# A neuropsychological instrument measuring age-related cerebral decline in older drivers: development, reliability, and validity of MedDrive

**DOI:** 10.3389/fnhum.2014.00772

**Published:** 2014-10-09

**Authors:** Paul Vaucher, Isabel Cardoso, Janet L. Veldstra, Daniela Herzig, Michael Herzog, Patrice Mangin, Bernard Favrat

**Affiliations:** ^1^Unit of Traffic Medicine and Psychology, University Center of Legal Medicine Lausanne–Geneva, University of GenevaGeneva, Switzerland; ^2^Unit of Traffic Medicine and Psychology, University Center of Legal Medicine Lausanne–Geneva, Centre Hospitalier Universitaire Vaudois, University of LausanneLausanne, Switzerland; ^3^Department of Neuropsychology, University of GroningenGroningen, Netherlands; ^4^Unit of Psychophysics, The Brain Mind Institute, École Polytechnique Fédérale de LausanneLausanne, Switzerland; ^5^Department of Ambulatory Care and Community Medicine, Centre Hospitalier Universitaire VaudoisLausanne, Switzerland

**Keywords:** aging, cognitive decline, measuring instrument, processing speed, psychometry

## Abstract

When facing age-related cerebral decline, older adults are unequally affected by cognitive impairment without us knowing why. To explore underlying mechanisms and find possible solutions to maintain life-space mobility, there is a need for a standardized behavioral test that relates to behaviors in natural environments. The aim of the project described in this paper was therefore to provide a free, reliable, transparent, computer-based instrument capable of detecting age-related changes on visual processing and cortical functions for the purposes of research into human behavior in computational transportation science. After obtaining content validity, exploring psychometric properties of the developed tasks, we derived (Study 1) the scoring method for measuring cerebral decline on 106 older drivers aged ≥70 years attending a driving refresher course organized by the Swiss Automobile Association to test the instrument's validity against on-road driving performance (106 older drivers). We then validated the derived method on a new sample of 182 drivers (Study 2). We then measured the instrument's reliability having 17 healthy, young volunteers repeat all tests included in the instrument five times (Study 3) and explored the instrument's psychophysical underlying functions on 47 older drivers (Study 4). Finally, we tested the instrument's responsiveness to alcohol and effects on performance on a driving simulator in a randomized, double-blinded, placebo, crossover, dose-response, validation trial including 20 healthy, young volunteers (Study 5). The developed instrument revealed good psychometric properties related to processing speed. It was reliable (ICC = 0.853) and showed reasonable association to driving performance (*R*^2^ = 0.053), and responded to blood alcohol concentrations of 0.5 g/L (*p* = 0.008). Our results suggest that MedDrive is capable of detecting age-related changes that affect processing speed. These changes nevertheless do not necessarily affect driving behavior.

## Introduction

In western Europe we expect the proportion of the population aged 60 years or more to double by 2075 compared to the present, rising from 21 to 42% (Lutz et al., 2008). Increased life expectancy at birth can be attributed to societal development, reduction of perinatal deaths for both newborns and mothers, a secure environment, and the decreased lethality of diseases. In developed countries, these improvements tend to leave normal aging as the major cause of death. This process is the result of chemical changes in cellular metabolism that increase the probability of dying, with age, even under optimal conditions (Harman, 2001). The same chemical changes are also believed to induce cerebral decline (Riederer et al., 2013). Therefore, the proportion of the population that will have to change their habits due to these alterations is bound to increase and cause some important societal changes that we need to anticipate (James et al., 2011).

We are, however, not all equal when facing cerebral decline in the context of normal aging; some of us are affected much sooner than others, without us completely understanding why (Hayden et al., 2011). Apparently, the process seems to affect most mammals and starts as early as during our twenties (Salthouse, 2009). Cognitive functions that are most affected are working memory, visual perception, and spatial orientation (Schaie, 1996). These can induce an important pejoration in older adults' quality of life (Deary et al., 2009).

Understanding the underlying mechanisms that could explain such differences remains a challenge for neuroscience (Grady, 2012). Animal research has allowed us to develop some explanations of the human brain aging process, but has also shown important limitations when investigating the consequences of cerebral decline for more complex behaviors (Gallagher and Rapp, 1997; Alexander et al., 2012). Indeed, when translating methods from animals to humans in age-related studies (Keeler and Robbins, 2011), one major difficulty is anticipating how individuals will adapt to changes that progressively emerge over periods that can stretch to over 50 years. Hence, to choose optimal behavioral measurements in aging research, we need to verify that these measurements are correlated to changes in behaviors occurring in a natural environment.

In developed countries, most human adults share a common skill that requires intensive training to acquire, is a complex executive task, and has elevated visual spatiotemporal constraints. This highly demanding task consists of driving a motor vehicle in open traffic. Functions required to drive safely are precisely those that have been shown to become impaired with age (Ryan et al., 1998; Anstey et al., 2005; Salthouse, 2010). This has made it possible for cognitive neuroscience and computational neuroergonomics (Liu et al., 2012) to model the underlying neural processing engaged during driving (Parasuraman and Wilson, 2008; Lees et al., 2010) and validate this model against driving difficulties (Rizzo, 2011; Aksan et al., 2012). These recent advances have us consider driving performance as the optimal choice for a reference standard to select and validate neuropsychological measures designed to detect age-related cerebral decline affecting older adults' mobility.

Epidemiological studies have shown that the relationship between age and road fatalities takes a U shape and older drivers are much more likely to be involved in a fatal accident than drivers aged 40 and above (Braver and Trempel, 2004; Eberhard, 2008; Langford et al., 2008). Those that fail an on-road driving test have an increased risk by 1.7 times (CI95% 1.3–2.2) of been involved in a crash with injury in the following 2 years compared to other aged drivers (Keall and Frith, 2004). However, when comparing fatalities occurring to occupants of other vehicles, we notice that this difference is mainly due to their own vulnerability (Braver and Trempel, 2004). Circumstances of accidents nevertheless differ with age. The older we get, the more likely accidents are to occur in daylight hours and following turning maneuvers, thereby provoking angled crashes (Ryan et al., 1998). With age, traffic violation becomes less frequent but lapses increase (Parker et al., 2000; Assailly et al., 2006; Classen et al., 2010) especially in lane maintenance, yielding, gap acceptance (Classen et al., 2010), and over-cautiousness (Dobbs et al., 1998).

Having physicians warn authorities about unfitness to drive prevents two patients in each thousand every year from injuring themselves while driving (4.76 vs. 2.73‰; *RR* = 1.45, CI95% 1.36–1.52) (Redelmeier et al., 2012). This however also leads to increased depression and out-of-home activity for those who ceased driving (Marottoli et al., 1997, 2000). Therefore, driving cessation is something that needs to be prepared for (Liddle et al., 2008), and means have to be put in place to help aging drivers to continue driving safely for as long as possible and also to decide when to stop (Edwards et al., 2009). Therefore, investigating the link between cerebral decline and driving behavior also has an important clinical application.

Using computed neuropsychological tests, it is conceptually possible to measure cerebral decline (Salthouse, 1996) but it is much more difficult to measure fitness to drive (Ball et al., 2006; Mathias and Lucas, 2009; Lin et al., 2013). To bridge this gap, it seems important to focus on measures that are known to be related to motor vehicle collisions, to have an idea whether these measures can detect changes from other conditions also known to affect driving performance, and to have a better understanding of underlying psychophysical properties of neuropsychological tests that are used. In neuropharmacology, when testing effects of drugs on driving, it is indeed recommended to calibrate effects of substances on those of alcohol (Owens and Ramaekers, 2009). Using driving performance and alcohol as references, this paper presents the cornerstone and the psychometric properties of an instrument designed to measure initial stages of age-related cerebral decline associated to fitness to drive.

## Objectives

The aim of this project is to develop and present a free, reliable, transparent, computer-based instrument capable of detecting the effects of aging on visual processing and cortical functions for research into factors affecting behavior and mobility in older adults. The project was planned in two phases: the development phase and the validation phase (Shapiro, 2006; Toll et al., 2008).

During the development phase (Study 1) we defined and obtained content validity for tasks to be integrated in an instrument measuring age-related cerebral decline, we developed the instrument interface including calibration and determination of the stepwise procedure, we adapted and standardized instructions, and we derived the scoring method. Results from this first phase are briefly presented in the next section when presenting this new instrument called MedDrive.

The validation phase provides psychometric properties of the developed instrument. This validation phase required four studies running from September 2012 to March 2014 to answer five objectives: Objective 1—Validation of the MedDrive score in predicting driving behavior (Study 2); Objective 2—Measuring the instrument's reliability (Study 3); Objective 3—Studying the instrument's underlying psychophysical properties (Study 4); Objective 4—Testing the instrument's responsiveness to alcohol (Study 5); and Objective 5—Testing the instrument's ability to model the effects of alcohol on simulator-based driving performance (Study 5).

Methods and results are presented separately for each study.

## Study 1—development of MedDrive

### Methods and results

#### Selection and description of tasks to be integrated into the instrument

Human performance in abstract reasoning, pattern recognition, and problem-solving, also known as fluid intelligence (Cattell, 1971), has been shown to decline with age due to age-related slowing of processing speed (Bors and Forrin, 1995; Salthouse, 1996; Zimprich and Martin, 2002), whereas crystallized knowledge is not affected (Salthouse and Babcock, 1991; Park et al., 2002). Given that age also affects other cognitive functions that are not directly linked to intelligence (e.g., dual tasking or task switching), Park et al. (2010) used the term “fluid processing” for all cognitive performance that requires both primary sensory processing and higher order cortical control. Tests that have been shown to require longer processing times are those that involve primary cortical sensory processing, task switching, inhibition of distractors, and short-term spatial memory (Alexander et al., 2012). These cortical functions have also all been shown to be associated to modifications in driving behavior (Reger et al., 2004; Mathias and Lucas, 2009; Silva et al., 2009). Relying on guidelines provided for neuropharmacological research (Berghaus et al., 1997) to test effects of drugs on driving, we chose to investigate the following cognitive functions: visual processing, attention shift, movement detection, susceptibility to distractors, and spatial working memory.

***Visual processing (Task 1)***. For visual processing, one computed task retained our attention: the useful field of view (UFOV) (Edwards et al., 2005). In aging research, the UFOV has been shown to be one of the best predictors of driving difficulties (Wood and Owsley, 2014). It provides a reliable quantitative estimate of temporal aspects of visual processing (Owsley, 2013). This test however has some major limitations. When using a standard 60 Hz screen renewal rate, its first subtask is subject to a floor effect. From our observations, the software was potentially unable to correctly measure the 50% threshold for 50.7% of older drivers aged 70 years or more. The final outcome is therefore often related to the limits of the instrument instead of to the participant's true capacities. This makes it difficult to observe changes between conditions for high performing participants. Second, if the participant responds correctly to all stimuli, the stepwise procedure stops at the fastest possible interval related to the screen renewal rate (usually 60 Hz or 16.7 ms) even if the last answers were given correctly purely by chance. Given that there is one chance in two of responding correctly without seeing the target, overestimating performance occurs frequently especially for those that perform well. The UFOV has been designed for clinical purposes and aims to detect drivers with mild cognitive impairment. It therefore does not meet our requirement for a more precise instrument that can be used in behavioral research. We therefore designed a new task using the same paradigm as the UFOV but increased the workload. This task was named the *visual recognition task* (Presentation 1, Supplementary Figure 1).

The task is to be done in a dimed room in the absence of reflections on the screen. Like for all other tasks, participants are placed at 60 cm from the screen. The task consists of three subtasks: the central visual processing subtask, the peripheral visual processing subtask, and the dual visual processing subtask. For all subtasks, participants are requested to focus on a central fixation cue and prevent themselves from doing any eye movement. Furthermore, in natural settings, the location of an important visual stimulus is often unknown prior to its appearance. It therefore seemed important to prevent participants from knowing which type of image was coming next. It was therefore decided to use an unpredictable random order for the three subtasks. We nevertheless included an option making it possible to test each subtask separately.

The central visual processing subtask consists of distinguishing which of two adjacent white rectangles might be different from a standard 13 mm high by 12 mm wide white rectangle. Rectangles are projected centrally side by side on the screen within a 75 arcmin visual angle. Rectangles can either be identical, or one of them can have rounded edges (1 mm radius), one of them can be darker (0.8 luminance), one of them can be placed horizontally instead of vertically, or one of them can be smaller (91% of normal size).

The peripheral visual processing task consists of identifying which of six white arrows on a black background is facing inwards. Arrows are 32 mm long and appear at 76 mm on either side of the center horizontally and at ±45°. The central side of the arrow is therefore placed eccentrically at 5.7°.

The dual visual processing task consists of simultaneously having to perform the central and the peripheral processing task. For each subtask, with a 60 Hz renewal rate, the stimuli is exposed for 16.7, 33.3, 50, 66.7, 83.3, 116.7, 150, 216.7, 283.3, 366.7, 483.3, 650, 850, 1133.3, 1500, 1983.3, 2633.3, 3500, or 4633.3 ms using a separate stepwise procedure for each subtask. A mask, preventing retinal impression, immediately follows each stimulus. Participants are then accorded as much time as they want to provide their answer. For each correct answer, the duration of the stimuli is shortened of one step, for each wrong answer, it is lengthened of one step. To account for the important learning effect that can occur the first time the task is done, the task ends after 60 stimuli only when all three subtask have shown at least three upper and three lower reversals that are included in a range of three consecutive possible values. The outcome for each subtask is measured as the geometrical mean of the adjusted logged duration of stimuli for each subtask after its first reversal. This value corresponds to the 50% threshold for correctly identifying the stimuli once adjusted for learning effect occurring during the task.

***Attention shift (Task 2)***. Inspired by Posner and Petersen's model of attention (Posner and Petersen, 1990), the attention network test (Fan et al., 2002) was developed to specifically measure processing speed when controlling for spatial cueing, temporal cueing, and distractors (Rueda et al., 2004; Posner, 2008). It has the advantage of providing an indication of the biochemical aspect of neural regulation during visual processing (Brown and Friston, 2013). The attention network test has been shown to measure different dimensions of attention than does the UFOV (Weaver et al., 2009). This approach nevertheless uses a factorial design that has some limitations given that it falsely assumes underlying cognitive functions not to interact one with another during the task (Macleod et al., 2010; Ishigami and Klein, 2011; McConnell and Shore, 2011). Furthermore, its utility for measuring the effects of cerebral decline in older adults is debatable. Older adults have been shown to perform better than younger adults when distracted by perceptual interference caused by items flanked on either side of a target (Wright and Elias, 1979). This benefit is only present when the target location compared to its distractors is known in advance, which is the case during the neural network test (Mahoney et al., 2010; Bugg, 2014). When measuring cerebral decline, a decision to maintain the filtering task is therefore questionable and we decided to exclude it and only maintain components related to attention shift. This also made it possible to increase the number of target locations, thereby increasing the task's difficulty. We named this task the *central cue attention task* (Presentation 1, Supplementary Figure 2).

In the central cue attention task, participants are requested to press on the space bar as quickly as possible as soon as they see a square appear at a random eccentric location (320 arcmin) on the screen. These white squares on a black background are 4.5 mm wide and appear 56 mm away from the center of the screen. Three random conditions can precede the appearance of the target cue. The central fixation cue, that serves as a reference and prevents participants from moving their eyes, can be replaced by a flashing star that warns them of the imminent appearance of the target cue (alerting condition), or it can be replaced by an arrow indicating where the next square will appear without them knowing when it will appear (orientation condition), or it can simply remain as it is (neutral condition). Response time is measured 32 times for each condition. Each response time is then adjusted for learning effect using linear regression after removing the three worst measures to account for slips. The outcome for each condition is measured as the average adjusted response time. Orientation gain is measured as the difference between neutral and orientation condition and alerting gain is measured as the difference between neutral and alerting condition.

***Movement detection with distractors (Task 3)***. When investigating effects of distractors we decided to improve existing models and integrate movement detection instead of shape discrimination in a third task (Task 3). In this task, a cue appearing at a random interval indicates one of eight square blocks in which lines are moving in a random direction. As soon as the direction is detected and distinguished from the movement of adjacent blocks, the participant has to press on the keyboard's space bar and can then take the time they require to provide their answer. This task was called the *movement detection task* (Presentation 1, Supplementary Figure 3).

In this task, eighty 10 mm sided white squares with two vertical and two horizontal 1 mm black lines appear in a 160 by 160 mm space. A 12 mm long arrow appears centrally indicating one of the eight adjacent squares. Simultaneously, lines within each square will then move in one of four directions taking 1 s to move from one side to another. The direction of the movement is defined randomly for each square conserving a ¼ ratio for the number of squares with similar movement. Participants are asked not to move their eyes. The task ends after 40 trials with correct answers. Linear regression is used to measure learning effect occurring during the first 30 measures after removing the four worst measures to account for slips. The outcome is the average response time adjusted for learning effect occurring during the test.

***Spatial working memory (Task 4)***. The fourth task (Task 4) investigates spatial working memory (Olivers et al., 2011), known to be affected by age but deficits in which only manifest themselves during tasks with important workloads (Klencklen et al., 2012). We also wished to integrate an indication of temporal decay regarding which controversies exist concerning age-related decline (Barrouillet et al., 2011). This *spatial working memory task* therefore consists of having to remember the location of the first and last crosses appearing in a series of randomly located crosses ranging from two to seven in number (Presentation 1, Supplementary Figure 4).

A gray cross is placed at the center of the screen and remains there during the entire trial to serve as a spatial reference. After 1 s, a white cross, constituted of two 0.5 mm thick and 9 mm long white lines, appear at a random location within an invisible 80 mm radius circle before disappearing after 1.5 s. The participant is requested to remember where this first cross was located. The second cross will appear at a random location within the circle but not within the two adjacent 30° sections on each side of the section in which the first cross had appeared. This is to prevent participants confusing the location of this cross with the one of the first cross they have to remember. Crosses continue appearing until the sequence stops. Participants are then requested to indicate with the screen cursor where they think the last cross was before also reporting where they think the first cross was. The random number of appearing crosses ranges from two to seven. The distance from the true position to the reported position for the first and last cue is registered. This procedure is repeated thirty times. The three worst measures for the first and last cue are removed. To normalize the range of measures, values are logged transformed, and the mean logged value is calculated after adjusting for learning effect. Outcomes therefore correspond to the geometrical mean precision for locating the first and last cue. Memory decay is measured as the slope of the loss of precision for the first cue depending of the number of crosses that appeared.

#### Content validity

**Figure 1:**
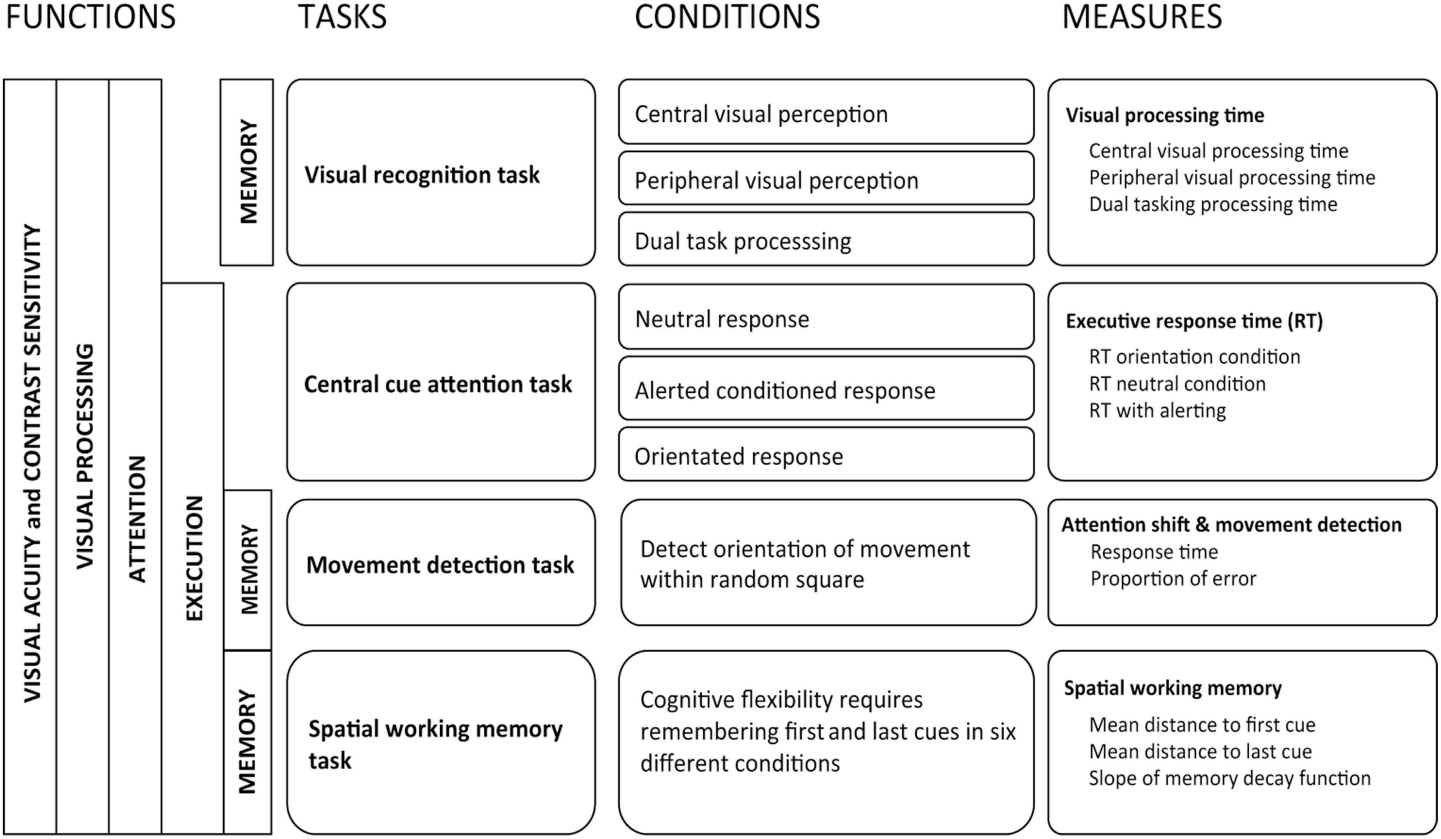
**Description of tasks and outcomes included in MedDrive2**.

A panel of three international experts—one clinician and researcher specializing in fitness to drive assessment, one neurologist specializing in cognitive neuroscience, and one psychologist specializing in drug-related effects on driving—was asked to provide opinions regarding the four tasks considered for inclusion in MedDrive (Figure 1). The experts were asked whether they believed each test to be suitable for testing cerebral decline or the effects of drugs on older adults. They were also invited to criticize the construction of the instrument and argue for and against additional measures. Experts were provided with a written detailed description of the aim of the instrument and the planned task. A face-to-face was then organized with each expert to answer their questions and find potential solutions that were implemented before interviewing the next expert. Taking their feedback into account, the instrument was revised and all experts were then asked to assess the final modified versions until a consensus was found.

#### Interface development, calibration, and determination of the stepwise procedure

For the exposed images to conserve their characteristics at a distance of 60 cm, we integrated a method in the software to adapt the number of pixels to be activated depending of the screen resolution. Instructions and training for each task were also integrated in the software.

For the visual processing task (Task 1), images were exposed 500 times at 50 ms and then modified until the prevalence of errors was equivalent for all image types. To achieve this, 8500 measurements were needed. To estimate the psychometric function of visual processing separately for each image type, three healthy young volunteers repeated measurements 500 times for each of the following exposure durations: 217, 150, 117, 83, 67, 50, 33, and 17 ms. These results showed slight discrepancies of psychometric functions with a 50% threshold ranging from 20 to 59 ms depending on features. The main differences concerned function slopes that were steeper for shape, orientation, and size than for contrast and identity. We also noted that sensitivity to contrast in the central visual processing subtask and shape size in the peripheral visual processing subtask were age-related and explained an important part of the function shift between features in younger adults.

We tried to optimize the stepwise procedure measuring a temporal threshold for visual processing to increase precision and reduce the number of measurements required. Given that important learning effects were observed, a Bayesian approach assuming the underlying function to be stable was abandoned (Klein, 2001). We also decided to only focus on the 50% threshold that was more or less centered at the same point for all features and renounced estimating the slope of the psychometric function. Our method therefore principally relies on the linear symmetrical aspect of the slope around the central part when temporal intervals are set on a log scale. In other words, given the probability of correctly answering purely by chance was significantly lower than 50%, we were able to simply increase the temporal intervals of a single step for wrong answers and go down by one step if answers were correct until the function became stable around a central point. Our method is not subject to the same vulnerability to high performing results occasioned by chance as is the UFOV for two reasons. First, we use stimuli for which there is only one chance out of five to guess correctly. This makes it much less likely to successively answer consecutively more than three times in row just by chance. Second, the procedure does not stop once the fastest threshold is reached. It continues at that speed for at least three repeated measures.

We then had five young adults do all four tests three times to identify the nature of the learning function during each test. For all tasks, the function was logarithmic with high learning effects during the first measurements and an optimal gain within the 30 initial measurements. This made it possible to integrate a regression method estimating each individual's learning function thereby adjusting the final results for learning effects occurring during the session. We also identified the number of outliers to exclude, how to transform values to be normally distributed, how to adjust observed values for learning effects, and which additional outcomes to provide to make it possible to detect abnormal responses.

We also tested the effect of the distance to the screen with 17 volunteers and noticed that performances remained very similar for distances of 33, 66, or 99 cm (*F*_df(3)_ = 2.22, *p* = 0.143). To simplify the procedure, participants are now therefore required to sit at arm's length from the screen when performing tasks.

MedDrive was programmed with a user-friendly interface designed for both research and clinical purposes (www.meddrive.org). It was programmed on Qt in C++ language and is designed for personal computers that run either Windows or Mac OS. Feature properties are maintained independently of screen size and can even be used with screens that have pixels that are not square. Tasks have been designed for a screen renewal rate of 60 Hz but performances at lower temporal intervals remain possible down to 8.3 ms (120 Hz).

#### Testing, adapting instructions, and the derivation of the scoring method

The instructions provided to older drivers for performing tasks were, for each task, tested and modified by having 106 older drivers perform a random selection of two of the four MedDrive tasks. Drivers were selected from participants in a driving refresher course organized for senior drivers (age ≥70 years) by the Swiss Automobile Association between May and September 2012. The instructions were not therefore consistent across all trials in the derivation set. This is however not an issue as we only began the measures once participants were able to perform the task as instructed. The main improvement was therefore the reduced time we needed to provide the instructions and training.

From the data collected from these 106 drivers, for each task we selected the outcome that was most associated to age (*p* < 0.2). We then defined the number of measurements required for each task. The stepwise procedure in Task 1 ends when three upper and three lower reversals fall within three intervals. For task 2 and 3 we initially had the participants continue tasks after the 30th measurement until their last 10 measures were within 5 ms of all the previous measurements. For Task 2, we noticed this was achieved for more than 90% of the participants before the 30th measure. We however had outliers due to the fact some participants were not pressing the space bar hard enough and would do so a second time after more than 1–2 s. For this reason it was decided to suppress the two slowest measurements and set the total number of measurements to 32. For task For task 4, we initially ran 40 series and noticed that the measures became stable enough from the 30th measurement onwards. We then computed outcomes and retained the 10th and the 90th percentile for each outcome on both older and younger drivers. This made it possible to define the expected range of possible values (Table 1). Summary measures were then transformed to a score ranging from 0 to 1—1 corresponding to optimal performance. Number of lapses during each task, alerting and orientation gain, congruent and incongruent movement detection in task 3, and spatial decay function were not associated to driving behavior or age and were not accounted for to construct the overall composite score. MedDrive's Matlab code and instructions are available at www.meddrive.org.

**Table 1:** **Retained MedDrive outcomes from derivation set**.

	**Transformation Score [0–1]**	**Range of expected values**		
		**Retained**	**Observed (10th–90th percentile)**	
			**Age < 40 (*n* = 27)**	**Age > 65 (*n* = 74)**
**VISUAL RECOGNITION TASK (TASK 1)**				
Central visual processing	1 − [(ln(*t*) − 2.833)/3.564]	17–600 ms	59–175 ms	137–520 ms
Peripheral visual processing	1 − [(ln(*t*) − 2.833)/3.852]	17–800 ms	17–139 ms	92–715 ms
Dual tasking	1 − [(ln(*t*) − 2.833)/5.328)	17–3500 ms	186–1258 ms	1215–3255 ms
**CENTRAL CUE ATTENTION TASK (TASK 2)**				
Execution with orientation cue	1 − [(*t* − 150)/350]	150–500 ms	249–348 ms	282–441 ms
**MOVEMENT DETECTION TASK (TASK 3)**				
Movement detection with attention shift	1 − [(*t* − 300)/1500)	300–1800 ms	448–1064 ms	743–1648 ms
**WORKING SPATIAL MEMORY TASK (TASK 4)**				
Working memory—1st cue	1 − [(*d* − 1)/29]	1–30 mm	7.8–19.5 mm	11.8–25.0 mm
Working memory—last cue	1 − [(*d* − 1)/29)	1–30 mm	7.0–12.8 mm	7.4–20.6 mm

*The measured threshold values were transformed to range from zero to one. The range of expected values was estimated from healthy populations. Outcomes below the lower bound of retained expected values were attributed the score 1 (optimal response), and values above the upper bound of retained values the score of 0. High scores therefore indicate better performance*.

## Study 2—validation study

### Methods for study 2

#### Study population

In collaboration with the national Driver and Vehicle Licensing Agency and the Swiss Automobile Club, we wrote to all drivers (*N* = 7867) who had reached their 70th year and were residents of western Lausanne, Vevey, Montreux, Aigle, and Entremont, inviting them to participate in a refresher course on driving competencies. Four hundred and four subscribed (5.1%). In this refresher course, all participants were then offered the opportunity to participate in this study. They were informed of the study both orally and in writing. A researcher called them to obtain their consent and schedule a 100-minute appointment. To be included, participants had to hold a valid Swiss driver's license, be aged 70 years or more, and be community-dwelling (i.e., not in assisted living or a nursing home). Their valid Swiss driver's license had to be non-restricted and they had to be capable of driving without assistance and not have a known diagnosis of dementia, Parkinson's disease, sequels to brain injury (e.g., trauma, stroke), or other important disorders known to affect cognition.

**Figure 2:**
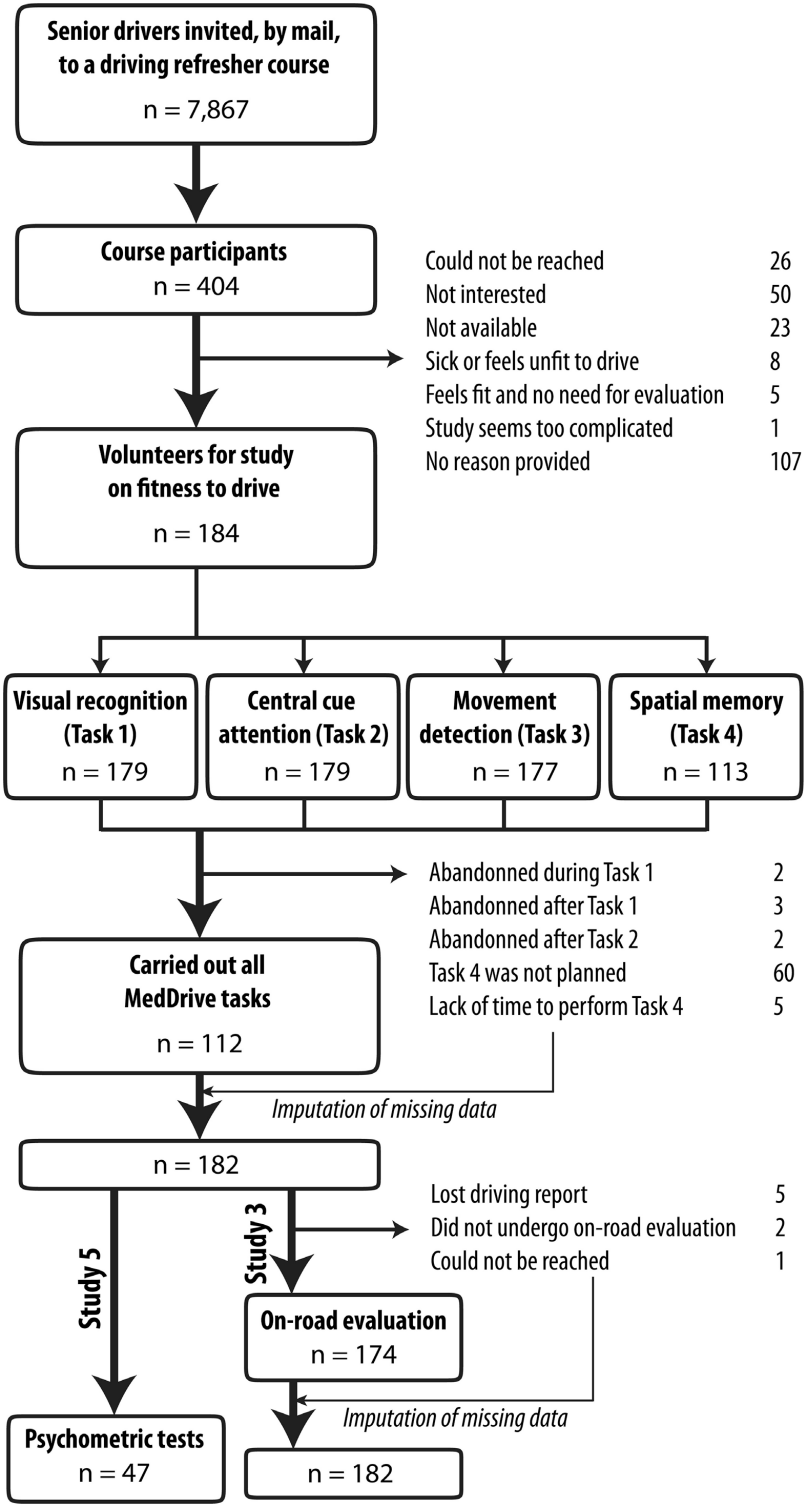
**Flow chart for selection of older drivers for studies 3 and 5**. Participants were volunteers from a driving refresher course. Close to one senior driver out of two (45.5%) agreed to participate. *n*, sample size.

Between September 2012 and September 2013, 182 older drivers were recruited. The method used to select drivers and reasons for not being included are provided in Figure 2. Characteristics of selected older drivers are provided in Table 2.

**Table 2:** **Characteristics of older home-dwelling drivers (Study 3: *n* = 182)**.

	**% (*n*)**
Age (years)	
67–69 years	1.6% (3)
70–74 years	48.4% (88)
75–79 years	29.1% (53)
80–84 years	15.9% (29)
≥85 years	5.0% (9)
Gender (% male)	66.5% (121)
Education (% <12 years)	34.6% (63)
**VISION**	
**Visual acuity (Snellen chart)**	
<0.6 dec	1.7% (3)
0.6–1.0 dec	53.9% (98)
>1.0 dec	43.4% (79)
Visual field (<140°)	0.6% (1)
**Contrast sensitivity (MARS)^†^**	
≤1.64 logCS	8.2% (15)
1.68–1.72 logCS	47.2% (86)
≥1.76 logCS	37.4% (68)
**FUNCTIONAL MOBILITY**	
Timed up-and-go task > 12 s	8.2% (15)
**COGNITIVE STATE**	
**MoCA (points [0–30])**	
0–18 points	0.5% (1)
19–25 points	29.1% (53)
26–28 points	42.9% (78)
29–30 points	27.5% (50)
**DRIVING**	
**Distance driven per week (km)**	
≥200 km	51.6% (94)
100–199 km	38.5% (70)
50–99 km	8.2% (15)
<50 km	1.7% (3)
**History of accidents (previous 2 years)**	
All types with material damage	26.4% (48)
Responsible for damage to other(s)	4.9% (9)
Injured	1.1% (2)
**On-road driving performance^†^**	
Excellent	44.5% (81)
Good	33.5% (61)
Moderate	15.4% (28)
Poor	4.5% (12)
**Driving self-restriction score [0–20]**	
None	34.6% (63)
1–3	43.4% (79)
4−	15.9% (29)
>6	6.1% (11)

^*^MARS was introduced after the eleventh participant had been included.

† *On-road performance was imputed for 8 drivers. CS, contrast sensitivity; MARS, mars contrast sensitivity test; MoCA, Montreal cognitive assessment*.

#### Procedure

Participants self-reported their age, history of accidents, driving habits, medication and other drug consumption. These were then classified as class I, II, or III depending of their known effect on driving (Riche et al., 2009). Cognitive status was investigated using the MoCA. We tested contrast sensitivity using the MARS, and visual field and acuity using a dedicated portable device (Essilor, Visiotest). Participants then were requested to wear their optical correction lens to perform the computed tests.

#### Computed tests

Lights were dimmed to prevent any reflections on the screen. Participants sat at arm length from the computer screen using their optical corrections if required. Tasks were projected on a 22-inch screen with a 60 Hz refresh rate. Screen brightness was set to the maximum.

***MedDrive***. All older drivers performed MedDrive tasks in the same order from the first to the fourth task. All participants received the same standardized instructions and training session included in the software. Details on the four tasks are provided in Section Study 1 - Development of MedDrive of this article.

***Useful field of view (UFOV)***. For concurrent validity, we used the UFOV, one of the most widely used visual processing speed tests in traffic psychology (Ball et al., 1988; Edwards et al., 2006). This task measures the ability to rapidly visually detect and localize targets. It also investigates disengagement from a previous goal (dual tasking) and visual search. The first version measuring performances at different angles of eccentricity was later abandoned for a fixed angle. We used this fixed angle version that included three subtasks (version 6.1.4). The first subtask consists of measuring the threshold duration needed to correctly identify an appearing vehicle in the center of a screen (processing speed). The second subtask consists of measuring the threshold duration needed to correctly identify an appearing vehicle in the center of a screen and the location of a simultaneously appearing vehicle at a 10° eccentric angle from the center (divided attention processing speed). The third subtask is identical as the second apart for the addition of triangular distractors making it more difficult to identify the location of the eccentric vehicle (selective attention processing speed). Contrarily to instructions, when participants required more than 500 ms to perform subtask one or two, we still had them perform the following subtasks.

#### On-road evaluation

Routes were standardized for participants from the same region. They were sufficiently difficult for lapses to occur, and long enough (≈45 min) to assess the effects of sustained attention. Routes included urban and rural sections, secondary and principle roads and highways, simple and complex intersections, “roundabouts” (circular intersections with changing on-road priorities), traffic signals, and complex lane selections. The Swiss National Council for Road Security validated the routes. All study participants performed the on-road evaluation in their own vehicle with a driving instructor on the passenger seat. Twelve driving instructors participated in the study. They were either self-employed or were employees of the Swiss Automobile Club. They were all certified by the Swiss National Council for Road Security with a specific diploma for managing older-driver instruction. Driving instructors were blinded to the results from the psycho-medical evaluation and reported their “gestalt” evaluation of driving performance as “good” or “sufficient” for the following criteria: respecting road regulations, handling vehicle, speed adaptation, correct position on the road, comfort, behavior toward other road users, observation, and anticipation. Driving competencies were summarized as excellent (no lapse), good (lapses reported for one or two items), moderate (lapses reported for three to five items), or poor (lapses reported for six to eight items). This scoring method was verified using principle component analysis and Rasch analysis on a set of 445 older drivers thereby confirming its unique dimension (Eigenvalue = 5.1) and good fit to an overall trait (R1c = 12.2, *df* = 14, *p* = 0.565).

#### Driving self-restriction score for older drivers

No validated methods were available to measure driving self-restrictions. A literature review had us identify 13 questions on strategic compensations (Irwin, 1989; Raitanen et al., 2003; Ragland et al., 2004; Baldock et al., 2006; Molnar and Eby, 2008; Ross et al., 2009; Blanchard and Myers, 2010). These were reformulated for drivers to place their response on a five-level Likert scale ranging from “useless” to “indispensable.” Data were collected from 445 senior drivers. We then relied on factorial analysis using polychoric correlation to construct the measuring scale. Number of factors was defined by principle component analysis. Iterated principal factor with orthogonal rotation was then used to structure factors. Those with an eigenfactor higher than 0.6 were excluded from the model and the analysis was run over again. This resulted in identifying five items derived in a single factor: restrict driving to known roads, avoid driving on highways, avoid driving in the dark, avoid driving in dense traffic, and avoid driving in fog. The internal coherence of the scale was acceptable (Cronbach alpha = 0.680). We then used bidimensional polytomous Rasch model analysis to verify that each score within each item was linearly associated to the overall trait. The score has good discrimination properties for distinguishing drivers with medium to high traits but not so good for those with low to medium traits. This is mainly due to the high prevalence of drivers who have not yet adopted driving compensation strategies.

#### Statistical analysis and ethical standards

We used confirmatory factor analysis and SEM with linear regression to validate the model that issued from the derivation set. Data from older drivers who accomplished all four tasks were used to define weights of each of the seven measures used to compute the MedDrive score. Goodness-of-fit indices were chosen prior to the analysis. We then used simple imputation with linear regression (using available MedDrive measurements, gender, and driving performance score) to replace missing measures, thereby making it possible to compute the MedDrive score for all participants. We used linear regression to test the association of the MedDrive score and its constituents to driving performance and driving self-restrictions. Given determinants and outcomes were all expected to be correlated, we did not use Bonferoni adjustments for *p*-values. Significance level was set at *p* < 0.05. The analysis was pre-programmed on STATA 12 and then executed.

All patients gave their written informed consent to participate. Ethical approval was obtained from official state Biomedical Ethics Committees under reference number CE157/2011. All steps in developing MedDrive were conducted in accordance with the principles of the declaration of Helsinki (6th revision, Seoul).

### Results from study 2

#### Association to age

Older driver's age ranged from 67 to 91 years with a median age of 75 years (mean = 76 years, *SD* = 4.8). For these older drivers, MedDrive score decreased linearly of 5.2 points for every decade (*n* = 182, *R*^2^ = 0.041, *p* = 0.006). This association was maintained after adjusting for education level, visual acuity, and contrast sensitivity (*p* = 0.032). When adding measures from younger drivers (age < 40 years), a similar loss of 4.9 points (95% CI 3.8–6.0) per decade was also observed. It is also worthwhile to note that even if large differences of performance on MedDrive were observed between younger and older drivers (mean = 65.3 points vs. 43.8 points), the variance between individuals remained the same (*SD* = 11.9 vs. *SD* = 12.3).

Without the time needed to provide instructions (≈10–15 min), older drivers took 23 min (ranging from 16 to 36 min) to perform all four tasks. The visual recognition task (Task 1) was the one that took the longest (9′ 40″), followed by the spatial memory task (Task 4; 8′ 06″), the movement detection task (Task 3; 4′ 22″), and the central cue attention task (Task 2; 4′ 07″).

#### Construct validity

**Figure 3:**
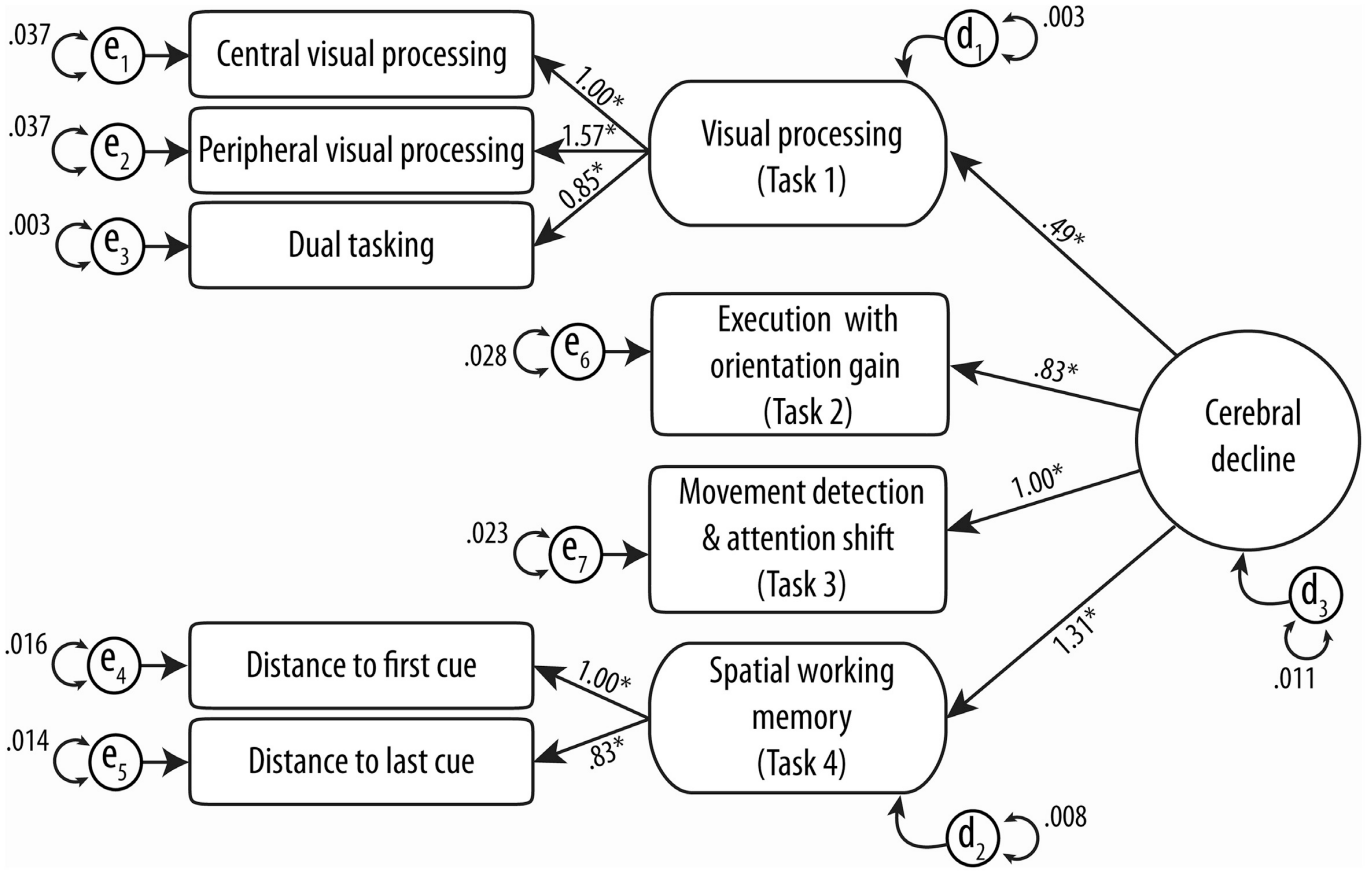
**Confirmatory structural equation modeling of MedDrive's theoretical construct**. Circles and rounded-cornered squares represent latent variables. Numbers above straight lines correspond to weights of score for MedDrive outcomes to best model “cerebral decline.” e_n_ models residual errors for observed variables and d_n_ models the same errors for latent variables. ^*^Identifies variables that contribute significantly (LRtest *p*-value < 0.05) to the model.

One hundred and twelve drivers completed all four MedDrive tasks (61.5%). The structure of our model and its validation as a unique construct of “cerebral decline” are illustrated in Figure 3. Structural equation modeling confirmed that each measurement contributed significantly (LRtest; *p* < 0.009) to the overall latent trait we assumed to be related to cerebral decline.

Different goodness-of-fit statistics revealed the MedDrive score to perform very well in defining this studied trait (Table 3). The only index below expectations is the goodness-of-fit statistics (GFI). This GFI is however known to be unstable with an important number of parameters and relatively small sample size (Hooper et al., 2008; Westland, 2010). The measurement that best fitted the overall trait we have called “cerebral decline” was the latent measurement corresponding to working memory (*R*^2^ = 0.720; Task 4). It was followed by the latent measurement corresponding to visual perception (*R*^2^ = 0.474; Task 1), the single measurement from the movement detection task (*R*^2^ = 0.329; Task 3), and finally the measurement for simple executive response time with orientation cue (*R*^2^ = 0.217; Task 2). We noted that the central visual processing subtask without the other two measurements of visual perception had the most difficulties in fitting the overall trait (*R*^2^ = 0.136).

**Table 3:** **Goodness of fit of derived MedDrive score in determining “Cerebral Decline”**.

**Fit indexes**	**Observed values (*n* = 112)**	Recommended values^*^
*X*^2^_(*df* = 12)_	13.8 (*p* = 0.314)	*p* > 0.05
RMSEA_(CI95 %)_	0.037	<0.07
CFI	0.987	≥0.95
TLI	0.977	≥0.95
GFI	0.809	≥0.95
SRMR	0.041	<0.08

* *(Hooper et al., 2008) CFI, comparative fit index; GFI, goodness-of-fit statistics; RMSEA, root mean squared error of approximation; SRMR, standardized root mean square residual; TLI, Tucker–Lewis index; X^2^, Chi square test*.

#### Validity against driving behavior

Data were considered to be missing at random and we imputed missing measures to compute the MedDrive score for all 182 participants. MedDrive score ranged from 8.7 to 65.0 points with a median of 43.0; zero corresponding to poor performances in all tasks and 100 to optimal results expected from young, healthy subjects. The mean score was 41.6 (*SD* = 12.2). Values were distributed normally (skewness = −0.605, kurtosis = 3.120). We also noticed that the MedDrive score was correlated to cognitive impairment measured by the MoCA (*R*^2^ = 0.152, *p* < 0.001), and also, but to a lesser degree, to age (*R*^2^ = 0.041, *p* = 0.006).

**Figure 4:**
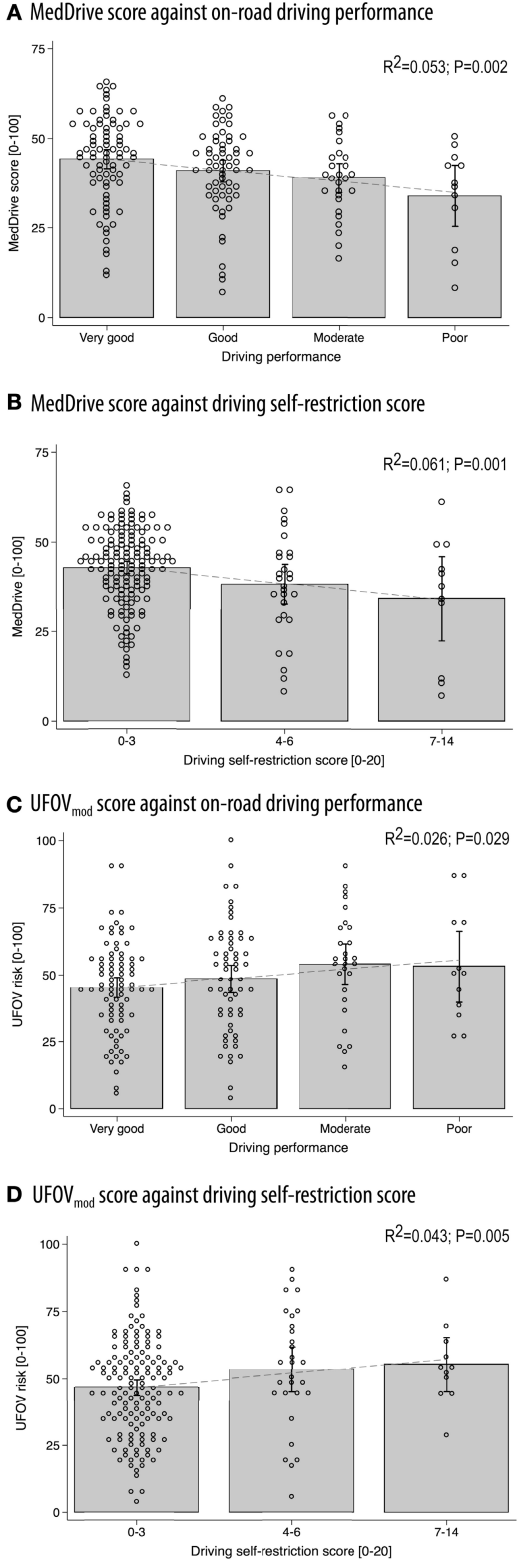
**Concurrent validity between MedDrive and the UFOV in predicting on-road driving performance or driving self-restrictions**. Correlations between MedDrive and on-road driving performance **(A)** and driving self-restriction score **(B)** on 182 older drivers. Concurrent validity was evaluated by providing the UFOV's correlations to the same measures of driving behavior **(C,D)**. Dots correspond to each individual observation. Gray blocks' upper borders correspond to mean observed score values. Interval bars represent 95% CI. Dotted lines represent the best fit regression line. UFOV_mod_ was generated from log transformed values of visual processing, divided attention, and selective attention using SEM. This overall score was then transformed to range from 0 to 1. This method fitted driving behavior better than did the standard UFOV risk category method and made comparison with MedDrive more even. *P*-values correspond to LRtest statistics for *R*^2^ = 0. UFOV, useful field of view; *R*^2^, coefficient of determination; SEM, structural equation modeling.

As expected, the magnitude of the association to on-road driving performance was weak (*R*^2^ = 0.053, *p* = 0.002). We were nevertheless satisfied to observe a linear decrease of mean MedDrive scores with increased misjudgments and errors observed during the on-road evaluations (Figure 4A). MedDrive was also capable of predicting the importance accorded by drivers to their driving self-restriction (Figure 4B, *R*^2^ = 0.061, *p* = 0.001). We also observed an absence of residual confounding that could have appeared with age. Indeed, the magnitude of the associations were not affected after adjusting for age, and age did not contribute to improving the prediction of driving behavior (driving performance: ß_adj_ = −1.71 vs. ß = −1.74, *p* = 0.855; driving self-restriction score: ß_adj_ = −5.79 vs. ß = −5.41, *p* = 0.255). Finally, when breaking down the MedDrive score to its constituents, all task outcomes were significantly associated to either the on-road evaluation or to the driving self-restrictions score (Table 4).

**Table 4:** **Computed tasks' associations to driving behavior (*n* = 182)**.

	**Median [range]**	**On-road evaluation [Very good, good, moderate, poor]**			**Driving self-restriction score [0–20]**		
	***n* = 182**	**ß coefficient**	***R*^2^**	***p*-value**	**ß coefficient**	***R*^2^**	***p*-value**
**MedDrive (SCORE 0–100)**							
Visual processing (Task 1)	22.0 [0–67.3]	ß = −1.628	*R*^2^ = 0.062	*P* = 0.001	ß = −2.716	*R*^2^ = 0.020	*P* = 0.055^*^
*Central visual processing*	28.8 [0–75.7]	ß = −0.770	*R*^2^ = 0.029	*P* = 0.021	ß = −0.719	*R*^2^ = 0.003	*P* = 0.463^*^
*Peripheral visual processing*	23.0 [0–98.1]	ß = −0.886	*R*^2^ = 0.047	*P* = 0.003	ß = −1.511	*R*^2^ = 0.016	*P* = 0.088^*^
*Dual tasking*	10.8 [0–43.0]	ß = −1.282	*R*^2^ = 0.013	*P* = 0.122^*^	ß = −5.754	*R*^2^ = 0.031	*P* = 0.017
Execution with orientation cue (Task 2)	41.7 [0–75.3]	ß = −0.726	*R*^2^ = 0.021	*P* = 0.050^*^	ß = −2.688	*R*^2^ = 0.034	*P* = 0.013
Movement detection with attention shift (Task 3)	39.8 [0–76.2]	ß = −0.874	*R*^2^ = 0.033	*P* = 0.014	ß = −3.382	*R*^2^ = 0.054	*P* = 0.002
Working spatial memory (Task 4)	62.2 [0–85.8]	ß = −0.814	*R*^2^ = 0.019	*P* = 0.066^*^	ß = −3.343	*R*^2^ = 0.037	*P* = 0.009
*Working memory—1st cue*	61.0 [0–87.0]	ß = −0.447	*R*^2^ = 0.008	*P* = 0.240^*^	ß = −2.597	*R*^2^ = 0.030	*P* = 0.019^*^
*Working memory—last cue*	64.1 [0–100]	ß = −1.032	*R*^2^ = 0.031	*P* = 0.017^*^	ß = −3.036	*R*^2^ = 0.031	*P* = 0.017
**MedDrive score**	43.0 [7.4–66.0]	ß = −1.737	*R*^2^ = 0.053	*P* = 0.002	ß = −5.410	*R*^2^ = 0.061	*P* = 0.001
**UFOV (ms)**							
*Visual processing (ms)*	16.7 [16.7–270]	ß = 0.649	*R*^2^ = 0.021	*P* = 0.048^*^	ß = 1.638	*R*^2^ = 0.016	*P* = 0.088^*^
*Divided attention (ms)*	.117 [16.7–500]	ß = 0.355	*R*^2^ = 0.012	*P* = 0.143^*^	ß = 1.893	*R*^2^ = 0.040	*P* = 0.007
*Selective attention (ms)*	263 [67–500]	ß = 0.864	*R*^2^ = 0.030	*P* = 0.020	ß = 1.800	*R*^2^ = 0.015	*P* = 0.099^*^
**UFOV risk categories [0**–**5]**	1 [0–5]	ß = 0.632	*R*^2^ = 0.026	*P* = 0.028	ß = 1.860	*R*^2^ = 0.027	*P* = 0.027
**UFOV_mod_ score (0–100)**	49.7 [3.7–100]	ß = 0.780	*R*^2^ = 0.026	*P* = 0.029	ß = 2.920	*R*^2^ = 0.043	*P* = 0.005

*Scores can be interpreted as percentages of full capacity. Measurements from UFOV were transformed to range from 0 to 1 making it possible to compare coefficients one to another across the same driving score. The ß coefficient corresponds to the average observed increase on the driving score between minimum and maximum testing values. For goodness of fit, we used the coefficient of determination R^2^ indicating the proportion of shared variance. It ranges from 0 to 1, 1 been a perfect fit. P-values from likelihood ratio tests (LRtest) indicate the probability of the ß coefficient being null. ß, coefficient of regression; R^2^, coefficient of determination; UFOV, useful field of view*.

* *Was not significant when only accounting for values without imputation*.

We observed those with a MedDrive score below 25 points to have a three-fold increased risk (CI95% 0.90–10.2; *p* = 0.070) of performing poorly in the on-road evaluation, using such a cut-off point would have clinicians falsely assuming five patients out of six to be unfit to drive. It would also have them only detecting one poorly performing driver out of four. Spending 45 min to obtain such a result is not worthwhile. Therefore, it appeared important to investigate whether the movement detection task (Task 3) could replace the composite score that includes all four tasks. We therefore did a *post-hoc* analysis to measure its predictive ability. The movement detection task (Task 3) was able to explain 54.5% of the variance of the overall composite score. Those with values below 25 points had a non-significant increase in risk of 1.7 (CI95% 0.53–5.3, *p* = 0.383). Even if the test would detect one poorly performing driver out of three, it would also unfortunately also falsely assume nine drivers out of 10 to be unfit to drive.

#### Concurrent validity against the UFOV

Compared to MedDrive, UVOF risk categories performed half as well in predicting driving performance (*R*^2^ = 0.026 vs. 0.053) or driving self-restrictions (*R*^2^ = 0.027 vs. 0.061). Combining all three measures from the UFOV, using SEM (UFOV_mod_) did not improve the UFOV's prediction of driving behavior (*R*^2^ = 0.026, *p* = 0.029) but improved its ability to predict driving self-restrictions (*R*^2^ = 0.043, *p* = 0.005). The visual perception score from MedDrive performed better in predicting driving performance than did the UFOV_mod_ (*R*^2^ = 0.062 vs. 0.026, *p* = 0.032) but did not do any better in predicting driving self-restrictions (*R*^2^ = 0.021 vs. 0.043, *p* = 0.117). Detailed results for the UFOV composite score are provided in Figures 4C,D.

## Study 3—reliability study

### Methods for study 3

Seventeen younger drivers (age ranged from 23 to 39 years), who were also volunteers for study 5, agreed to participate to this reliability study and performed all MedDrive tasks five times. The first measurements were carried out on one of our labs PC with a 22-inch LCD screen. All other measurements were carried out on participants' personal home computers after they had downloaded and installed the software themselves. Participants were requested to repeat measurements at home in similar conditions than those in our laboratory. They were told to only run the tests if they knew they would not be disturbed during the next 15 min, that they did not feel more fatigue than usual, and that they had not consumed any psychotropic drugs during the previous 24 h. Once they had finished their five sessions, they then Emailed the recorded crude data file to the study research staff for their results to be analyzed. Reliability was measured using two-way mixed single measure intraclass correlation coefficients (ICC_3,1_).

### Results from study 3

MedDrive scores ranged from 35.2 to 77.0 points. Repeated-measure reliability for each MedDrive outcome is provided in Table 5. MedDrive's composite score had an overall ICC_3,1_ of 0.852 from the first measurement onward. Compared to the first session, participants did better at the second session (65.3 points vs. 68.7 points; *p* = 0.932). From the second onwards, we then observed a linear progression of the mean score with an improvement of 1.3 points (CI 95% 0.8–1.8) between each session.

**Table 5:** **Reliability of measurements in research settings with healthy adults**.

	**Repeated measure reliability**	
	**ICC(3,1) [CI95%]**	
	**All measures (*N* = 85, *n* = 17)**	**From 4th measure onward (*N* = 34, *n* = 17)**
**VISUAL RECOGNITION TASK (TASK 1)**		
Visual processing	0.838^†^ [0.717–0.927]	0.856 [0.713–0.940]
*Central processing*	0.420^†^ [0.221–0.665]	0.477 [0.181–0.740]
*Peripheral processing*	0.892 [0.803–0.953]	0.938 [0.867–0.975]
*Dual task response*	0.594^†^ [0.397–0.790]	0.587 [0.309–0.806]
**CENTRAL CUE ATTENTION TASK (TASK 2)**		
Executive response with orientation gain	0.561^†^ [0.361–0.769]	0.697 [0.441–0.859]
**MOVEMENT DETECTION TASK (TASK 3)**		
Movement detection with attention shift	0.879^†^ [0.782–0.947]	0.959^†^ [0.911–0.984]
**SPATIAL MEMORY TASK (TASK 4)**		
Spatial working memory	0.540 [0.338–0.754]	0.792 [0.602–0.911]
*Distance to first cue*	0.460 [0.259–0.697]	0.556 [0.271–0.788]
*Distance to last cue*	0.445 [0.244–0.685]	0.409 [0.109–0.695]
**MedDrive score**	0.852^†^ [0.740–0.934]	0.911 [0.814–0.929]

*ICC (3,1) is the intra-class correlation coefficient using 2-way mixed single measures. ICC ranges from 0 to 1; 0 showing no ability of the measurement to distinguish individuals one from another with a single measurement, and 1 revealing an optimal ability to clearly distinguish all individuals using a single measurement*.

† *Significant session effect was observed (p < 0.05)*.

Once trained, young participants required 17 min (ranging from 15 to 29 min) to perform all four MedDrive tasks, and 2 min (ranging from 1′ 38″–2′ 38″) to perform the movement detection task (Task 3).

## Study 4—study on MedDrive's psychophysical properties

### Methods for study 4

#### Study population

Participants in the spring 2013 refresher course (for further details see Section Study Population) were also invited to volunteer for two 2½-h sessions during which a series of additional tests were undertaken in a dedicated lab. To be included, volunteers had to detain a non-restricted valid Swiss driver's license, be capable of driving without assistance, and be exempt of a know diagnosis of dementia, Parkinson's disease, sequels of brain injury (e.g., trauma, stroke), or other important affections known to affect cognition. Participants' average age was 75.6 years (*SD* = 5.0, range 69.3–90.6), 68.1% were males, and 36.2% had followed less than 12 years of scholarship.

#### Psychophysical tests

A researcher, blind to the results from MedDrive, tested visual acuity [Landolt C, FrACT version 3.7l (Bach, 1996)]; contrast sensitivity (Gabor patch); vernier acuity, visual backward masking (Herzog and Koch, 2001; Roinishvili et al., 2011); motion direction sensitivity, orientation sensitivity, biological motion, and visual search (16 objects); the Simon effect, auditory volume sensitivity, auditory pitch sensitivity, simple response time, and executive functions (Wisconsin Card Sorting Test); verbal fluency, and working memory (digital forward and backward task). Details on these tests are provided as a supplementary document (Presentation 2, Description of psychophysical tests).

#### Statistical analysis and ethical standards

After verifying that observed values were normally distributed and that the nature of each association was linear, we used linear regression to compute the magnitude of the association between the psychometrical outcomes and outcomes from MedDrive. Results are reported as the proportion of explained variance between variables. This corresponds to the squared Person's correlation coefficient. Significance level was set for *p* < 0.05 without Bonferroni correction as measures were believed to be correlated one to another. All patients gave their written informed consent to participate. Ethical approval was obtained from official state Biomedical Ethics Committees under reference number CE384/2011.

### Results from study 4

**Figure 5:**
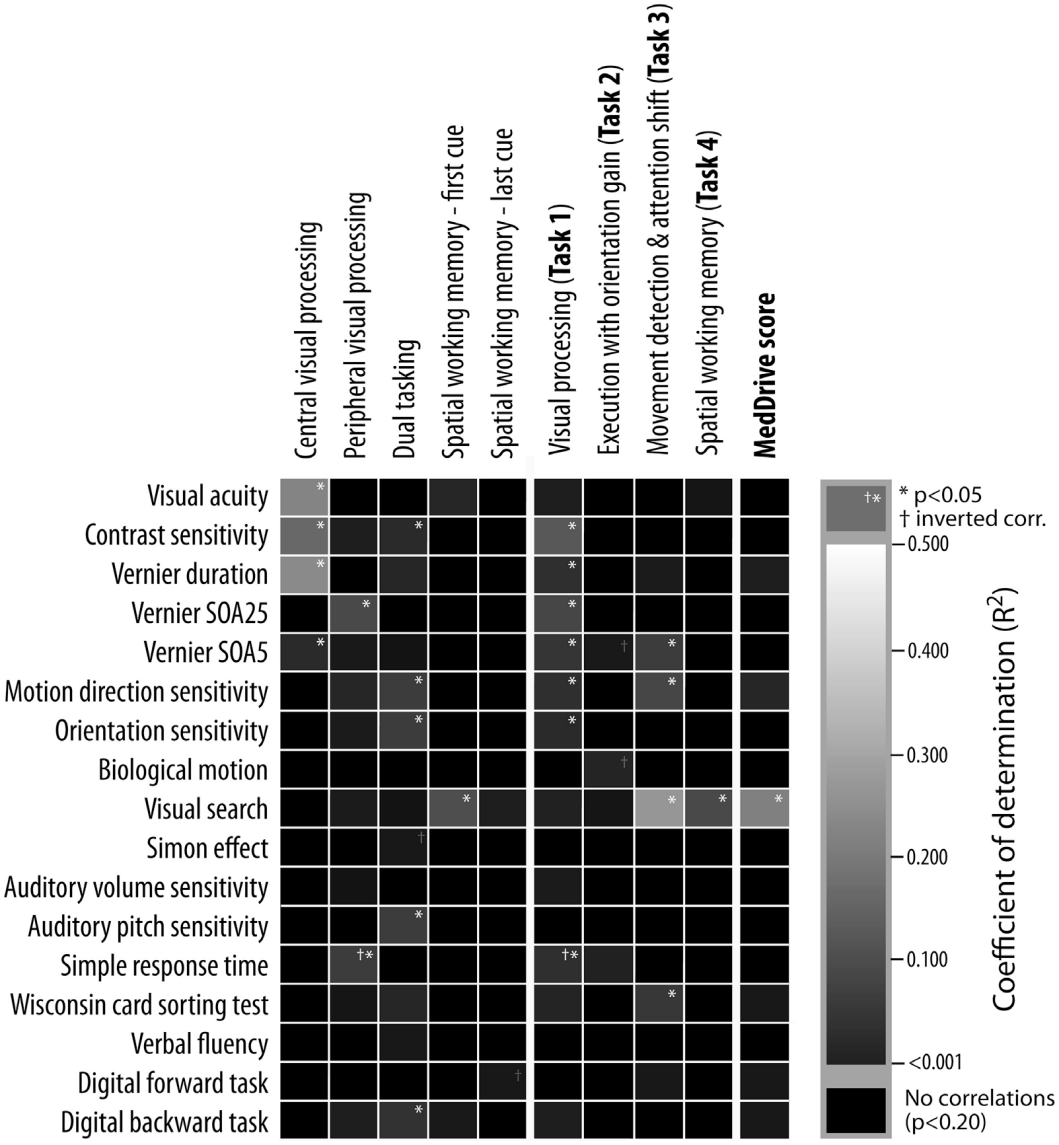
**Underlying psychophysical properties of MedDrive outputs**. Columns represent outputs from MedDrive, whereas rows represent results from psychophysical and neuropsychological tests. The lighter the square, the stronger is the correlation between the two. White stars correspond to a significant level of *p* < 0.05 unadjusted for multiple testing. Visual processing score was significantly associated to all visual processing measures except for biological motion detection. The output from the movement detection task (Task 3) was associated to visual processing, visual search, and to mental flexibility (WCST). Interestingly enough, when all these measures were combined in a single score (MedDrive score) this score was then highly correlated to visual search. This leads us to believe that the MedDrive score is a good indicator of a person's ability to detect and interpret peripheral visual stimuli. SOA, stimuli onset asynchrony; *R*^2^, coefficient of determination; WCST, Wisconsin card-sorting test.

The 47 older drivers who participated in the psychophysical tests showed similar traits to participants from Study 2. Figure 5 reports coefficients of determination (*R*^2^) between MedDrive components and other psychophysical measurements.

Together, contrast sensitivity (*p* = 0.016), visual masking (SOA25; *p* = 0.034), and simple response times (*p* = 0.006) explained 35.6% of the variations observed during the visual recognition task (MedDrive, Task 1). Furthermore, the inversed correlation of visual processing speeds with simple response times suggests that older drivers who tend to be faster at processing stimuli in the central visual field might be those that tend to perform more poorly in tasks during which the target location is unknown.

The spatial working memory task was correlated to visual search (*R*^2^ = 0.143, *p* = 0.009) but not to the digital backward task (*R*^2^ = 0.023, *p* = 0.313). This is probably due to the nature of the task, which requires drivers to rely on their peripheral vision to spot newly arriving cues and remember where they were. Finally, visual search was important enough to come out as the only significant correlate to the overall MedDrive score (*R*^2^ = 0.256, *p* = 0.001).

## Study 5—responsiveness to alcohol and ability to model driving performance on the driving simulator

### Methods for study 5

We ran a four-way, dose-response, crossover, double blind, placebo-controlled, randomized study to test the effects of different blood alcohol concentrations (BAC) on MedDrive measurements. From February to April 2013, 20 healthy young adults volunteered to participate and were included in the study. They were aged 23–40 years, 11 were women, and eight had had 18 or more years of scholarship. Fifteen participants reported alcohol misuse (AUDIT-C >4 for men, >3 for women), and one participant was a very occasional consumer of alcohol (AUDIT-C score = 1). Using Widmark's formula, participants were given cranberry juice with different doses of ethanol to bring their BACs to 0, 0.5, 0.65, and 0.8 g/L. Before recruitment began, each participant identification number was allocated to a random sequence order of BAC levels that were defined using random digits from a table. Prior to each session, participants were asked not to have eaten during the four previous hours and not to have consumed any psychotropic substance during the 24 previous hours. Participants were blinded to the presence of ethanol by inhaling ethanol vapor just before drinking. The researcher supervising the measurements was blinded to the allocation. BAC was maintained and monitored during the entire experiment by a second researcher using a Breathalyzer and administrating drinks every 15 min. Participants were previously trained in using MedDrive by performing all tests five times. For further details we have made the study protocol available (Presentation 3, Protocol for study 5).

#### Driving tasks and measurements from the driving simulator

To measure driving performance, we used a driving simulator (StSoftware PvW-2010). The simulator is a mock-up of a car with normal controls (car seat with seat belt, steering wheel, pedals, turn signals, horn, mirrors). Three screens provided a 2-D view of the environment on 200° centered on the driver's head. We then used three scenarios modified from those used in a previous study (Veldstra et al., 2012): a road-tracking task, a car-following task, and a car-following task with dual tasking. During the road-tracking task, participants were requested to drive on a straight road at 120 km/h during 7 min. The lateral position standard deviation was then measured during the entire task. During the car-following task, they were requested to follow a lead car on a road with slight curves. This car has a random sinusoidal speed change ranging from 90 to 110 km/h. A third task consisted of simultaneously following the lead car and responding to peripheral stimuli. A red cross would either appear on the left or the right screen at a 90° visual angle. Participants were asked to respond as rapidly as possible by activating the turn signal in the direction of the stimuli.

#### Statistical analysis and ethical standards

We powered the study to detect an effect size of 1.25 using an ANOVA with four levels of factor. With a power set at 0.8 and a significance level at 0.05, this required including 16 participants. Anticipating difficulties in reaching targeted BAC levels, we decided to include 20 participants.

We used random-effect GLR regression to compare effects of different BACs on MedDrive measurements. Using log likelihood ratio tests, ltests, linearity of effects was tested by comparing regression models with dichotomized BAC groups to sBACs entered as a continuous variable. Significance level was set at *p* < 0.05. The entire analysis was pre-programmed on STATA 12 and then executed.

All patients gave their written informed consent to participate. Ethical approval was obtained from official state Biomedical Ethics Committees under reference number CER12/277. This study was also registered as a clinical trial before the study began (ClinicalTrials.gov NCT01781273). All steps in developing MedDrive were conducted in accordance with the principles of the declaration of Helsinki (6th revision, Seoul).

### Results from study 5

#### Control of blood alcohol concentrations and the effects of alcohol

Our first challenge was to make sure we were controlling blood alcohol concentrations (BACs) correctly. We initially used two calibrated Breathalyzers simultaneously to verify the exactitude of our BAC measurement. On average, participants had to be given 69% more ethanol than that which would have been required using the Widmark formula (Posey and Mozayani, 2007). Each individual's coefficient to adjust to Widmark's estimation remained stable throughout the study and ranged from 1.4 to 2.0 depending on participants. Using individuals' personalized coefficients made it possible to reach, with precision, the expected BAC; we obtained an average BAC of 0.78 g/L for the 0.8 g/L group, 0.64 g/L for the 0.65 g/L group, and 0.49 g/L for the 0.5 g/L group. The observed standard deviation from the expected BAC was 0.04 g/L. When under placebo conditions (BAC = 0 g/L), none of the participants had traces of blood alcohol or had reported any alcohol consumption within the previous 48 h that could have led them to have a BAC ≥ 0.01 g/L. Furthermore, measuring BAC every 15 min made it possible to confirm BAC was adequately maintained throughout the hour required to perform all measurements. Therefore, our only limitation in controlling for effects of alcohol was that participants might not have been completely blinded to their allocation as their guess on which BAC group they were in was above chance (Kappa = 0.383, CI95% 0.255–0.511). Their guess was mainly orientated by residual taste (63.7%), perceived effect on their performance during tasks (62.5%), and other side effects of alcohol (57.5%). We therefore cannot exclude that participants might have unnoticeably slightly changed their behavior by voluntarily performing more poorly under placebo conditions (BAC = 0 g/L).

#### Responsiveness to alcohol

**Figure 6:**
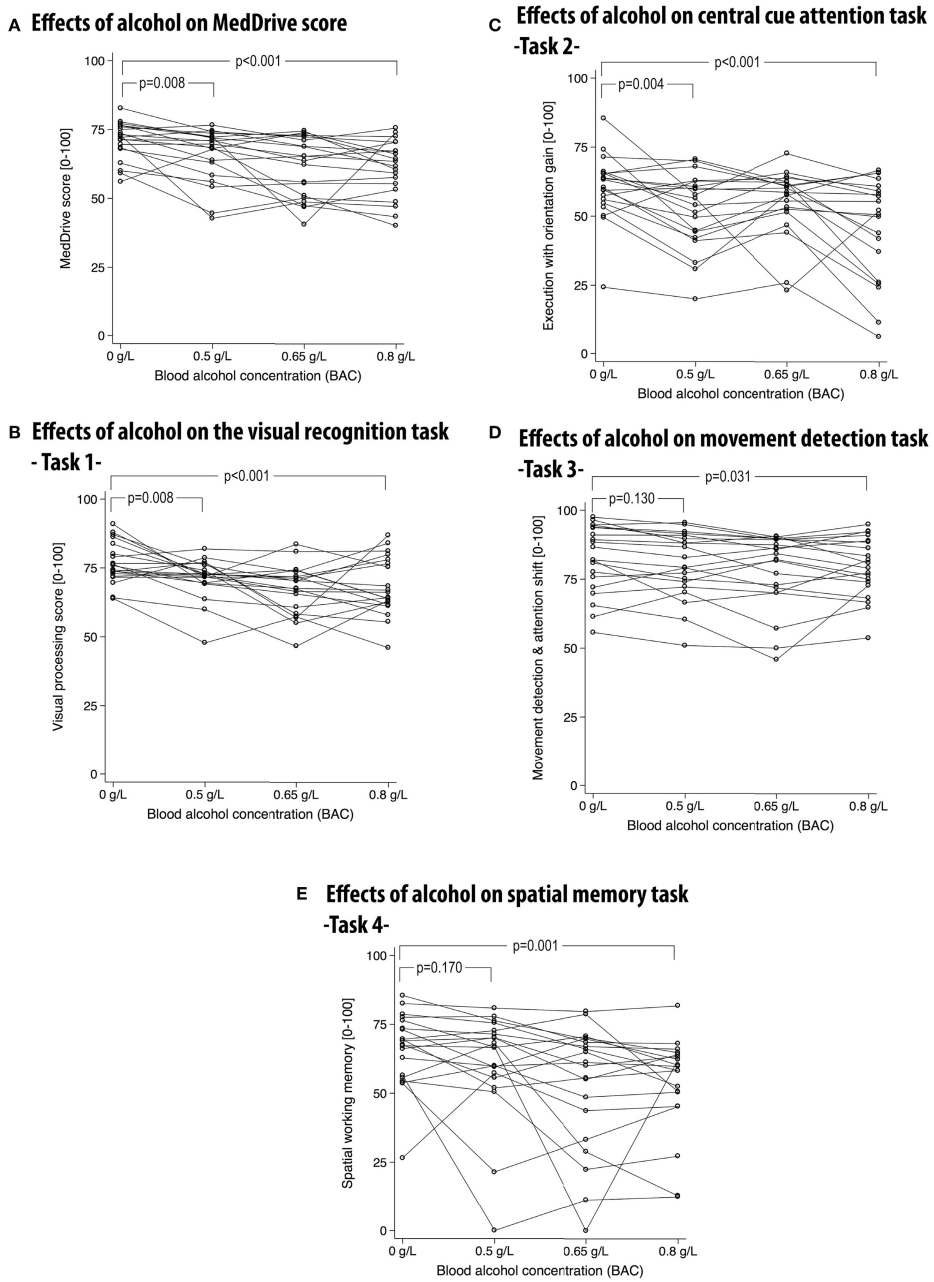
**Effects of alcohol on the overall MedDrive score **(A)** and for each of its composing subtasks **(B–E)****. MedDrive's responsiveness to different blood alcohol concentrations. Lines represent changes of performance across different blood alcohol concentrations (BACs). Lower scores represent worse performance. *P*-values correspond to Wald's test of significance comparing differences in performance between the control condition (BAC = 0 g/L) and other BACs. BAC, blood alcohol concentration.

Alcohol had significant dose-dependent effects on the MedDrive score (*R*^2^ = 0.366, *p* < 0.001) and compared to placebo this effect was observable for concentrations as low as 0.5 g/L (ΔMedD = −5.3 points, *p* = 0.008). The effect of alcohol on the overall MedDrive score was not attributable to outliers. Out of 20 participants, 15 saw their score decrease with a BAC at 0.5 g/L (Figure 6A). On the other hand, the spatial memory task (Task 4) was more vulnerable to important changes in a minority of participants (Figure 6E). This was probably related to some participants having inverted responses when spatially placing the first and last cue when under the influence of alcohol. The movement detection task (Task 3) was less influenced by alcohol than other outcomes making this MedDrive's most stable outcome (Figure 6D). At low doses, alcohol had significant effects on visual processing (Task 1) and execution with orientation gain (Task 2) whereas it was not significant for the other two MedDrive tasks (Figures 6B,C). All MedDrive outcomes were significantly influenced by alcohol at concentrations of 0.8 g/L compared to placebo.

#### Ability to model driving performance changes on the simulator due to alcohol

**Figure 7:**
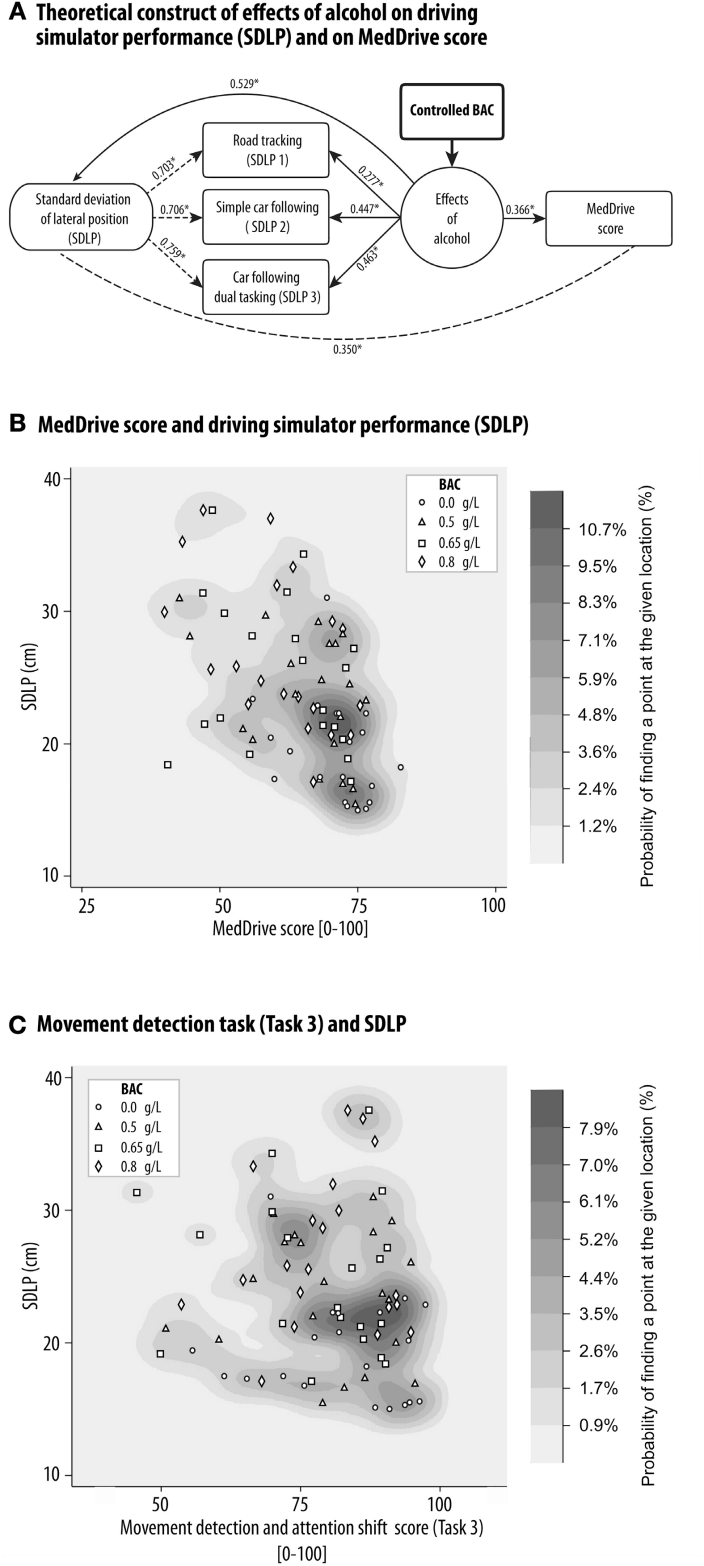
**MedDrive's ability to model the effects of alcohol on driving simulator performance**. Blood alcohol was controlled at **four** different concentrations in 20 young healthy drivers, and the effects of alcohol on the standard deviation of the lateral position during **three** driving simulator tasks was measured. **(A)** Schematic representation of causal links between alcohol, driving simulator performance, and MedDrive score. Numbers represent the ability of each link to model the effects of alcohol at an individual's level (*R*^2^). ^*^p < 0.001. Dashed lines mean that we do not assume a causal relationship between measures. Density distribution plot of the correlation between SDLP and MedDrive score **(B)** or Movement detection task **(C)**. The oblique orientation of the distribution associated to the gradient of BACs illustrates the shared causal link of effects of alcohol on driving performance and on MedDrive score. **(C)** Density distribution plot of the correlation between SDLP and MedDrive's Task 3 score. Even if the oblique gradient is less obvious than for the MedDrive score, the movement detection task alone was still able to model the effects of alcohol on SDLP (*R*^2^ = 0.178, *p* = 0.027). BAC, blood alcohol concentration; SDLP, standard deviation of lateral position; *R*^2^, coefficient of determination.

The side effects of alcohol are very similar to those of car- or simulator sickness. Our main difficulty was therefore to distinguish the direct effects of alcohol on simulator driving performance from those indirectly caused by simulator sickness. This was important as we observed alcohol to significantly increase simulator sickness (*R*^2^ = 0.152, *p* > 0.001). With a BAC of 0.8 g/L, participants saw their SSQ score increase by an average of 5 points (CI95% 2.5–7.4). Our summary measurements from the simulator tasks (mean SDLP) however reacted to BAC independently of simulator sickness (ß = +8.7 cm per g/L OH, *p* < 0.001). Effects of alcohol explained 52.9% of observed differences between sessions of average SDLP over the three driving simulator tasks. The theoretical model linking alcohol consumption, measurement of its effects on driving performance, and its relation to MedDrive is illustrated in Figure 7A. MedDrive was capable of modeling 36.6% (*p* < 0.001) of the effects of alcohol on driving simulator performance (Figure 7B). MedDrive's ability to model the effects of alcohol on driving performance was also observed for each of its tasks. The movement detection task (Task 3) modeled 17.8% of the effects of alcohol on SDLP (Figure 7C, *p* = 0.027). For the visual processing score (Task 1; *R*^2^ = 0.120, *p* = 0.014), we noted however that the peripheral visual processing score (*R*^2^ = 0.037) and the dual tasking score (*R*^2^ = 0.038) were less correlated to SDLP than was the central processing score (*R*^2^ = 0.123).

### Adverse events and eventual side effects

During the experimental study with alcohol, undesired, reported adverse events were mostly attributed to alcohol absorption. During the resting period, two participants felt nauseous enough to vomit (BAC = 0.8 g/L), and seven complained of a slight headache the day after having received alcohol. Participants also complained of benign temporary lack of concentration (*n* = 5, occurrence = 5), sleepiness (*n* = 9, occurrence = 13), or eye fatigue (*n* = 7, occurrence = 8). The implication of MedDrive in these symptoms cannot totally be excluded. During the validation study, four participants from the 184 volunteers stopped the tests complaining of eye fatigue or lack of ability to concentrate. One did so before the tasks even began. Given these drivers manifested difficulties during other tests, it is difficult to say if these symptoms were truly caused by MedDrive or not.

## Discussion

### Overview of results

#### MedDrive's validity

Results from confirmatory structural equation modeling showed the construct of the instrument to be consistent. We also observed very important differences of value between young and older adults suggesting this measure to be age-related. However, given the central visual processing task contributed the least to the overall appreciation, we cannot exclude that the measured trait is related to increased difficulties in controlling attention shift without eye movement. In other words, older drivers tend to mainly have reduced processing speed when discriminating and localizing eccentric objects (Gruber et al., 2013). This could also explain MedDrive's composite score's important association to visual search that would also be affected by increased difficulties in detecting eccentric images, and planning eye movements.

Given both eccentric object detection and visual search are important for drivers to correctly monitor motor vehicle control, it is not surprising that our composite score is associated to driving behavior. However, we have to note that MedDrive remains limited in distinguishing clearly those fit to drive from those unfit to drive even if our results suggest MedDrive to perform better than the UFOV in predicting driving behavior and performed better than most existing tests (Mathias and Lucas, 2009). Our results therefore confirm the important limitation of these tests and the important risk (9 out of 10 positive drivers) of falsely having older drivers cease driving when solely relying on neuropsychological tests.

#### MedDrive's reliability

Given we gave ourselves the constraint of choosing a homogenous and healthy study population, we did not expect participants to perform very differently one from another, thereby making it difficult to distinguish variability between measurements from the same subjects from variability of measurements between subjects. In this context, an overall ICC(3,1) of 0.852 from the first measurement onward is exceptional and reveals MedDrive's ability to also identify differences in performance for healthy young people. The learning effect through the first three sessions we observed suggests that, for experimental research purposes, study participants should run MedDrive tasks at least three times before starting measurement under controlled conditions. Training can nevertheless take place at home on their personal computer. For observational studies during which less time is available for testing, the movement detection task (Task 3) could be of interest as it was shown to be reliable from the first measurement onward (ICC[3,1] = 0.879). These results however need to be confirmed for older adults.

#### Responsiveness to alcohol

MedDrive responded above expectations to alcohol consumption in young adults, making it possible to detect effects with blood alcohol concentrations as low as 0.5 g/L. We were also able to show that the effect of alcohol on the MedDrive score was related to changes in driving performances on the simulator. But most interestingly, if we compare MedDrive's score from young drivers with 0.8 g/L BAC, to the same score from very good performing older driver in their normal state (Figure 4A vs. Figure 6A), we observe that over half of older good drivers did worst on the MedDrive score than any of the young impaired drivers. This suggests that effects on MedDrive score can lead to different behavioral consequences depending of the context in which these changes occur. Abrupt changes of small magnitude have important effects on behavior whereas progressive slow age-related changes of higher magnitude can have little or no effect on behavior.

### Aging and processing speed

Our model for measuring cerebral decline ended up including all the measurements that had previously been identified as been related to normal aging; those linked to attention shift, dual tasking, managing distractors, and noticing changes (Groth and Allen, 2000). Results from our psychophysical study suggest that increased age-related demands in cerebral processing could explain the increased difficulties in visual search tasks. When modeling age-related changes at a behavioral level, Earles and Salthouse came up with a very similar construct to our own (Earles and Salthouse, 1995). They also showed the importance of altered processing speed which led to the processing-speed theory that assumes observed age differences in task performances to be due to the reduction in speed of underlying “cognitive operations” (Salthouse, 1996). Reduced processing speed accounts for most other age-related changes in working memory, episodic memory, reasoning, and spatial ability (Verhaeghen and Salthouse, 1997). The fact that there was a temporal constraint within our spatial working memory task could explain why it was so closely related to other tasks even if it did not directly measure processing time. Previous studies have shown that age-related working memory deficits are at least partially due to increased temporal needs for encoding (Lewandowsky and Oberauer, 2009) or maintenance (Barrouillet et al., 2011). The same temporal constraint could also enhance an age-related impaired updating process affecting participants' abilities to locate the last cue in our task (Stormer et al., 2012). In our study, we also noticed that once the spatial information had been encoded, its maintenance was not affected by normal aging. In other words, we were able to rule-out that normal aging was associated to important temporal working memory decay.

Our observations also support previous observations that in the absence of temporal constraints, spatial orientation is maintained in normal aging. Similarly to our observations, age-related increase in response time during the attention network test have been shown not to be due to differences in controlled conditions related to alerting or orientation (Weaver et al., 2009; Gamboz et al., 2010). This is probably due to the fact that spatial localization is preserved with aging and that the informative cue on the location of a target is given prior to display. Therefore, the attention shift in the attention neural network task is only affected by age if the stimulus onset asynchrony between the informative cue and the target cue is short enough for the older adult not to be able to process it (Nissen and Corkin, 1985). In other words, spatial difficulties are only related to temporal constraints limiting the ability to correctly identify the target location.

The single dimension of MedDrive's composite score could therefore be related to a common trait affecting performances in all tasks—reduced processing speed, loss of complexity, and dedifferentiation (Sleimen-Malkoun et al., 2014). Given that processing speed is nevertheless affected differently depending on engaged cognitive functions, there are reasons to believe that age mainly affects the way sensory processing is modulated by higher order processes (Chmielewski et al., 2014). This includes frontal cortical functions that regulate voluntary orienting without eye movement and control attention shift (Kelley et al., 2008). The importance of these functions within our tasks might explain why our composite score was highly associated to visual search but not to contrast threshold, orientation threshold, or stimulus-onset asynchrony. During a given task, the effects of age are believed to not only reduce transmission of the signal at a neural level within one or more neural networks, but also to reduce synchronicity between networks that process the signal (Grady et al., 2006) and to recruit alternative accessory compensation networks that extends the processing time. Examples of such changes are the observed differences in the small-world architecture in the functional connectivity of younger adults compared to older adults (Goh, 2011; Antonenko and Floel, 2014) and activation of additional neural networks to compensate for the loss of differentiation (Goh and Park, 2009; Burianova et al., 2013; Antonenko and Floel, 2014; Geerligs et al., 2014). These changes reduce capacities to use similar networks within a short time frame at the response-selection stage (Allen et al., 1998). Therefore, the time span is increased for response retrieval if a network is solicited by another prioritized processing demand before it has ended processing the first demand (Anderson et al., 1998). This bottleneck phenomenon seems to be task dependent as more demanding tasks show increased age-related differences (Vaportzis et al., 2013). Therefore, manifestation of age-related cerebral decline at a behavioral level might only appear when the task load is important enough.

### Links to behavior in a natural environment

Scanning the environment for potentially threatening situations, performing more than one action at a time, managing distractors, noticing changes, and rapidly memorizing spatial locations of objects are essential for correctly monitoring automated, crystallized execution functions operating while driving a motor vehicle (Macadam, 2003; Lees et al., 2010). Therefore, reduced processing speed induces noticeable changes in driving behavior (Classen et al., 2010) that increase the risk of been involved in specific kinds of accidents (De Raedt and Ponjaert-Kristoffersen, 2001). It is therefore not surprising that the MedDrive score and its components were associated to on-road performance and driving habit changes. What was expected, but is more surprising, is the low magnitude of these associations. The difficulties in transposing measurements of cognitive functions to driving performance are however already known (Mathias and Lucas, 2009). MedDrive's ability to detect driving difficulties is better than many existing tests including those that were tested on drivers with cognitive disorders (Reger et al., 2004; Silva et al., 2009). What is even more surprising is that even if MedDrive is capable of detecting the effects of low blood alcohol concentrations (BAC = 0.5 g/L) in young drivers, the magnitude of effects under these conditions are much lower than the observed changes that will occur with aging. In other words, if behavioral changes due to alcohol were of the same nature as those observed with aging, 70 year-old drivers would permanently feel like a young driver with more than 1.5 g/L BAC. This leads us to believe that the effects of reduced processing speed on behavior largely depend on the time frame during which it appears; 20 min for alcohol vs. 50 years for normal aging. Despite the massive reduction of performance in processing speed, older adults are capable of driving due to compensation mechanisms that see their brains adapt to their conditions and maintain a constant level of risk (Wilde, 1982). The posterior-anterior shift observed with aging reflects this compensatory mechanism that maintains cognitive performance (Davis et al., 2008). Older adults also display more activity in the dorsal prefrontal cortex and parietal cortex compared to younger adults, suggesting a higher top-down activity of the dorsal attention network (Corbetta et al., 2008; Vallesi et al., 2011) In tasks requiring attention in both right and left visual fields, older adults show increased bilateral activation of the prefrontal, compared to younger adults (Davis et al., 2012). Modified cortical activity, associated with improved performance, supports the idea that increased activity in the prefrontal cortex might be engaged as a compensation mechanism. The benefits of these compensation mechanisms are therefore task-specific and response-specific with slower responses but improved accuracy (Grady, 2008). Compensatory mechanisms are therefore put in place by recruiting additional brain regions if these can functionally contribute to improving or maintaining performance in daily tasks (De Chastelaine et al., 2011). Brain plasticity can therefore put up a cognitive reserve that will be effective in compensating for the effects of age on processing speed as long as the demand does not exceed the reserve (Steffener and Stern, 2012). For driving, most adults reach this limit after the age of 85 years (Dellinger et al., 2001). MedDrive and other instruments measuring processing speed therefore probably only transpose to observable natural behavioral changes when older adults have not had the opportunity to regularly practice the given task or when cerebral decline is sufficiently advanced for compensation mechanisms not to be efficient anymore. The actual state of knowledge nevertheless leaves room to debate whether reduced performance in cerebral processing speed is the cause or the consequence of neural network reorganization in older adults (Park et al., 2012; Voss et al., 2013).

### Limitations

Even if our instrument has tried to optimize the measurement of cerebral decline in processing speed, it has some limitations. The association to age has been measured using cross-sectional data and remains subject to bias. We have also shown that individuals display different sensitivities to different features. Therefore, the measurement related to the central visual processing task lacks precision and validity as it falsely assumes that underlying psychometric functions are similar. It will be necessary to improve the task by adding a step prior to the stepwise procedure. This consists of modifying parameters (i.e., shape and contrast) and thereby defining individual thresholds for detecting changes in traits without temporal constraints. This will also make it possible to compensate for optical defects.

Contrarily to recommendations (Berghaus et al., 1997), risk avoidance and sustained attention were not included in our battery of tests. Risk avoidance is probably a consequence of brain maturation and is close to impossible to measure in an artificial environment. Sustained attention is most likely not affected by normal aging as it has even been shown to improve under specific conditions (Staub et al., 2013). As tasks related to sustained attention are time consuming, we therefore decided against including a specific task that was very likely to not contribute to our final model. The choice of our battery of tests was subjective and relied on experts' opinion and existing knowledge on effects of age on cognition from previous studies. We therefore cannot exclude that we failed to integrate a parameter that could yet improve the measurement of visual processing related cerebral decline.

Our instrument has revealed itself to be very reliable. This is partially due to the integration of the learning function within the instrument. In young adults, three sessions were sufficient to overcome the learning effect. Nevertheless, these results might not be transposable to older adults for whom the learning effect might require more sessions (Bherer et al., 2008; Strobach et al., 2012).

We believe our instrument provides an overall indication on processing speed related to visual processing and cerebral decline. We however cannot rule out that another unknown mechanism could provide an alternative explanation. We believe that accessory networks are recruited in older adults. In other words, we could be measuring a surrogate of dedifferentiation. This however needs to be confirmed by imaging studies. Our instrument is therefore adapted for experimental designs that aim to test the effects of interventions on cognitive functions but not for providing explanations regarding underlying neural processes.

### Consequences for currently held views

This study supports the idea that normal aging is accompanied by brain changes that affect processing speed. These changes nevertheless do not necessarily affect driving behavior as they are compensated for. In consequence, older adults might react completely differently than would younger adults to other constraints that require additional compensation. Nevertheless, most studies investigating the effects of drugs on driving performance rely on young adults only, even if they are also dedicated to older adults. We might therefore be underestimating the adverse side effects of drugs on older adults. For the sake of credibility, pharmaceutical studies of driving risk should also target older adults or at least clearly state that effects on driving performance are unknown for this population.

A second important implication is that given that the effects of changes in cerebral processing on behavior are largely dependent of the context (Harel et al., 2014), in normal aging it is difficult to transpose results from neuropsychological tests to behaviors in a natural environment. Clinical use of such tests therefore only makes sense if interpretations are contextualized. The time frame of the onset of the impairment and opportunities for older adults to have practiced the behavior under evaluation need to be taken into account before assuming that adults have lost their competency to perform a given task. Our results reveal that in no way should MedDrive alone lead to banning older drivers from driving without disposing of complementary information on their driving behavior. Concerning this point, there is an important gap between current clinical guidelines and actual knowledge when assessing fitness to drive (Ama/Nhtsa, 2003; Messinger-Rapport, 2003; Murden and Unroe, 2005; Odenheimer, 2006; Sherman, 2006; Iverson et al., 2010; Bula et al., 2011; Mosimann et al., 2012; Lin et al., 2013b). Clinical guidelines are based on observations made in the context of pathological conditions (e.g., stroke, Alzheimer disease, Parkinson's disease) and have been transposed to normal aging without warning clinicians that interpretations of neuropsychological tests differ for this population and need to be contextualized.

This leads to the last point. Whether age-related changes reflect the consequences of brain maturation and a stabilization of neural networks or a natural decay corresponding to our individual “mindspan” remains debatable. We must nevertheless keep in mind that cerebral decline can be considered to be a normal processes and is often accompanied by improved cerebral processing for other functions such as emotional control (Voss et al., 2013). Cerebral decline is therefore not necessarily a disorder and should also be studied without it being regarded as a pathological condition (Graham and Stephenson, 1992). This is indispensable for preventing elderly people from being stigmatized for their cognitive problems.

### Future directions for research

One of the most important problems we still have to solve is to understand the inter-level constraints (Kistler, 2009) that link age-related neural reorganization to active lifestyle and maintained cognitive functions. In other words, how do these two phenomena, which occur at different levels of organization, interact? Investigating models that integrate neural plasticity, cognitive reserve, and driving needs seems essential in understanding and defining when driving cessation becomes necessary.

The second direction for research to take is to develop and test the effects of targeted cognitive training on behavior in natural environments. This is challenging, as training programs would need to address impaired cognitive functions that are not compensated for. The first step is therefore to develop instruments to detect these functions before developing and testing interventions that will not only improve performance on the cognitive task, but also improve behaviors that will help maintain an active lifestyle.

## Author contributions

Paul Vaucher, Bernard Favrat, and Michael Herzog applied for the grants; Paul Vaucher, Michael Herzog, and Daniela Herzig wrote the protocols; Bernard Favrat, Janet L. Veldstra, and Patrice Mangin provided input and validated the protocols; Paul Vaucher and Bernard Favrat reviewed the literature; Paul Vaucher and Isabel Cardoso collected, analyzed, and interpreted data for the qualitative study; Paul Vaucher conceived of and designed the tasks included in MedDrive under Bernard Favrat's supervision; Paul Vaucher and Isabel Cardoso collected data for the validation study with seniors and the experimental study regarding alcohol; Janet L. Veldstra programmed the scenarios for the driving simulator; Daniela Herzig collected data for the psychophysics study; Paul Vaucher and Bernard Favrat planned the statistical analysis; Paul Vaucher analyzed data; all authors interpreted the results; Paul Vaucher wrote the manuscript draft; all authors have given final approval of this version of the manuscript.

### Conflict of interest statement

The reviewer, Dr. Annoni declares that, despite having collaborated with the authors, the review process was handled objectively. Bernard Favrat and Paul Vaucher have developed a computerized battery of neuropsychological tests to assess cerebral fitness, a battery entitled MedDrive. Copyrights are detained by the University of Geneva and the University Hospital of Lausanne. Bernard Favrat and Paul Vaucher have officially renounced any financial interest in the eventual commercialization of this instrument. The authors declare that the research was conducted in the absence of any commercial or financial relationships that could be construed as a potential conflict of interest.

## Supplementary material

Presentation 1 **Supplementary figures 1-4**.

Presentation 2 **Description of Psychophysical Tasks for Study 4**.

Presentation 3 **Protocol for Study 5**.

## Abbreviations

ANT: attention network test
BAC: blood alcohol concentration
CD: coefficient of determination
CFI: comparative fit index
GFI: goodness-of-fit statistics
LRtest: likelihood ratio test
R2: coefficient of determination
RMSEA: root mean squared error of approximation
SD: standard deviation
SDLP: standard deviation of lateral position to the middle of the road
T1: visual recognition tasks included in MedDrive
T2: central cue attention task included in MedDrive
T3: movement detection task included in MedDrive
T4: spatial working memory task included in MedDrive
TLI: Tucker-Lewis index
UFOV: useful field of view
UFOVmod: modified score combining results from the useful field of view using structural equation modeling
X2: Chi square test.
