# Supplementary material for: A neuropsychological instrument measuring age-related cerebral decline in older drivers: development, reliability, and validity of MedDrive
Source: Front Hum Neurosci. 2014 Oct 9;8:772. doi: 10.3389/fnhum.2014.00772 (PMC4191221; doi:10.3389/fnhum.2014.00772)
Supplement: Presentation 1 — Supplementary figures 1-4. [file Presentation1.PDF]

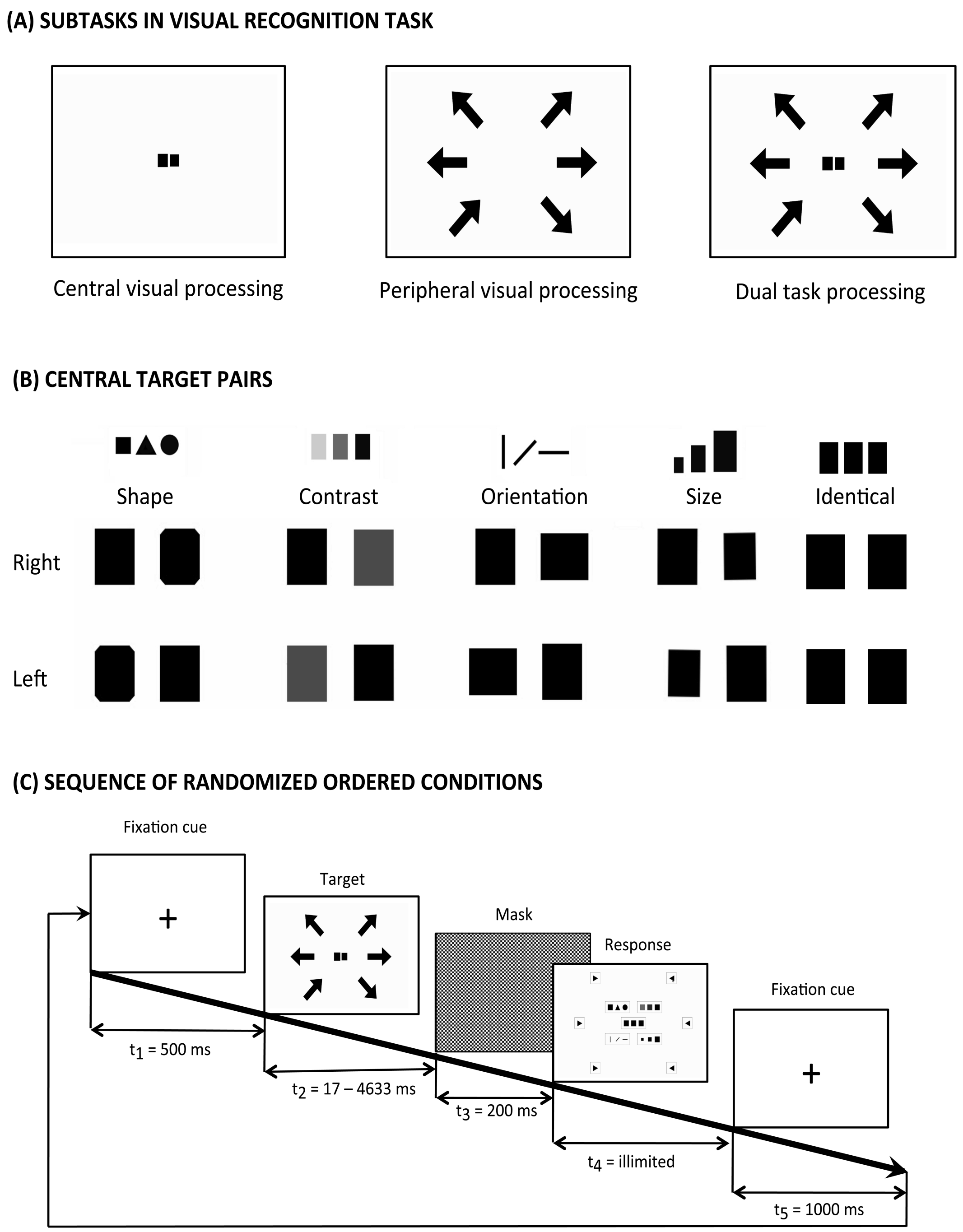

Supplementary Figure 1. Visual Recognition Task. This task includes three subtasks presented in a randomized order: central visual processing, peripheral visual processing, and dual tasking (A). The central visual processing subtasks present a pair of shapes that can either be identical or have a slightly different shape, contrast, orientation, or size (B). The peripheral visual processing subtask consists of identifying the arrow that is directed toward the center. A stepwise procedure measures the 50% threshold for correctly identifying targets independently for each subtask (C).

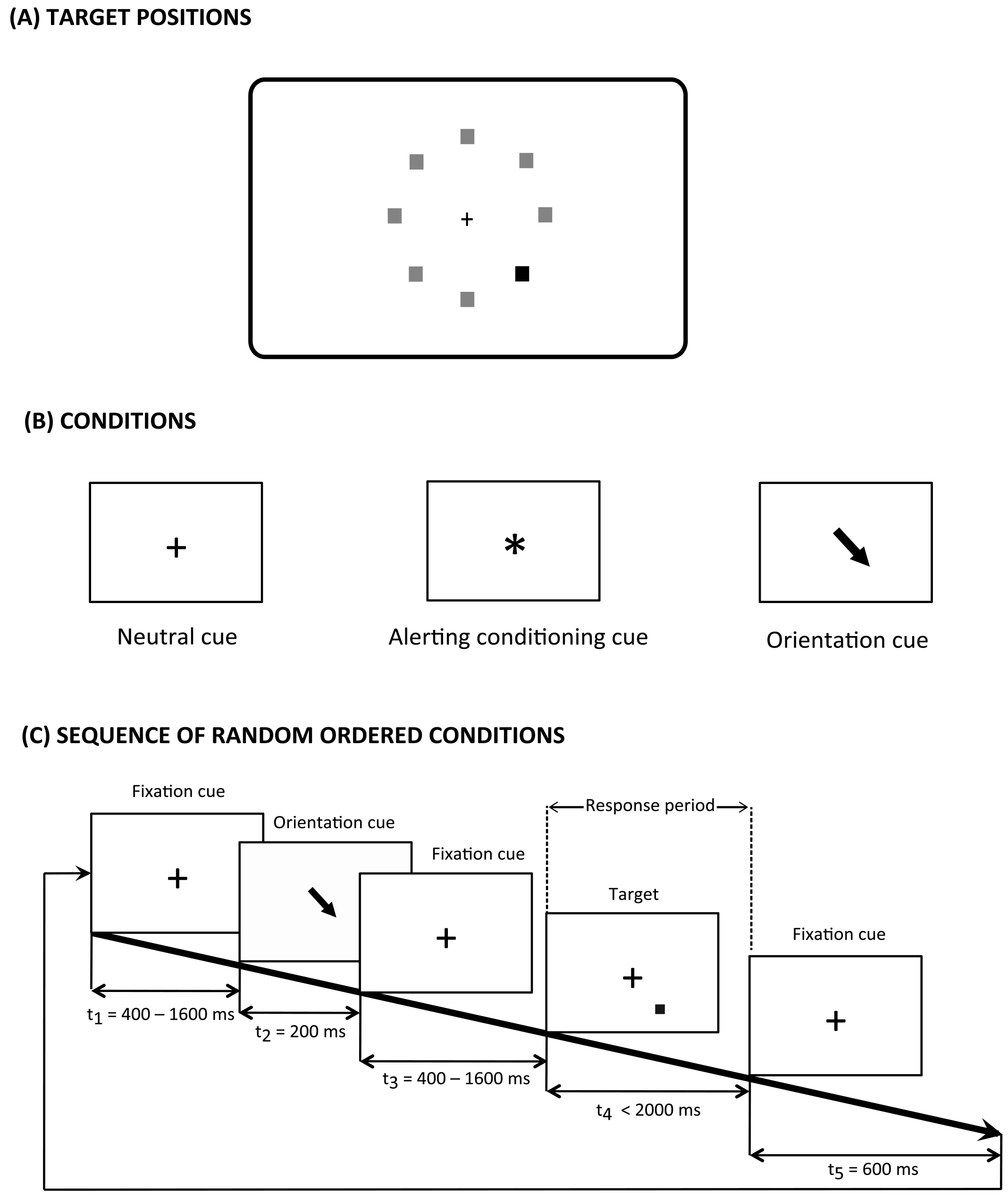

Supplementary Figure 2. Central Cue Attention Task. This task consists of pressing on the keyboard's space bar as quickly as possible after a black square appears in one of eight possible positions (A). Prior to the appearance of the target, three random-ordered conditions are possible: a neutral condition providing no other information than indicating the center of the screen, an alerting condition consisting of a star briefly appearing and warning of the imminent appearance of the target, or an orientation condition consisting of the appearance of an arrow indicating where the target will appear (B). The orientation cue appears for 200 ms at a random interval ranging from 600 to 1800 ms before the target whereas the alerting cue will consistently appear 600 ms before the target (C).

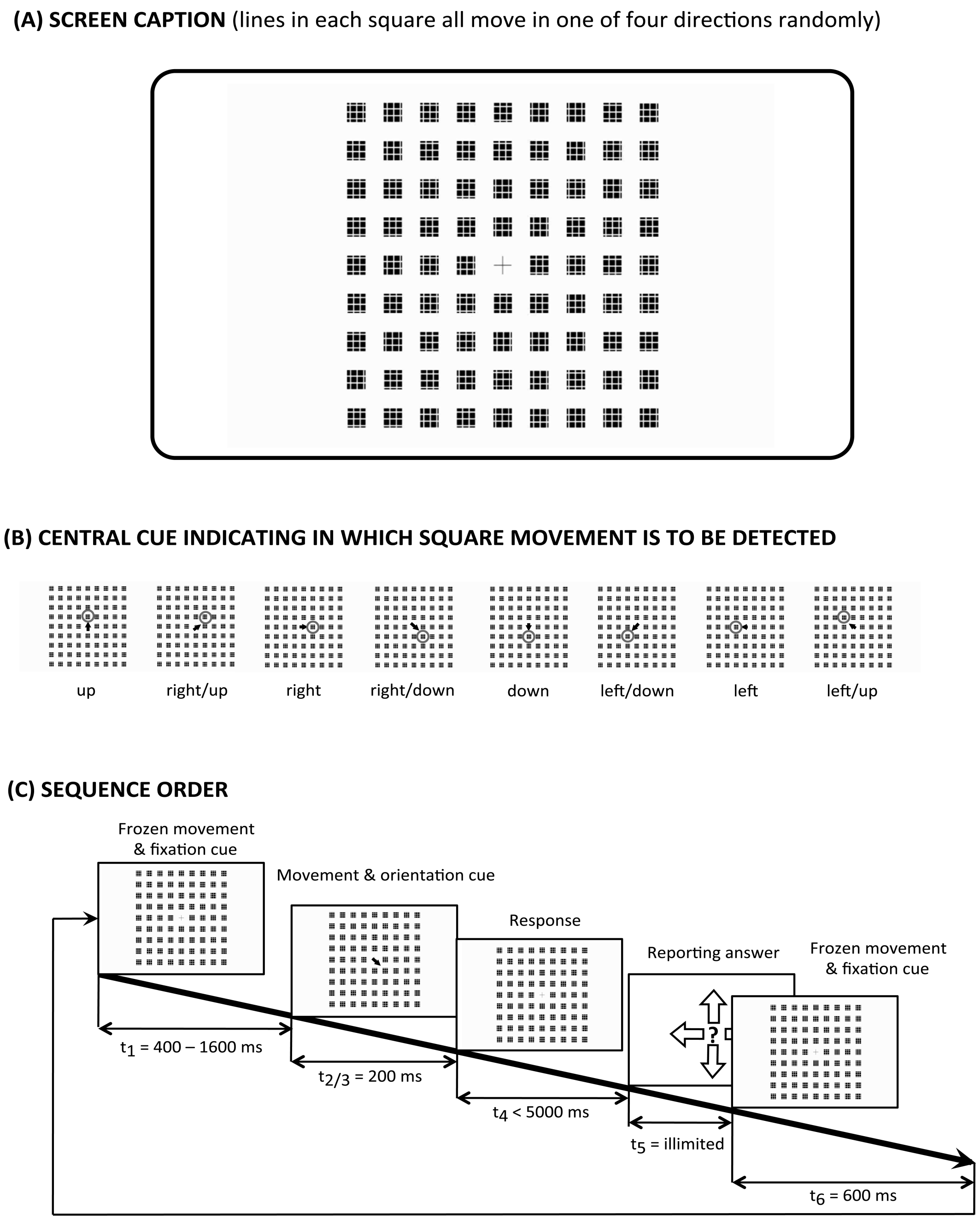

Supplementary Figure 3. Movement Detection Task. This task consists of pressing on the keyboard's space bar as soon as one is able to detect the direction of moving lines within an indicated square. Eighty squares have randomly moving lines (A). During 200 ms an orientation cue indicates which square the participant needs to attend to (B). Simultaneously, all squares start to move till the participant presses on the space bar. The participant is then given all the time they need to provide their answer. (C).

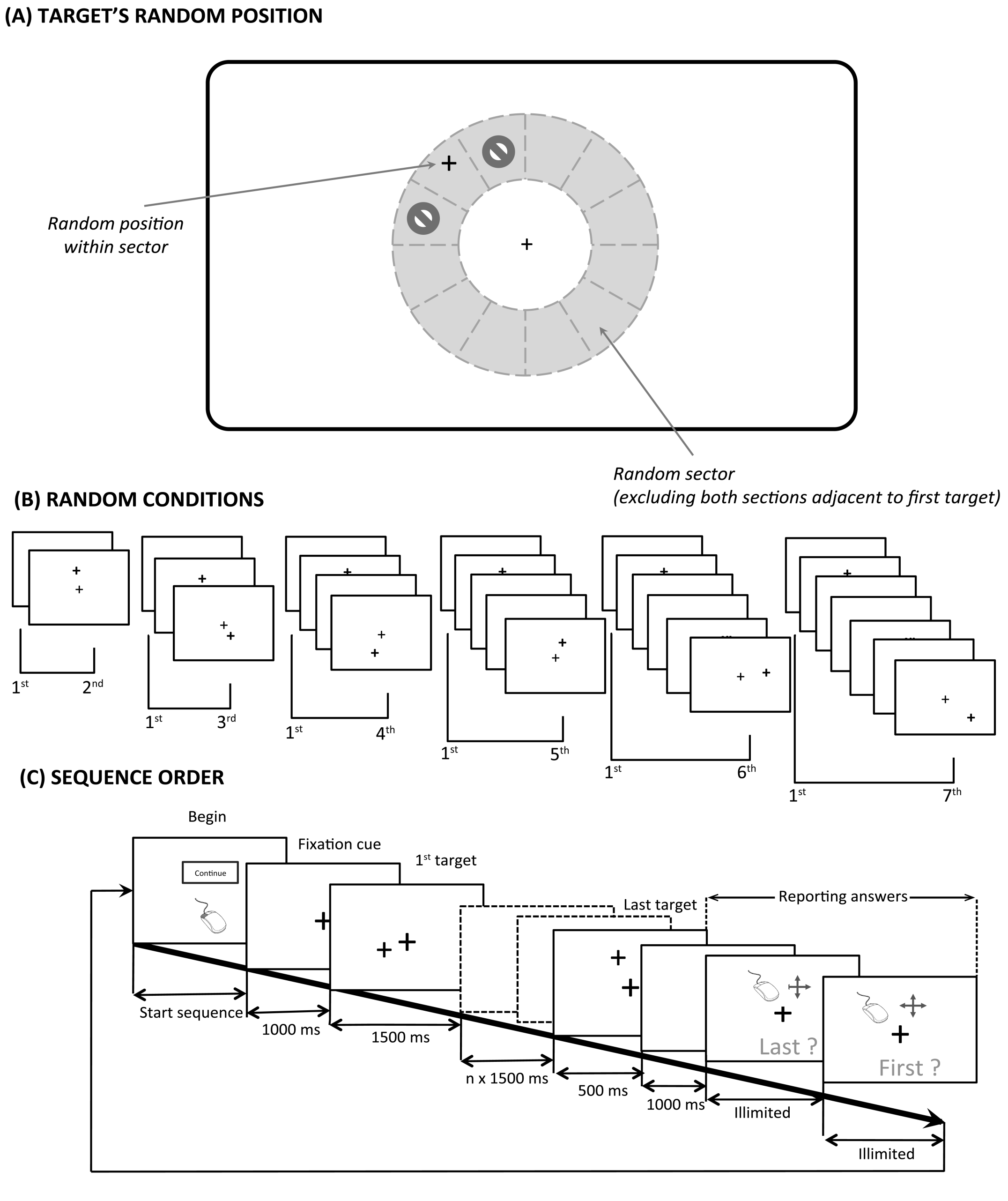

Supplementary Figure 4. Spatial Working Memory Task. This task consists of remembering the location of the first and last randomly located targets (A) within a series of crosses for which each cross after the first one could be the last (B). Once the series has ended, the participant is asked to bring the mouse pointer to the location in which they think they saw the last cross, then the location in which they think they saw the first, before starting a new series (C). The outcome is the geometrical mean of the distances between the true location and the indicated location.
