# Supplementary material for: A neuropsychological instrument measuring age-related cerebral decline in older drivers: development, reliability, and validity of MedDrive
Source: Front Hum Neurosci. 2014 Oct 9;8:772. doi: 10.3389/fnhum.2014.00772 (PMC4191221; doi:10.3389/fnhum.2014.00772)
Supplement: Presentation 3 — Protocol for Study 5. [file Presentation3.PDF]

MedDrive's responsiveness to different blood alcohol concentrations and concurrent validity against performances on a driving simulator; a phase I, randomised, double blind, placebo, dose response validation trial

- The OH-MedDrive study -

Regulatory and Funding Sponsor
Prof Patrice Mangin, MD, PhD
Centre Universitaire Romand de Médecine Légale
HUG
CMU – Bât. C
Michel-Servet 1
CH-1211 Geneva-4

Tel. +41 22 379 55 94, Fax. +41 22 379 59 02
Email: Patrice.Mangin@hcuge.ch

Principal Investigator
Dr Bernard Favrat, MD RER
Unité de Médecine et de Psychologie du Trafic
Centre Universitaire Romand de Médecine Légale
HUG / UNIGE
Michel-Servet 1
CH-1211 Geneva-4

Tel. +41 79 556 61 83, Fax. +41 22 379 59 02
Email: Bernard.Favrat@hcuge.ch

Secondary Investigator
Paul Vaucher, DiO, MSc, doctorant
Unité de Médecine et de Psychologie du Trafic
Centre Universitaire Romand de Médecine Légale
HUG / UNIGE
Michel-Servet 1
CH-1211 Geneva-4

Study product (non-invasif diagnostic instrument)
MedDrive©, a computed battery of neuropsychological tasks to assess fitness to drive

[ICH 6.1]

Signature from sponsor *[ICH 6.1]*

I hereby declare that I have read and approve this protocol and its attached documents. I certify the study was planned conformably to the Good Clinical Practice from the International Conference on Harmonisation.

All future changes to the protocol will be announced to the ethical committee. Decisions for such modifications will be taken by the trial steering committee composed of six members: Patrice Mangin, Bernard Favrat, Barbara Broers, Janet Velstra, Claire Bindschaedler, and Paul Vaucher.

Patrice Mangin		13.02.13_
**Sponsor**	**Signature**	**Date**

Professeur UNIGE, Directeur du Centre Universitaire Romand de Médecine Légale
Title

HUG / UNIGE, Michel-Servet 1, CH-1211 Genève-4
Institution and address

+41 22 379 55 94
Telephone

Signature from principle investigator

I hereby declare that I have read and approve this protocol and its attached documents. I certify the study will take place conformably to the protocol and to the Good Clinical Practice from the International Conference on Harmonisation.

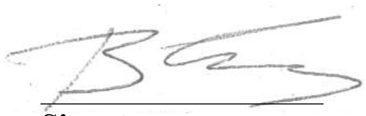

Bernard Favrat		13.02.13_
**Principle investigator**	**Signature**	**Date**

MD, Priva deucens, directeur de l'Unité de Médecine et de Psychologie du Trafic
Title

HUG / UNIGE, Michel-Servet 1, CH-1211 Genève-4
Institution and address

+41 79 556 61 83
Telephone

Signature from secondary investigator

I hereby declare that I have read and approve this protocol and its attached documents. I certify the study will take place conformably to the protocol and to the Good Clinical Practice from the International Conference on Harmonisation.

Paul Vaucher		13.02.13
**Principle investigator**	**Signature**	**Date**

DiO, MSc Clinical Trials, PhD student in Neuroscience
Title

UNIGE, Michel-Servet 1, CH-1211 Genève-4
Institution and address

+41 78 788 33 66
Telephone

Table of contents

Résumé synoptique du protocole en français 8

Study procedure 10

List of Abbrevations 11

1. Background *[ICH 6.2]* 12
1.1. Background 12
1.2. Measuring instrument under study 12
1.3. Risks related to alcohol consumption 13
1.4. Rationales 13

2. Study objectives *[ICH 6.3]* 13

3. Study design *[ICH 6.4]* 14
3.1. General design 14
3.2. Primary and secondary endpoints 14
3.3. Randomisation 14
3.4. Blinding 14
3.5. Duration of the study for participants, early withdrawal, and unblinding procedure 14
3.6. Source documents and Case Report Form 15

4. Study participants *[ICH 6.5]* 15
4.1. Inclusion criteria 15
4.2. Exclusion criteria 15
4.3. Early withdrawal 15
4.4. Subject recruitment and screening 15

5. Material under investigation (diagnostic instrument) *[ICH 6.6]* 16
5.1. MedDrive 16
5.2. Development 16
5.3. Testing effects of expectations on visual processing speed 16
5.4. Incompatible treatment or behaviour 17

6. Administration of Alcohol 18
6.1. Preparing and administrating the beverage 18
6.2. Control 18
6.3. Maintaining BAC 18
6.4. Pregnancy and breastfeeding 18

7. Study outcomes *[ICH 6.7]* 18

7.1. Simulator 18

7.2. UFOV 19

7.3. TMT 19

8. Data handling and keeping *[ICH 6.10 & 6.13]* 19

8.1. Confidentiality 19

8.2. Source documents 19

8.3. CRF 19

8.4. Records retention 20

9. Safety and adverse events *[ICH 6.8]* 20

9.1. Definitions (Imputability) 20

9.2. Managing plan for adverse reactions occurring during the experiment 21

9.3. Recording adverse events 21

9.4. Reporting AE and unanticipated problems 21

9.5. Follow-up of adverse events after study ends 22

10. Study monitoring, auditing, and inspection *[ICH 6.11]* 22

10.1. Monitoring 22

10.2. Audit and inspection 22

11. Statistics *[ICH 6.9]* 23

11.1. Sample size 23

11.2. Data analysis 23

11.3. Dropouts and missing data 23

11.4. Stopping rule 23

11.5. Data entry and aberrant values 23

12. Ethical considerations *[ICH 6.12]* 23

12.1. Good Clinical Practice 23

12.2. Ethical approval 24

12.3. Informed consent 24

13. Publication plan *[ICH 6.15]* 24

13.1. Clinical trial registry 24

13.2. Publication 24

13.3. Final study report 25

14. Study finances *[ICH 6.14]* 25

14.1. Funding source 25

14.2. Conflict of interest 25

14.3. Participant's stipends 25

14.4. Insurance 25

15. Protocol amendments 26

15.1. BAC level 26

16. References 27

17. Attachments *[ICH 6.16]* 28

Résumé synoptique du protocole en français

**Nom du promoteur**	Prof Patrice Mangin, Centre Universitaire Romand de Médecine Légale, Michel-Servet 1, CH-1211 Genève-4
**Nom du produit étudié**	MedDrive©
**Description du produit**	Un logiciel de mesure comportant quatre tests neuropsychologiques pour évaluer l'aptitude à la conduite
**Titre de l'étude**	Effets de différentes alcoolémies sur MedDrive et validation de cet instrument face aux performances sur simulateur de conduite ; un essai clinique contrôlé de validation d'instrument.
**Investigateurs**	Investigateur principal : Dr Bernard Favrat<br>Co/Investigateurs : Paul Vaucher
**Lieu de l'étude**	Institut de Médecine Légale, Michel-Servet 1, CH-1211 Genève-4
**Calendrier de l'étude**	Recrutement fév 2013, entrainement jan-fév 2013, phase expérimentale fév-avr 2013
**Rationnel et objectifs**	Afin d'informer correctement les patients des effets de leur condition cognitive sur leur conduite, nous avons développé un nouvel instrument de mesure appelé MedDrive. Cet instrument devrait permettre de mieux investiguer les effets des médicaments et d'autres conditions médicales sur la conduite. Cette étude vise à mesurer l'effet de différentes alcoolémies sur les mesures de MedDrive afin de disposer de valeur de référence. Ceci permettra par la suite de tester d'autres substances dont les effets sur la conduite restent inconnus.<br>L'objectif principal est donc de quantifier l'effet de l'alcool sur les mesures de MedDrive, et de valider ces mesures face à la performance de conduite sur simulateur. Les objectifs secondaires sont l'évaluation de la fiabilité des mesures de MedDrive, de comparer les performances de MedDrive face à l'UFOV, la modélisation de ces mesures pour prédire le comportement sur simulateur, et l'étude de l'effet de l'alcool sur l'attention.
**Méthodes**	Essai contrôlé randomisé croisé dose-dépendant à double aveugle. Seize volontaires en bonne santé se verront administré aléatoirement une boisson contenant de l'alcool pour atteindre une alcoolémie de 0 g/l, 0.5 g/l, 0.8 g/l, et 1.0 g/l. Leur alcolémie sera maintenue alors qu'ils réaliseront l'ensemble des tests MedDrive ainsi que trois épreuves sur simulateur de conduite.
**Nombre de Patients**	16 participants.
**Critères de sélection**	Critères d'inclusion :<br>Détenteur d'un permis de conduire depuis au moins deux ans.<br>Apte à la conduite.<br>Agé de 20 à 40 ans.<br>Parlant couramment le français.<br>A déjà volontairement subi une alcoolémie de 1.0 g/L (6 unités en 3 heures) durant les trois mois précédents.<br>Critères de non inclusion :<br>Prise de médicament influençant la conduite.<br>Dépendance à un psychotrope.<br>Présence d'une maladie psychiatrique influençant la conduite.<br>Manifeste une maladie du voyage lors de la conduite sur simulateur.<br>Présente des antécédents de crise d'épilepsie.<br>Mauvaise tolérance à l'alcool<br>Grossesse, allaitement.
**L'instrument étudié**	MedDrive a été développé par notre département en étroite collaboration avec le Département Universitaire de Médecine et Santé Communautaire du CHUV. La programmation du logiciel a été réalisée par la haute école d'ingénieur et de gestion du canton de Vaud. MedDrive a été conçu pour évaluer les fonctions cognitives de bases nécessaires à la conduite. Il comporte quatre épreuves sur ordinateur permettant de mesurer la durée de traitement de l'information visuelle centrale et périphérique, la charge lié à la double tache, la vitesse exécutive de réponse, le gain par alerte, le gain par orientation, la vitesse d'exécution de détection de mouvement après transfert d'attention, la précision de la mémoire spatiale et la dégradation dans le temps de celle-ci.. L'analyse intermédiaire des données de l'étude normative effectuée sur des séniors (≥ 70 ans ; n=120), montre une très bonne corrélation de nos mesures avec l'âge et l'état cognitif.

**Substance administrée**	0.8g/kg, 0.64g/kg, 0.5g/kg, 0g/kg d'éthanol (95%) dilué dans du jus de canneberge sont administrés par voie orale en cinq doses de 100 ml prises toutes les deux minutes. L'alcoolémie est alors maintenue tout au long de l'expérience (1h) grâce aux mesures prises avec un éthylomètre.
**Produit de référence**	Jus de canneberge ne contenant pas d'alcool. Afin de rendre le produit indiscernable de celui avec de l'alcool, 5 ml d'éthanol sont versés sur un coton apposé sur le couvercle du verre afin de produire l'odeur d'éthanol lorsque le participant ingurgite le liquide.
**Evaluation de l'efficacité de l'instrument de mesure**	Réponse dose-effet des mesures MedDrive face aux différentes concentrations d'alcool. Validité de concurrence face à la performance sur simulateur de conduite (déviation latérale en conduite et temps à l'impact en tache de poursuite) sur un parcours standardisé de 15 min. Validité de concurrence face a l'UFOV, un test neuropsychologique sur ordinateur.
**Evaluation de la tolérance**	La tolérance au simulateur (maladie de voyage) se fait à l'aide d'un questionnaire (MSAQ) lors de chaque visite. L'alcool est une substance dont la toxicité est connue. Les risques liés à une alcoolémie de 1.0 sont un risque accru de somnolence, un déficit d'attention, des troubles d'équilibres, des possibles nausées voir vomissements. Ces effets sont cependant temporaires et ne persistent pas. Les participants sont donc gardés en surveillance pendant 3h après la fin de l'expérience avant de vérifier que leur alcoolémie est bien < 0.5‰. Une étude pilote effectuée sur 107 participants séniors (≥ 70 ans) ainsi qu'une étude normative sur 120 séniors n'ont montré aucun effet secondaire indésirable lié à l'utilisation de MedDrive.
**Méthode statistique**	La taille de l'échantillon a été mesurée pour assurer la détection d'une taille d'effet de 1.25 avec une puissance a 0.8 et un seuil de signification de 0.05. Après avoir vérifié la linéarité de l'association entre le l'alcoolémie et les mesures de MedDrive, nous effectuerons une régression linéaire permettant de quantifier cette association. Une régression linéaire ajustant pour le niveau d'alcoolémie et ajoutant un effet fixe pour tenir compte du manque d'indépendance des mesures venant des mêmes participants sera utilisée pour mesurer la force de l'association entre MedDrive et les mesures du simulateur. Pour quantifier la reproductibilité des mesures MedDrive, nous utiliserons les 5 premières séries de mesures effectuées par chaque participants lors de l'entrainement et calculerons l'ICC(2,1). Les analyses seront effectuées en per protocole et le seuil de signifiance sera fixé à 0.05 sans correction de Bonferroni vu la corrélation élevée entre chacune d'elles.

Study procedure

Schedule for each participant

	Preparation visits			Testing visits			
		Visit1		Visit2	Visit3	Visit4	Visit5
	Pre-visit (E-mail)	≅D-30	D-15 to D-1	Day 1	Day 8	Day 15	Day 22
Information	X						
Eligibility		X					
Consent		X					
Status		X					
Instructions		X					
Allocation				X			
Breathalyser prior to administration							
Administration of OH / placebo				X	X	X	X
Breathalyser				X	X	X	X
MedDrive		X	X	X	X	X	X
UFOV		X		X	X	X	X
Simulator		X		X	X	X	X
Side effects and simulator sickness		X		X	X	X	X
Sobering period				X	X	X	X

Planning for visits 2-5

	Preparation	OH absorbtion	Simulator	MedDrive	Secondary effects	Sobering up
**Duration**	5’	45’	30’	30’	10’	3h

List of Abbrevations

AE	Adverse Event
ANT	Attention Network Task
AR	Adverse Reaction
BAC	Blood Alcohol Concentration
CHUV	Centre Hospitalier Universitaire Vaudois
CRF	Case Report Form
EC	Ethical committee
GCP	Good Clinical Practice
HUGE	Hopitaux Universitaires de Genève
ICC	Intra-Class Correlation Coefficient
ICH	International Conference on Harmonisation
SAE	Serious Adverse Event
SUSAR	Suspected Unexpected Serious Adverse Reaction
TSC	Trial steering committee
UFOV	Useful Field of View
UMPT	Unité de Médecine et de Psychologie du Trafic

1. Background *[ICH 6.2]*

1.1. Background

Driving is believed to be one of the most complex common task people in developed countries have learned to do. It is therefore affected by cognitive disorders before most other activities.[1] Not only does age affect driving performances, but also medicinal drugs.[2] The number of patients on psychotropic drugs is increasing year after year inevitably leading to polypharmacy and an increased number of patients become unfit to drive.[3-5] This has led some countries, like France, to provide clear indications on drug packages about their effect on driving.[6] However, collecting grounded information on effects of medication on driving performance remains difficult. Most psychotropic drugs' effects on driving have not been measured in experimental designs.[6] Furthermore, neuropsychological tests that are sometimes used to test drugs[7,8] are not those that relate most to driving performances.[9-11] Recent reviews therefore emphasize the need to develop new adapted instruments to assess cognitive functions related to driving.[12,13]

Studying effects of drugs on driving performance remains challenging. Merging principles from neuroscience, cognitive psychology, and ergonomics provide a solid translation approach to study the complexity of multiple cognitive processes engaged during driving.[14,15] Computational neuroergonomics has shown promising results in its capacity to model cognitive functions and predict driving difficulties.[16,17] This has now made it possible to translate results from computed tests, such as the UFOV[18] or the ANT,[19] to real-life situations that can be observed when driving.[14] Finally, recent technological advances in home-computer processing time and in improvements in neuroergonomic modelling have opened the field of cognitive screening for effects of medication on driving in primary care settings. This has led us to develop a new instrument to detect effects of drugs on cognition functions indispensable for driving. The aim of this study is to validate MedDrive's performance in detecting known effects of alcohol on cognition and driving performance.[20-22]

1.2. Measuring instrument under study

MedDrive is a software that runs on personal computers on either Windows or Mac OS. It includes four computed neuropsychological tasks. The three first tasks are inspired from the UFOV, and the attention network task (ANT). The UFOV has been shown to be one of the best predictors of driving difficulties. It is however designed for senior drivers and is not adapted to be used with younger drivers.[9,11,18,23] Using Posner's model of attention,[24-26] the ANT has been shown to measure different dimensions of attention than the UFOV.[19] The ANT uses a single stimulus to measure simultaneously top-down arousal related to alertness or orientation, and frontal executional modulation related to coherent or incoherent visual information. We have however decided to separate these measures as it has been shown that they tend to interact one with another.[27-29] This has made it possible to improve the design of the paradigm and integrate movement detection instead of shape discrimination. The fourth task investigates spatial working memory decay.[30,31] Visual processing, attention, executive functions, and working memory have all been recognised as essential for driving.[32-39]

MedDrive was designed to be used for both experimental designs and clinical applications. Task are standardised but the order of subtasks, the location or nature of targets remain nevertheless unpredictable, as we have used multilevel block

randomisation. Shapes, size, duration and instructions have been calibrated to optimise the precision of the instrument. From collected data, we were able to model learning effects during tasks and improve MedDrive's ability to consistently measure the same value. MedDrive also adapts the image size to different screen resolution and pitch for the size of the image to always be constant independently of the screen. The software exports both summary measures for each task and detailed results for more specific analysis to be made. The underlying concepts are grounded, transparent, and made available to all future users in the instruction book.

We have already tested the software on more than 200 senior drivers (≥ 70 years). As expected, these unpublished results show that MedDrive has better performances in predicting effects of age, and state of cognition compared to the UFOV. From 208 participants, only one interrupted the measures on MedDrive. This participant complained of drowsiness due to the repetition of the test. Three patients with recent ophthalmological intervention and under treatment reported eye fatigue. No patient reported dizziness, headache, or gastro-intestinal disorders. There is no reason to believe this instrument could intervene during pregnancy or breastfeeding. However, as we are to administer alcohol to participants, measures will be taken to prevent us testing MedDrive's performances on pregnant women.

1.3. Risks related to alcohol consumption

As we are measuring the responsiveness of MedDrive to different blood alcohol concentrations (BAC), participants will each have their BAC at 0.0 g/L, 0.5 g/L, 0.8 g/L, and 1.0 g/L in a random order week after week. In this study, alcohol is by no mean tested as a therapeutic agent. It is however important to report known risks related to alcohol consumption.

1.4. Rationales

To improve patients' security, there is a need for a reliable and valid measuring instrument to assess effects of drugs on driving performances at a neuropsychological level. This study should make it possible to validate and promote a new instrument developed by our department. This instrument was designed to help us model and understand effects of drugs on psychometric functions. Preliminary results on senior drivers are sufficiently promising for us to believe MedDrive will not only serve research but could also help physicians evaluate effects of polypharmacy on their own patients.

This field of research will certainly become of utmost importance over the next decade given the projected increase in older drivers coupled to polypharmacy.

2. Study objectives *[ICH 6.3]*

The primary objectives are to quantify effects of different BAC on MedDrive's summary measures and validate them against performances on the driving simulator.

Secondary objectives are measuring the reliability of MedDrive following repeated measures during the training phase, compare MedDrive's performances in measuring effects of different BAC against the UFOV, and model MedDrives measures to predict behaviour on the simulator. Finally, this study also includes a nested experimental study measuring effects of alcohol on attention. This nested study's primary objective is to test whether alcohol consumption decreases our ability to pay attention to both central and peripheral visual stimuli and whether this can be modulated by expectations.

3. Study design *[ICH 6.4]*

3.1. General design

This validation trial relies on a four-way, dose-response, crossover, double blind, placebo-controlled, randomised study design. This means each participant serves as his/her own control (this is possible as alcohol can easily be washed-out). The order in which each participant's BAC is controlled is unpredictable, and both the assessor and the participant are blinded to how much alcohol was administrated.

3.2. Primary and secondary endpoints

This study does not aim to assess the efficiency of a procedure but to measure the responsiveness of a measuring instrument to different BACs. Therefore all summary measures from MedDrive are equally important.

3.3. Randomisation

For each participant, the order of BAC administration will be defined in advance using random digits from a table. Starting at a random point, the statistician will link four successive random number to the four different BAC levels (0.0 g/L to 1.0 g/L). The random numbers will then be reordered from the smallest to the largest number thereby placing the BAC levels in a random order. Each successive visit is then associated to the BAC level of this random order. Each BAC is then placed in a sealed envelope that is labelled (Participant ID and visit number). Each envelope is to be opened 1 hour before each visit by the person preparing the beverages. The same person measures the BAC using a breathalyser but maintains both the assessor and the participant blinded to results. A similar procedure will be used to decide in which order the participant is to run the tasks between the simulator and MedDrive.

3.4. Blinding

For the assessor and the participant to remain blinded to the BAC level, procedures are identical independently of the amount of alcohol administrated. The frequency of measures using the breathalyser, the amount and frequency of liquid absorption, and the washout period are all identical. To administer the beverage, we will use a 250 ml glass will have an open lid on which 5 ml of ethanol can be placed on a cotton surface before filling the glass. The participant is asked to breathe in by the nose before swallowing the liquid. The smell of ethanol will prevent them from smelling or tasting the presence of ethanol in the liquid. The person administrating the drinks will wash their hands with alcohol based liquid soap before each manipulation of the cup thereby preventing the participant from smelling the content before adding the 5ml of alcohol on the lid.

3.5. Duration of the study for participants, early withdrawal, and unblinding procedure

The study lasts one month. Participants are to attend the research centre once a week at the same time. Participants can withdraw early. These are to be replaced by a new participant. Therefore, expecting a dropout rate of 10%, we plan on screening and training 18 participants to easily replace dropouts. Unblinding will only occur if a serious adverse event occurred and for legal purposes it is essential for the participant to know if he was still under the effect of alcohol at the time of the event.

3.6. Source documents and Case Report Form

The CRF includes all collected data except those provided by MedDrive, and the simulator. For these sources, a daily backup will prevent this data from been lost. A copy of the CRF is attached to this document.

4. Study participants *[ICH 6.5]*

Screening for eligibility will be done by the principle investigator or the co-investigator supervised by a physician working at the Unit of Traffic Medicine and Psychology.

4.1. Inclusion criteria

Participants have to be:

- Aged 20 to 40 years
- Obtained drivers license at least 24 months before
- Fit to drive
- Consumed at least once six units of a beverage with alcohol at a single occasion during the previous six months

4.2. Exclusion criteria

Participants must not be:

- Under the influence of a medicinal drug affecting their driving performance
- Suffer from a psychiatric condition affecting driving performances
- Suffer from simulator sickness
- Presenting criteria (ICD-10) of alcohol dependence.
- Pregnant or breastfeeding
- Intolerant to alcohol defined by having either headaches or digestive disorders for quantities of alcohol that do not seem to bother other people.

4.3. Early withdrawal

As this study is per protocol, any major deviation from the protocol can lead the investigator to ask the patient to withdraw early. Major deviations are taking psychoactive substances within the period when we are not supposed to on more than one occasion, not been able to undergo tasks due to simulator sickness on more than one occasion, and for women having had unprotected sexual intercourse during the study period.
Once a participant has withdrawn, they are to be replaced by a backup participant who will have already followed the training. This person can start at the following week.

4.4. Subject recruitment and screening

Participants are to be recruited by posting recruitment forms within the university of Geneva. The recruitment form is attached to this document.

5. Material under investigation (diagnostic instrument) *[ICH 6.6]*

5.1. MedDrive

MedDrive has been developed following the most recent advances in neuropsychology and behavioural science. It measures processing speeds and accuracy for distinct neural networks and cognitive functions related to driving. Needs and expectations for a new instrument were investigated by a qualitative study questioning experts, primary care physicians and senior drivers. Relying on systematic reviews of neuropsychological tests and driving performances, we conceived four new computed tasks (Figure 1).

5.2. Development

Three international experts provided face validity. Instructions, learning functions, and length of tasks were defined by having 105 senior drivers run the tasks. Concurrent validity was then assessed on 120 new senior drivers. We then compared MedDrive's measures to on-road performance, age, MoCA, trial making task, and the UFOV. Details on the paradigms used are provided in Figure 2. The preliminary analysis shows tasks other than Task2 to be correlated to cognitive state (MoCA) and to age. Task1 and 2 show promising results for association to on-road evaluation.

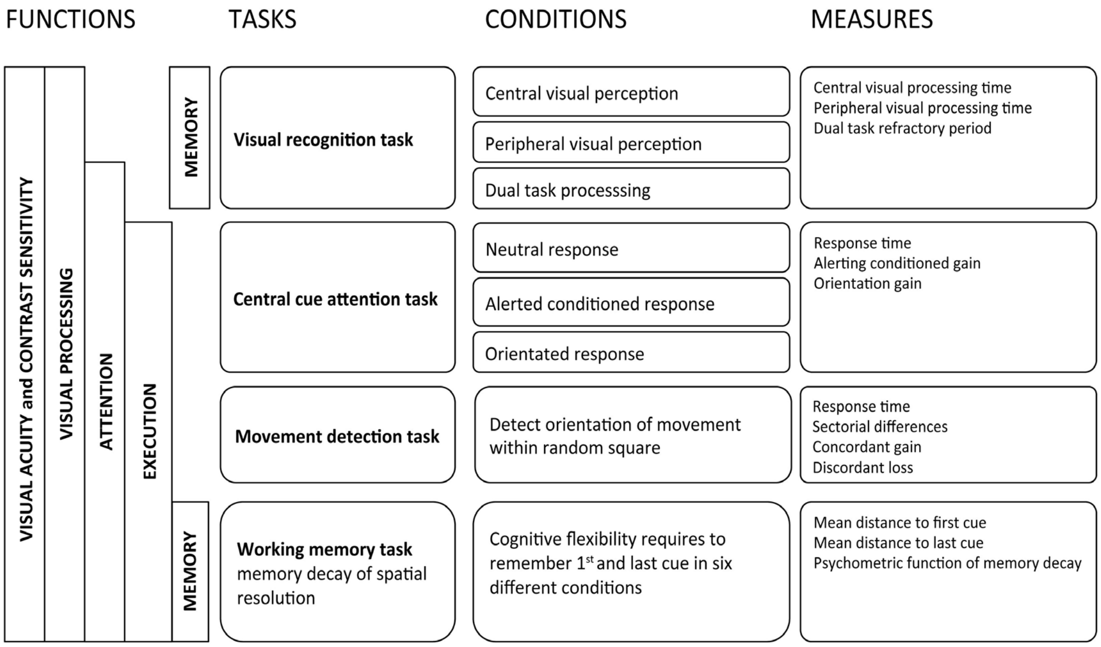

Figure 1: Tasks and measures included in MedDrive

To prevent any learning effect, participants are to practice tasks five times before starting the experimental phase. This can be done at the CMU or on their home computer.

5.3. Testing effects of expectations on visual processing speed

During the four visits during which participants can be under the influence of alcohol, they will be asked to run task 1 twice. Once each subtask run in a random order, and once each subtask run separately.

5.4. Incompatible treatment or behaviour

As MedDrive is believed to be sensitive enough to detect effects of drugs, participants are asked not to drink alcohol or any other psychoactive substance 24h before the time of their visit. For coffee this period has been reduced to four hours and for smoking to two hours. Participants are asked to report any substance taken within the previous 48h or any new medication. If those interfere with driving performance, the visit is cancelled and postponed.

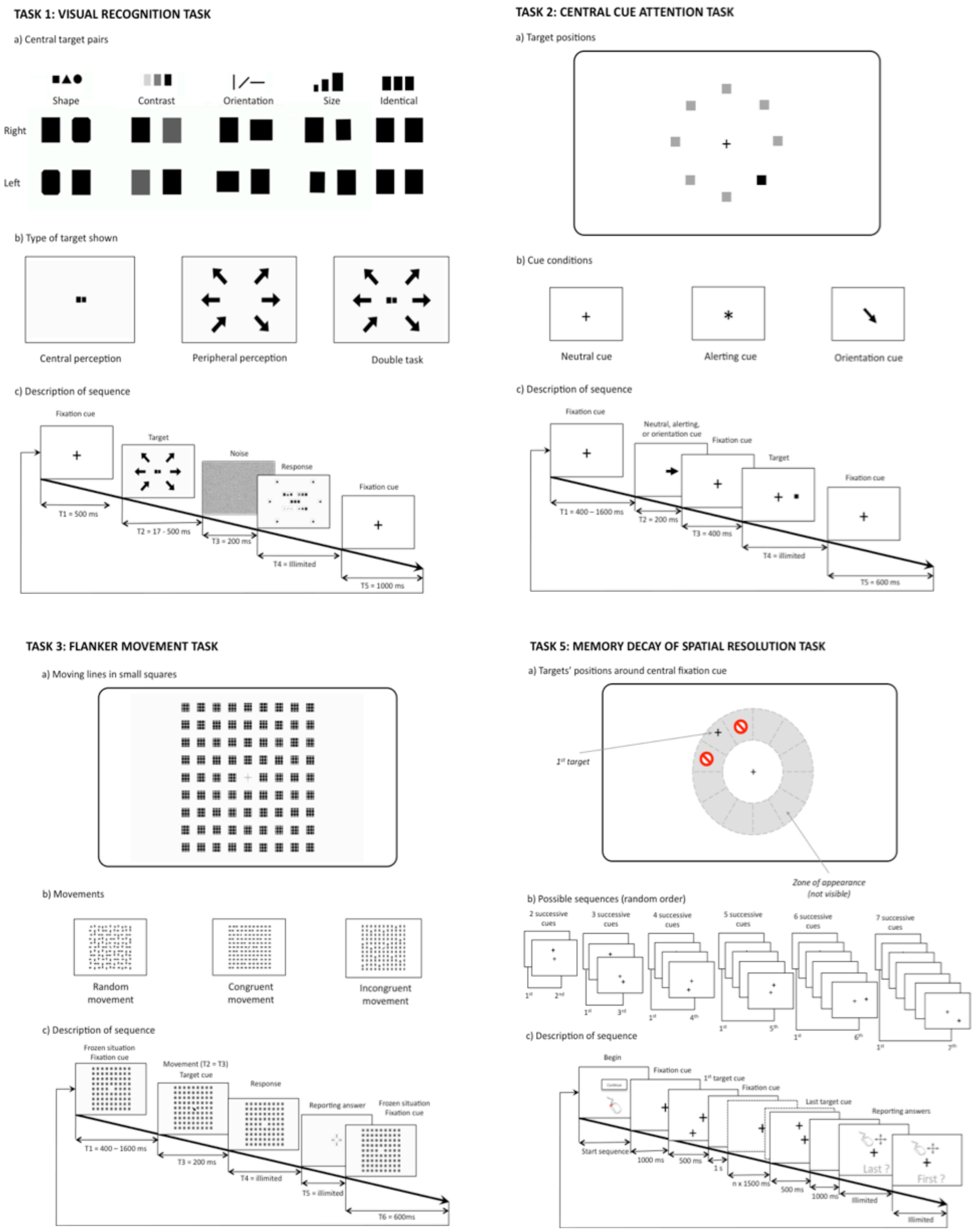

Figure 2: Paradigms used in MedDrive

6. Administration of Alcohol

We reproduced the method used by Veldstra and al.[40]

6.1. Preparing and administrating the beverage

During a 45 min period, we will ask participants to drink 500ml of cranberry juice, 100 ml at a time each 5 minutes. Depending of the allocation, these drinks will contain more or less alcohol. Pure ethanol will be used mixed with the beverage. The amount of alcohol to dilute in the drink will be calculated using Widmarks formula. Participants will move to the simulator room 20 minutes after having finished drinking the first 500 ml. As two participants are seen simultaneously, each cup will be labelled with the participant's id number.

6.2. Control

On one occasion, participants will receive no alcohol in their beverage. They will nevertheless undergo the same protocol as if there drink did contain alcohol.

6.3. Maintaining BAC

To maintain the BAC level, we will use Widmark's formula and provide 100 ml cranberry juice every 20 minutes with the amount of necessary alcohol. The person administrating drinks will hand over the drinks and make sure the participants breaths in before taking a sip, thereby inhaling alcohol vapour from the lid and keeping them blinded to the content. BAC is to be monitored using a breathalyser.
After the driving task, the participants will be asked to stay in a waiting room for three hours. During this period, they will answer the questionnaire on simulator sickness and effectiveness of blinding, and then they will be free to read, work, or use a computer. After three hours, their BAC will be estimated using a breathalyser. Only once their BAC is below 0.5‰ will they be authorised to leave.

6.4. Pregnancy and breastfeeding

At inclusion, participants who breastfeed or those with a positive pregnancy test are excluded. During the trial, female patients are asked to report any unprotected sexual intercourse. Reporting such behaviour will lead to the patient having to withdraw from the study.

7. Study outcomes *[ICH 6.7]*

Other than the effects of alcohol on MedDrive, which could be considered as our primary outcome, we will also measure effects of alcohol on driving performances on the simulator, on the UFOV and on the trial making task (TMT).

7.1. Simulator

We reproduced the method used by Veldstra and al.[40]
"Participants are required to complete test rides in a (fixed-base) driving simulator consisting of a mock-up car with original controls (three pedals, clutch, steering wheel, safety belt, indicator and hand brake) linked to a dedicated graphics computer, registering driver behaviour while the road environment and dynamic traffic are computed at 30 Hz+. Participants have a 180° view of the road environment. Other vehicles in the simulated world interact with each other and the simulator car

autonomously, and behave according to hierarchically structured decision rules that are based on human driving behaviour.[41]"
The participants will be asked to perform a road tracking task. In this task, they are requested to drive on a straight road. The standard deviation from the lateral position is then measured during the entire task. The second task is a more complex driving performance called car following. The participant is requested to follow a car. This car has a random sinusoidal speed change. The simulator calculates the style of following by assessing gain and delay.[42] A third task consists of simultaneously following a car and responding to peripheral stimulus. This dual task will provide information on balanced attention between central and peripheral fields and effects of alcohol.

7.2. UFOV

We will use UFOV-7 running on a portable PC with Windows XP to measure processing speed, selective attention, and discrimination. As mentioned in the introduction, the UFOV has been shown to be the most promising instrument in predicting driving difficulties.[9,11,43]

7.3. TMT

The trial making task is a pen and paper neuropsychological test that assesses visual seeking skills, working memory, mental flexibility, and executive function. The purpose of using this task is to identify limitations of such instruments in experimental designs (vulnerable to learning effects).[44,45]

8. Data handling and keeping *[ICH 6.10 & 6.13]*

8.1. Confidentiality

Information about study subjects will be kept confidential and managed according to the requirements of the LPD, LPT, and, State Health laws in accordance to GCP from the ICH. Participants will be informed on what protected health information will be collected, who will have access to that information and why, and who will use or disclose that information, and their rights to revoke their authorization for use of their protected health information.

8.2. Source documents

There are four sources documents for this study. The first is the paper-form CRF. The three remaining sources are informatics. These consist of the output files from MedDrive, the UFOV, and the driving simulators. All these files are conserved for quality control and audits up to 10 years after the end of the trial.

8.3. CRF

The CRF is the primary data collection instrument. The CRF for the first visit (CRF-P) and for the four following visits (CRF 1-4) are annexed to this document. All data requested on the CRF must be completed in blue or black ink. Missing data needs to be explained. Non-applicable items must be completed with N/A. Corrections to errors must be made by adding a single line through the incorrect entry and corrected entry written above. The correction must be dated and initialled.

8.4. Records retention

It is the investigator's responsibility to retain essential documents (source documents) for at least 10 years after the end of the study. These are to be conserved in a fire safe and locked file holder.

9. Safety and adverse events *[ICH 6.8]*

Investigators are responsible of correctly reporting unanticipated problems involving risk to participants or others, or any adverse events. The following sections describe how these are to be recorded and reported.

9.1. Definitions (Imputability)

Unanticipated problems are any incident, experience, or outcome that was unexpected in nature, severity, or frequency, that was possibly related to participation in the research, and that suggests that the research places participants or others at greater risk of harm.

An **adverse event** (AE) is any symptom, sign, illness or experience that develops or worsens in severity during the course of the study. Pre-existing conditions are recorded during screening. Intercurrent illnesses or injuries should be regarded as adverse events. Abnormal results of diagnostic procedures are considered to be adverse events if the abnormality:

- results in study withdrawal
- is associated with a serious adverse event
- is associated with clinical signs or symptoms
- leads to additional treatment or to further diagnostic tests
- is considered by the investigator to be of clinical significance

The **severity** of each adverse event is the assessment of the degree to which participants developed symptoms as a result of the intervention.

- Grade 1: Non-Severe
  Medical intervention is required but lack of such would not result in permanent damage or impairment of a bodily function.
- Grade 2: Severe
  Inpatient hospitalization or prolongation of hospitalization is directly attributable to the adverse event, persistent or significant disability or incapacity of the patient occurs as a result of the reaction, or a medical or surgical intervention is necessary to preclude permanent damage or impairment of a body function.
- Grade 3: Life-threatening
  Major intervention (intubation, intensive care) required following the manifestation of the adverse event.
- *Grade 4: Death
  The recipient died as a result of the adverse event.

Serious adverse events (SAE) are any adverse event that is:

- fatal
- life-threatening
- requires or prolongs hospital stay

- results in persistent or significant disability or incapacity
- a congenital anomaly or birth defect
- an important medical event

Imputability is the assessment of the relationship between MedDrive and the adverse event. It can either be definite, probable, possible, doubtful, ruled out, or not determined.

The **adverse event reporting period** is the period from the initiation of any study procedures to the end of the study follow-up. For this study, the follow-up period ends at the end of the 4th visit once the participant's BAC is below 0.1 g/L. At the last scheduled visit, participants are instructed to report any subsequent event(s) that the subject, or the subject's personal physician, believes might reasonably be related to participation in this study.

9.2. Managing plan for adverse reactions occurring during the experiment

In case of physical problems or faintness occurring during the experience, the participant is to cease the exercise they are doing and no more beverage are to be administered.
The principle investigator is contacted within 5 minutes to decide whether hospitalisation is required or not. If this was to be the case, an ambulance is to be called (144). All expenses related to the transport and the care related to this event and its consequences are covered by the study's insurance.
If hospitalisation is not necessary, the participant is then brought to a consultation room where they can lay down. They are to be assisted at all time until they feel well enough to move to the cafeteria (waiting room). Once their BAC is below 0.5‰, if they still feel physical problems or faintness, the research assistant is accompany them to their home and assure they can obtain assistance in case of need.

9.3. Recording adverse events

At each contact, the investigator will seek information on adverse events. These are recorded immediately on the CRF. Related signs, symptoms, and abnormal diagnostic procedures results are to be recorded in the CRF, they can however be grouped under one diagnosis. All adverse events occurring during the study period must be recorded.

9.4. Reporting AE and unanticipated problems

Sponsor
Any study-related unanticipated problem posing risk of harm to subjects or others, and any type of serious adverse event, will be reported to the study sponsor by telephone within 24 hours of the event. To report such events, a Serious Adverse Event (SAE) form will be completed by the investigator and E-mailed to the study sponsor. The investigator will keep a copy of this SAE form on file at the study site.

Serious adverse events will be reported by E-Mail and phone to:

Patrice Mangin Patrice.Mangin@hcuge.ch +41 22 379 59 02

Within the following 48 hours, the investigator will provide further information on the serious adverse event or the unanticipated problem in the form of a written narrative report. This should include a copy of the completed Serious Adverse Event form

(annexed document), and any other diagnostic information that will assist the understanding of the event. Significant new information on on-going serious adverse events are to be provided promptly to the study sponsor

Ethical committee
Within 10 days following the time the investigator became aware of an adverse event, he will report any adverse event that is unexpected and related to the research procedure to the central ethical committee. Events will be reported within the next 24h when death is unforeseen and could indicate others are at risk of harm, and within 72h for any death regardless of whether it is related to study participation or not.

The ethical committee will be notified by E-mail at the following address:

RechercheEtreHumain.CommissionEthique@hcuge.ch

Copies of each report and documentation of ethical committee notification and receipt will be kept in the Clinical Investigator's study file.

9.5. Follow-up of adverse events after study ends

The clinical course of each event will be followed until resolution, stabilization, or until it has been determined that the study treatment or participation is not the cause. Serious adverse events that are still on-going at the end of the study period will be followed up to determine the final outcome. Participants will be monitored for adverse events after their 5th visit over the phone one week after their last visit. Any serious adverse event that occurs after the study period and is considered to be possibly related to the study treatment or study participation will be recorded and reported.

10. Study monitoring, auditing, and inspection *[ICH 6.11]*

10.1. Monitoring

Once the study is under way, the investigator is to monitor the study five times, once after each set of height participants provide their output file to verify training procedures, and again after they have all undergone their first visit, and a second after all height first participants have undergone all visits. The monitoring plan includes checking CRFs for missing data, incomplete data, checking output files from devices, verifying time allocation and BAC control.

10.2. Audit and inspection

The diagnostic instrument we are validating cannot be registred by Swissmedic. Therefore, this government regulatory body is not entitled to run an audit for this study. The investigator will permit study-related monitoring, audits, and inspections by the ethical committee, the sponsor, government regulatory bodies, and University compliance and quality assurance groups of all study related documents (e.g. source documents, regulatory documents, data collection instruments, study data etc.). The investigator will ensure the capability for inspections of applicable study-related facilities.

11. Statistics *[ICH 6.9]*

11.1. Sample size

We have powered the study to detect an effect size of 1.25 using an ANOVA with four levels of factor. With a power set at 0.8 and a significance level at 0.05, this requires including 16 participants.

11.2. Data analysis

Effects of alcohol on each of MedDrive's output is provided by 1,920 measures. Summary measures of the thirty repeated measures are already provided by MedDrive. Changes in outcomes between control (0 g/L OH) and other concentrations will be compared using random effect linear regression to calculate an averaged least-square mean for each different concentration of OH and adjusting for lack of independence for measures coming from the same subject. Level of significance will be set at $p<0.05$. We will also test linearity of dose-response effect using likelihood ratio tests. Test-retest reliability will be measured using ICC(2,1) with CI95%.
Using known properties of neural transmission, measured times of response or perception from MedDrive will be used to model and predict lateral deviation and distance to leading vehicle measured by the driving simulator. The model using MedDrive will then be compared to the model using results from the UFOV.

11.3. Dropouts and missing data

The analysis is per protocol. Patients who dropped out early are to be replaced by a new participant. When available, reasons for dropping out will be reported. Special care will be taken to prevent any missing data. If data is to be missing totally at random, we will then use multiple imputation.

11.4. Stopping rule

No interim analysis is planned. As there are no expected serious adverse events related to the intervention, we do not plan any stopping rules. In the presence of a serious adverse event, the trial steering committee is to be notified and can decide to stop the trial for security reasons. The trial cannot be stopped early for futility or efficacy.

11.5. Data entry and aberrant values

Double data entry will be used to prevent transcription errors. Clear instructions are provided on values to be considered as aberrant. When possible, participants will be contacted to correct aberrant values. Those that cannot be corrected will be considered as missing.

12. Ethical considerations *[ICH 6.12]*

12.1. Good Clinical Practice

This study is to be conducted according to international standards of Good Clinical Practice (International Conference on Harmonization guidelines), applicable government regulations and Institutional research policies and procedures.

12.2. Ethical approval

This protocol and any amendments will be submitted to the Ethics Committee (EC) of the HUG, in agreement with local legal prescriptions, for formal approval of the study conduct. A copy of their decision will be added to the Clinical Investigator's study file and provided to the sponsor before commencement of this study.

12.3. Informed consent

All subjects for this study will be provided a consent form describing this study and providing sufficient information for subjects to make an informed decision about their participation in this study. Both information sheet and consent form are attached to the protocol. They will be submitted with the protocol for review and approval by the EC for the study. Participants and the investigator-designated research professional obtaining the consent will sign the consent forms.

13. Publication plan *[ICH 6.15]*

13.1. Clinical trial registry

This study was registered in January 2012 prior to recruitment on ClinicalTrials.gov with the identifier: NCT01781273. An amendment was brought on the 12th February 2013 to raise the age limit for inclusion from 35 to 40 years of age.

13.2. Publication

At least four publications are expected using data drawn from this study. These publications have been summarised in table 1.

Target Journal	*1st author*	*Senior author*	*Co-authors*	*Topic*
PLoS Med	Vaucher P	Favrat B	Veldstra J, Broers B, Annoni JM, Mangin P, Petremand E, Bindschaedler C	Testing effects of drugs and age on fitness to drive: development and validity of a personal computer based neuropsychological instrument - MedDrive
Accid Ann Prev	Vaucher P	Favrat B	Mangin P	Psychometric performance of MedDrive against the UFOV in assessing fitness to drive
Traffic Inj Prev	Vaucher P	Favrat B	Mangin P	Queuing network modelling of attention and driving performances
Alcohol Res Health	Vaucher P	Bindschaedler C	Cardoso I, Favrat B, Mangin P	Effects of alcohol on attention, visual processing, and driving performance

Table 1: Study publication plan

Neither the complete nor any part of the results of the study carried out under this protocol, nor any of the information provided by the sponsor for the purposes of performing the study, will be published or passed on to any third party without the consent of the study sponsor. Any investigator involved with this study is obligated to provide the sponsor with complete test results and all data derived from the study.

13.3. Final study report

No specific study report is planned for this study. Results from this study and those collected during the GARAge study (400 screened senior drivers) are to be include in Paul Vaucher's thesis.

14. Study finances *[ICH 6.14]*

14.1. Funding source

This study is financed by a grant accorded by the DUMSC, CHUV, and by research funds from our own department.

14.2. Conflict of interest

Legal rights over MedDrive are handled by UNITEC from the university of Geneva. MedDrive been a software, it is covered automatically by copyright. Etienne Pétremand, the programmer, ceded his rights under contract. Paul Vaucher and Bernard Favrat therefore detain copyrights for MedDrive. The product is therefore issued from both the University of Geneva and the CHUV. All benefits are usually shared 50% for the institutions and 50% for the creators. Bernard Favrat and Paul Vaucher have however both renounced receiving any financial benefit from future potential financial incomes related to the software. Patrice Mangin reports not having any conflict of interest.

14.3. Participant's stipends

Volunteers receive a stipends of CHF 100.- for each visit which lasts four hours each.

14.4. Insurance

Once the study protocol accepted, the EC is to formulate a request for an insurance policy to Me Marescotti. The study will be able to start once the confirmation of this insurance coverage can be added to the Clinical Investigator's study file.

15. Protocol amendments

15.1. BAC level

On Thursday 28th February, the first two participants had their BAC rise to 1 g/L. This was made possible by providing them with respectively 150 mL and 104 mL of ethanol (96%) over a two-hour period. Both participants fell sick (nausea and vomiting) within the 30 minutes that followed the measures on the simulator and on MedDrive. Their state stabilised after 1h30’ and they did not suffer from symptoms the following day.
The TSC was notified and decided that for the participant’s safety and comfort, we were to change the procedure to prevent participants BAC to rise to 1 g/L. Allocation sequence we therefore modified for all participants (Table 2). The ethical committee was notified of this decision on Monday 4th of March.

Initial allocation BAC (g/L)	Modified allocation BAC (g/L)
0	0
0.5	0.5
0.8	0.65
1.0	0.8

Table 2: Changes of target BAC for allocated sequences.

All participants were informed of this modification both orally and in writing.

16. References

1. Rizzo M. Impaired driving from medical conditions: a 70-year-old man trying to decide if he should continue driving. *JAMA.* Mar 9 2011;305(10):1018-1026.
2. Orriols L, Salmi LR, Philip P, et al. The impact of medicinal drugs on traffic safety: a systematic review of epidemiological studies. *Pharmacoepidemiol Drug Saf.* Aug 2009;18(8):647-658.
3. Moloney ME, Konrad TR, Zimmer CR. The medicalization of sleeplessness: a public health concern. *Am J Public Health.* Aug 2011;101(8):1429-1433.
4. Gallego JA, Bonetti J, Zhang J, Kane JM, Correll CU. Prevalence and correlates of antipsychotic polypharmacy: A systematic review and meta-regression of global and regional trends from the 1970s to 2009. *Schizophr Res.* Apr 23 2012.
5. De Las Cuevas C, Sanz EJ. Fitness to drive of psychiatric patients. *Prim Care Companion J Clin Psychiatry.* 2008;10(5):384-390.
6. Riche C, Caulin C, Caron J, et al. *Medicinal products and driving.* Paris: Agence française de sécurité sanitaire des produits de santé (afssaps);2009.
7. Otmani S, Demazieres A, Staner C, et al. Effects of prolonged-release melatonin, zolpidem, and their combination on psychomotor functions, memory recall, and driving skills in healthy middle aged and elderly volunteers. *Hum Psychopharmacol.* Dec 2008;23(8):693-705.
8. Wilkinson CJ. The acute effects of zolpidem, administered alone and with alcohol, on cognitive and psychomotor function. *J Clin Psychiatry.* Jul 1995;56(7):309-318.
9. Mathias JL, Lucas LK. Cognitive predictors of unsafe driving in older drivers: a meta-analysis. *Int Psychogeriatr.* Aug 2009;21(4):637-653.
10. Reger MA, Welsh RK, Watson GS, Cholerton B, Baker LD, Craft S. The relationship between neuropsychological functioning and driving ability in dementia: a meta-analysis. *Neuropsychology.* Jan 2004;18(1):85-93.
11. Silva MT, Laks J, Engelhardt E. Neuropsychological tests and driving in dementia: a review of the recent literature. *Rev Assoc Med Bras.* Jul-Aug 2009;55(4):484-488.
12. Carr DB, Ott BR. The Older Adult Driver With Cognitive Impairment: "It's a Very Frustrating Life". *JAMA.* April 28, 2010 2010;303(16):1632-1641.
13. Martin AJ, Marottoli R, O'Neill D. Driving assessment for maintaining mobility and safety in drivers with dementia. *Cochrane Database Syst Rev.* 2009(1):CD006222.
14. Lees MN, Cosman JD, Lee JD, Fricke N, Rizzo M. Translating cognitive neuroscience to the driver's operational environment: a neuroergonomic approach. *Am J Psychol.* Winter 2010;123(4):391-411.
15. Parasuraman R, Wilson GF. Putting the brain to work: neuroergonomics past, present, and future. *Hum Factors.* Jun 2008;50(3):468-474.
16. Aksan N, Anderson SW, Dawson JD, Johnson AM, Uc EY, Rizzo M. Cognitive functioning predicts driver safety on road tests 1 and 2 years later. *J Am Geriatr Soc.* Jan 2012;60(1):99-105.
17. Liu Y, Wu C, Berman MG. Computational neuroergonomics. *Neuroimage.* May 18 2011.
18. Hoffman L, McDowd JM, Atchley P, Dubinsky R. The role of visual attention in predicting driving impairment in older adults. *Psychol Aging.* Dec 2005;20(4):610-622.
19. Weaver B, Bedard M, McAuliffe J, Parkkari M. Using the Attention Network Test to predict driving test scores. *Accid Anal Prev.* Jan 2009;41(1):76-83.
20. Borkenstein RF. *The Role of the Drinking Driver in Traffic Accidents: The Grand Rapids Study*: Steintor-Verlag; 1974.
21. Krüger H-P, Kazenwadel J, Vollrath M. Grand Rapids effects revisited: Accidents, alcohol and risk. *Alcohol, Drugs and Traffic Safety - T'95.* 1995;1:S. 222-230.
22. Rehm J, Mathers C, Popova S, Thavorncharoensap M, Teerawattananon Y, Patra J. Global burden of disease and injury and economic cost attributable to alcohol use and alcohol-use disorders. *Lancet.* Jun 27 2009;373(9682):2223-2233.
23. George S, Crotty M. Establishing criterion validity of the Useful Field of View assessment and Stroke Drivers' Screening Assessment: comparison to the result of on-road assessment. *Am J Occup Ther.* Jan-Feb 2010;64(1):114-122.
24. Posner MI, Rothbart MK. Research on attention networks as a model for the integration of psychological science. *Annual review of psychology.* 2007;58:1-23.
25. Lopez-Ramon MF, Castro C, Roca J, Ledesma R, Lupianez J. Attentional networks functioning, age, and attentional lapses while driving. *Traffic Inj Prev.* Oct 2011;12(5):518-528.
26. Jennings JM, Dagenbach D, Engle CM, Funke LJ. Age-related changes and the attention network task: an examination of alerting, orienting, and executive function. *Neuropsychol Dev Cogn B Aging Neuropsychol Cogn.* Jul 2007;14(4):353-369.
27. Fan J, McCandliss BD, Sommer T, Raz A, Posner MI. Testing the efficiency and independence of attentional networks. *J Cogn Neurosci.* Apr 1 2002;14(3):340-347.
28. McConnell MM, Shore DI. Mixing measures: testing an assumption of the Attention Network Test. *Atten Percept Psychophys.* May 2011;73(4):1096-1107.
29. Macleod JW, Lawrence MA, McConnell MM, Eskes GA, Klein RM, Shore DI. Appraising the ANT: Psychometric and theoretical considerations of the Attention Network Test. *Neuropsychology.* Sep 2010;24(5):637-651.
30. Ruchkin DS, Grafman J, Cameron K, Berndt RS. Working memory retention systems: a state of activated long-term memory. *Behav Brain Sci.* Dec 2003;26(6):709-728; discussion 728-777.
31. Olivers CN, Peters J, Houtkamp R, Roelfsema PR. Different states in visual working memory: when it guides attention and when it does not. *Trends in cognitive sciences.* Jul 2011;15(7):327-334.
32. Sherman FT. Driving: the ultimate IADL. *Geriatrics.* Oct 2006;61(10):9-10.
33. Odenheimer GL. Driver safety in older adults. The physician's role in assessing driving skills of older patients. *Geriatrics.* Oct 2006;61(10):14-21.
34. Murden RA, Unroe K. Assessing older drivers: a primary care protocol to evaluate driving safety risk. *Geriatrics.* Aug 2005;60(8):22-25.
35. Mosimann UP, Bachli-Bietry J, Boll J, et al. [Consensus recommendations for the assessment of fitness to drive in cognitively impaired patients]. *Praxis.* Mar 28 2012;101(7):451-464.
36. Messinger-Rapport BJ. Assessment and counseling of older drivers. A guide for primary care physicians. *Geriatrics.* Dec 2003;58(12):16-18, 21-14.
37. Iverson DJ, Gronseth GS, Reger MA, Classen S, Dubinsky RM, Rizzo M. Practice parameter update: evaluation and management of driving risk in dementia: report of the Quality Standards Subcommittee of the American Academy of Neurology. *Neurology.* Apr 20 2010;74(16):1316-1324.
38. Bula C, Eyer S, von Gunten A, Favrat B, Monod S. Conduite automobile et troubles cognitifs: comment anticiper? *Rev Med Suisse.* Nov 9 2011;7(316):2184-2189.
39. AMA/NHTSA. Older patients & driving. New AMA/NHTSA guide helps physicians assess patient ability. *Tenn Med.* Sep 2003;96(9):405.
40. Veldstra JL, Brookhuis KA, de Waard D, et al. Effects of alcohol (BAC 0.5 per thousand) and ecstasy (MDMA 100 mg) on simulated driving performance and traffic safety. *Psychopharmacology (Berl).* Aug 2012;222(3):377-390.
41. Van Wolffelaar P, Brookhuis K, de Waard D. A new driving simulator including an interactive intelligent traffic environment. Proceed- ings of the third international conference on vehicle navigation and information systems1992:499-506.

42. Brookhuis K, Waard DD, Mulder BEN. Measuring driving performance by car-following in traffic. *Ergonomics.* 1994/03/01 1994;37(3):427-434.

43. Devos H, Akinwuntan AE, Nieuwboer A, Truijen S, Tant M, De Weerdt W. Screening for fitness to drive after stroke: A systematic review and meta-analysis. *Neurology.* Feb 22 2011;76(8):747-756.

44. Tombaugh TN. Trail Making Test A and B: Normative data stratified by age and education. *Archives of Clinical Neuropsychology.* 2004;19(2):203-214.

45. Sanchez-Cubillo I, Perianez JA, Adrover-Roig D, et al. Construct validity of the Trail Making Test: role of task-switching, working memory, inhibition/interference control, and visuomotor abilities. *J Int Neuropsychol Soc.* May 2009;15(3):438-450.

17. Attachments *[ICH 6.16]*

1. Recruitment form
2. Information form
3. Consent form
4. CRF
5. Serious adverse event form
6. Attestation of absence of financial conflicts of interest

Formulaire d'avis du Comité d'Ethique

La Commission d'Ethique de la Recherche sur l'Etre Humain (CEREH) a bien reçu vos réponses à nos questions dans votre courrier du 13.01.2013 ainsi que les documents annexés (version 1.2 du 13.02.2013 des documents suivants : résumé en français, protocole, formulaires d'information aux patients et de consentement, CRF, annonce SAE et attestation de renoncement aux intérêts financiers du 04.01.2013) et vous en remercie. Nous avons le plaisir de vous annoncer que votre protocole est définitivement accepté.

Investigateur

Nom, prénom, titre :	Dr Bernard FAVRAT
Fonction :	Médecin adjoint agrégé responsable d'unité
Adresse :	Centre universitaire romand de médecine légale<br>Rue Michel-Servet 1 – 1211 Genève 4

Désignation du projet de recherche n° de réf. CER : **12-277**

Effets de différentes alcoolémies sur MedDrive et validation de cet instrument face aux performances sur simulateur de conduite; un essai clinique contrôlé de validation d'instrument : l'étude OH-MedDrive

☐ procédure ordinaire ☒ procédure simplifiée ☐ évaluation ultérieure

Le comité d'éthique arrête l'**avis** suivant:

☒ **A Avis positif**[1]

☐ **B Avis conditionnel**[2]

- ☐ **Evaluation ultérieure par le comité d'éthique nécessaire**:
  documents à fournir en *1 exemplaire CD + 1 exemplaires papier*
- ☐ **Information écrite au comité d'éthique suffisante**:
  documents à fournir en *1 exemplaire CD + 1 exemplaires papier*

☐ **C Avis négatif motivé**[3] **(et explication pour réexamen)** (voir ci-dessous)

☐ **D Avis justifié de ne pas entrer en matière**[4] (voir ci-dessous)

L'avis s'applique également aux autres investigateurs mentionnés dans la « demande d'évaluation », travaillant dans des sites de recherche relevant du champ de compétence du CE.

Le Comité d'Ethique : Nom(s): **Professeur B. HIRSCHEL, Président**

Genève, le 19 février 2013 Signature(s) : 21/2/2013 B. Hirschel

HUG Hôpitaux Universitaires de Genève

UNIVERSITÉ DE GENÈVE

Cherchons volontaires

pour une étude sur l'effet de l'alcool sur la conduite

L'Unité de Médecine et de Psychologie du Trafic en collaboration avec la Faculté de Psychologie de l'Université de Genève vous invite à participer à une étude expérimentale.

Sous notre supervision, cette étude est menée conjointement par Paul Vaucher, doctorant en neuroscience, et Isabelle Cardoso, étudiante en 4ème année en psychologie.

Nous cherchons 18 personnes de 20 à 35 ans en bonne santé, détenteur d'un permi de conduire depuis au moins deux ans, pour participer une fois par semaine pendant un mois (février-avril 2013) à une étude qui a lieu au Centre Universitaire Romand de Médecine Légale, au CMU, Bâtiment C, 8ème étage.

Lors de cette expérience, nous vous demanderons de boire une boisson alcoolisée pouvant faire monter votre alcoolémie à 1‰. Nous vous demanderons ensuite de conduire notre simulateur de conduite et d'effectuer une série de tests neuropsychologiques sur ordinateur. Cette étude fait l'objet d'une rénuméra-tion.

Si vous êtes intéressé(e), veuillez soit nous contacter par téléphone au numéro ci-dessous, soit nous envoyer un courriel afin que nous vous transmettions de plus amples informations.

Dr Bernard Favrat, MD, privat docent
Investigateur principale
Unité de Médecine et de Psychologie de Trafic
Centre Universitaire Romand de Médecine Légale
Michel-Servet 1
1211 Genève-4

Dr Claire Bindschaedler
Chargé de cours
Faculté de Psychologie et des Sciences de l'Education
Boulevard du Pont-d'Arve 40
1211 Genève-4

Etude OH-MedDrive
cardoso4@etu.unige.ch
079 724 46 84

Etude OH-MedDrive
cardoso4@etu.unige.ch
079 724 46 84

Etude OH-MedDrive
cardoso4@etu.unige.ch
079 724 46 84

Etude OH-MedDrive
cardoso4@etu.unige.ch
079 724 46 84

Etude OH-MedDrive
cardoso4@etu.unige.ch
079 724 46 84

Etude OH-MedDrive
cardoso4@etu.unige.ch
079 724 46 84

Etude OH-MedDrive
cardoso4@etu.unige.ch
079 724 46 84

Etude OH-MedDrive
cardoso4@etu.unige.ch
079 724 46 84

Etude OH-MedDrive
cardoso4@etu.unige.ch
079 724 46 84

Etude OH-MedDrive
cardoso4@etu.unige.ch
079 724 46 84

Formulaire d'information de l'étude OH-MedDrive

HUG Hôpitaux Universitaires de Genève UNIVERSITÉ DE GENÈVE

Unité de Médecine et de Psychologie du Trafic
Centre Universitaire Romand de Médecine Légale
Michel-Servet 1
CH-1211 Genève 4

Concerne : Effets de différentes alcoolémies sur MedDrive et validation de cet instrument face aux performances sur simulateur de conduite ; un essai clinique contrôlé de validation d'instrument.
Protocole n° 12-277, version 1.2.1 du 4 mars 2013

Madame, Monsieur,

Vous nous avez manifesté votre intérêt à participer à une étude menée par l'Unité de Médecine et de psychologie du Trafic sur l'effet de l'alcool sur la conduite et la mesure de l'attention. Nous vous invitons maintenant à lire et à conserver ce document qui contient les informations nécessaires pour vous permettre de librement décider de participer ou non.

1. Critères pour pouvoir devenir participant volontaire

Pour participer, vous devez comprendre le français, être en possession d'un permis de conduire valable depuis au moins deux ans, avoir entre 20 et 39 ans, être apte à la conduite, et avoir consommé au moins une fois durant les trois derniers mois au moins six verres de boissons contenant de l'alcool durant une même occasion. Il est également important que vous ne preniez pas de médicament pouvant influencer la conduite, que vous ne souffriez pas de troubles psychiques influençant votre conduite, que vous ne souffrez pas de maladie du voyage apparaissant lors de la conduite sur simulateur (sera évalué lors de votre première visite), et que vous n'avez pas une mauvaise tolérance à l'alcool avec l'apparition de maux de tête et/ou troubles digestifs après l'ingestion d'une quantité d'alcool qui ne semble pas affecter d'autres personnes . Finalement, pour les femmes, il est important que vous ne soyez pas enceinte et que vous n'allaitiez pas. Un test de grossesse sera réalisé lors de la première visite, et nous vous demandons d'utiliser un moyen contraceptif sûr.

2. Objet de l'étude

Les récentes revues de la littérature soulèvent le besoin de développer de nouveau instruments cliniques pour évaluer l'état cognitif et l'aptitude à la conduite. Afin de permettre de mesurer l'effet des médicaments, de l'âge, ou d'autres substances ou conditions sur la conduite, nous avons développé un logiciel, appelé MedDrive, qui comporte quatre tests neuropsychologiques. Nous cherchons maintenant à mesurer la capacité de cet instrument à détecter les effets de différentes alcoolémies et de voir si ces effets ont également une répercussion sur la performance de conduite sur simulateur. Cette étude permet également de mesurer la fiabilité des mesures de MedDrive, de comparer sa performance face à un autre test sur ordinateur appelé UFOV, et d'étudier l'effet de l'alcool sur l'attention.

3. Informations générales sur l'étude

Comme vous, quinze autres jeunes conducteurs en bonne santé vont participer volontairement à cette étude expérimentale qui va durer jusqu'en avril 2013.

Substance administrée

Nous vous ferrons boire du jus de canneberge pouvant contenir jusqu'à 10% d'éthanol (alcool). Ceci dans le but d'élevé la concentration d'alcool dans votre sang (alcoolémie) à un taux fixe allant jusqu'à 0.8‰. Ceci correspond alors à l'équivalent de 5-6 boissons alcoolisées servies dans un verre standard.

Instrument de mesure évalué par l'étude

MedDrive est un logiciel informatique qui peut être installé sur PC ou Mac. Il contient quatre tâches neuropsychologiques permettant d'étudier diverses fonctions du cerveau nécessaires à la conduite. Ces tâches se présentent sous forme de jeux simplistes un peu répétitifs. Le temps nécessaire pour effectuer les quatre tâches est d'environ 20 minutes.

Simulateur

Durant l'étude, nous allons mesurer votre performance à conduire sur simulateur. Pendant 30 minutes, vous allez donc conduire sur une route virtuelle. Nous allons également vous faire faire un autre test neuropsychologique sur ordinateur.

Double aveugle, randomisation, placebo

Pour garantir que l'effet que nous observons est bien lié à l'alcoolémie et pas à autre chose, ni la personne qui effectuera les mesures, ni vous, ne saurez la quantité d'alcool que vous aurez absorbée. A votre insu, vous allez donc avoir aléatoirement un des taux d'alcoolémie suivant : 0‰, 0.5‰, 0.65‰ et 0.8‰. Pour des raisons de sécurité, nous nous comporterons donc avec vous comme si votre alcoolémie montait à 0.8‰ à chaque fois.

Approbation du service et de la commission d'éthique

Cette étude est réalisée dans le respect de principes reconnus et bénéficie de l'accord de la commission d'éthique de la recherche sur l'être humain. Le protocole a été approuvé par le chef de service, le Professeur Patrice Mangin.

4. Caractère volontaire de la participation

Votre participation à cette étude est volontaire. Votre consentement écrit sera demandé au début de la première visite. Vous pouvez à tout moment revenir sur votre consentement à prendre part à cette étude, sans être tenu(e) de justifier votre décision. Pour votre sécurité, vous vous engagez cependant à vous soumettre à une visite médicale avant votre départ.

5. Déroulement de l'étude

Nous vous verrons à cinq reprises au 8ème étage du CMU, bâtiment C, à l'Institution de Médecine Légale. La première visite est prévue pour que nous puissions faire connaissance, répondre à vos questions, vous familiariser avec les épreuves, et vous donner le matériel dont vous aurez besoin. Durant les quatre visites suivantes qui ont lieu une fois par semaine, nous vous donnerons une boisson qui peut contenir de l'alcool et nous permettra de monter votre alcoolémie jusqu'à 1 g/L ou 1‰. Sous l'effet de ces boissons, nous vous demanderons alors d'effectuer les différentes tâches sur ordinateur et simulateur de conduite. Une période de 3h de dégrisement est alors prévue pour vous permettre de repartir en toute sécurité.

Visite 1 : Evaluation psycho-médicale d'entrée et entraînement (environ 3h)

Lors de la première visite, nous allons vous questionner et effectuer une série d'épreuves et de tests cliniques (test de la vue, test d'impulsivité, etc.) afin de pouvoir confirmer votre éligibilité et d'obtenir les données nécessaires pour décrire la population que nous étudions (environ 1h). Les résultats de ces examens vous seront restitués sous forme de bilan. Si nous remarquons quelque chose d'anormal, vous serez vu par un médecin qui vous en informera.
Après votre évaluation psycho-médicale, nous allons vous faire conduire sur un simulateur (environ 30 minutes). Ceci afin de vous familiariser avec la tâche et d'évaluer si vous souffrez de maladie de voyage ou non. Finalement, nous vous montrerons comment utiliser le logiciel MedDrive, le logiciel UFOV, et l'éthylomètre (environ 45 minutes). Durant cette première visite, il se peut que l'investigateur décide de vous exclure si vous ne répondez pas aux critères dont nous avons besoin.

Période entre la visite 1 et la visite 2

Durant les trois semaines qui séparent votre première de votre deuxième visite, nous vous demanderons d'effectuer cinq fois l'ensemble des quatre tâches de MedDrive sur un ordinateur de votre choix à domicile. Ces épreuves doivent êtres effectuées sous les mêmes conditions (même endroit, même ordinateur, même luminosité, même position, même correction optique, même état de fatigue) et réalisées sans être sous l'influence d'un psychotrope (alcool, café, cigarette, etc.). Chaque série de mesures prend environ 20-25 minutes.

Visites 2 à 5 (5h chacune)

A quatre reprises, le même jour de la semaine et à la même heure, nous vous demanderons de vous rendre au CMU pour une durée de cinq heures. Les premières 45 minutes servent à amener votre alcoolémie au taux souhaité (à votre insu). Durant cette période, nous vous questionnerons sur les évènements indésirables que vous avez ressentis durant la semaine écoulée. Pendant l'heure qui suit, une collaboratrice de recherche qui ne connait pas votre alcoolémie va vous superviser pendant que vous effectuez les tests neuropsychologiques sur MedDrive et que vous effectuez les trois parcours sur le simulateur de conduite. Votre alcoolémie sera maintenue au niveau souhaité durant toute cette période. Vous serez ensuite invité à rester pendant trois heures dans notre établissement ou vous aurez la possibilité de lire, étudier, ou utiliser un ordinateur connecté à internet. Une fois cette période de dégrisement terminée, nous nous assurerons que votre alcoolémie est bien inférieure à 0.5‰ et vous demanderons de signer une décharge comme quoi vous rentrez à la maison en utilisant les transports publics. Cette procédure sera identique indépendamment de la quantité réelle d'alcool que vous aurez consommée.

6. Modification du comportement habituel

Nous allons vous administrer l'alcool à jeun. Nous vous demandons donc de ne pas manger durant les trois heures qui précèdent votre arrivée. Durant les 24 heures qui précèdent chaque visite, nous vous demandons de ne pas consommer d'alcool, ni d'autres substance récréatives ayant un effet négatif sur votre conduite. Durant les quatre heures qui précèdent votre visite, nous vous demanderons de ne pas boire ni café, ni thé ou d'autres boissons contenant de la caféine ou de la théine (boissons énergisante, Coca Cola, etc.). Durant les deux heures précédant votre visite, vous ne devez plus consommer de nicotine (cigarette, patch, etc).

7. Avantages pour les participants de recherche

En participant à l'étude, vous pourriez profiter du résultat des tests neuropsychologiques pour avoir une meilleure idée de votre capacité de conduite. Vous connaitrez également mieux les conséquences concrètes de votre consommation d'alcool sur votre conduite. Il se peut cependant que vous n'ayez aucun avantage direct de participer à cette étude.

8. Risques et désagréments

Consommation d'alcool

La consommation alcool n'est pas exempté de risque et provoque des effets connus de sensation de perte d'équilibre, de troubles visuels, voir de nausées et de vomissements. La conduite sur simulateur peut augmenter ces symptômes d'intoxication. Les troubles neurologiques provoqués par la consommation d'alcool peuvent être responsable de chutes ou d'accidents. La consommation d'alcool chez la femme enceinte peut nuire au développement du fœtus.

MedDrive

D'après nos observations réalisées sur plus de 200 conducteurs, MedDrive ne provoque pas d'effet secondaire. Il n'est cependant pas exclu que ces exercices puissent provoqué des maux de tête ou une sensation de fatigue oculaire temporaire.

Simulateur de conduite

La conduite sur simulateur peut provoquer chez certaines personnes des nausées voir même des vomissements. Ceux-ci se manifestent d'avantage chez des personnes souffrant de maladie du voyage. Ces symptômes sont temporaires et n'ont aucune répercussion sur la santé à moyen ou à long terme.

9. Confidentialité des données

Les données personnelles que vous nous donnez sont rendues anonymes et ne sont ensuite accessibles qu'aux chercheurs universitaires à des fins d'analyses scientifiques. Les résultats de l'étude peuvent faire l'objet de publications qui seront rendues publiques. Les spécialistes compétents de la Commission d'éthique pourront, dans le cadre de ce que l'on appelle un audit, contrôler la procédure de réalisation de l'étude et, à ce titre, consulter les données vous concernant qui restent cependant anonymes. Votre confidentialité est cependant strictement garantie. Votre nom ne pourra donc en aucun cas être publié dans des rapports ou des publications qui découleraient de cette étude.

10. Frais

Cette étude ne vous occasionne aucun frais hormis ceux liés à vos déplacements en transport public pour vous rendre au Centre Médical Universitaire et de retour chez vous.. Les autres frais sont entièrement pris en charge par notre département.

11. Rémunération des participants volontaires

La visite permettant de vérifier si vous répondez aux critères de l'étude ne donne droit à aucune rémunération. Le fait de réaliser cinq fois à domicile les tests de MedDrive vous donne droit à CHF 100.-. Chaque visite ultérieure vous donne à nouveau droit à ce montant. Au total chaque participant ayant terminé l'étude sera donc rémunéré de CHF 500.-

12. Couverture des risques

En cas de dommages subis dans le cadre de l'étude, vous bénéficierez d'une compensation pleine et entière; une assurance spéciale a été contractée pour couvrir cette responsabilité. Le cas échéant l'investigateur vous prêtera assistance pour entreprendre les démarches nécessaires.

13. Interlocuteur(s)

En cas d'interrogations, d'incertitudes, d'empêchements, ou de remarques concernant l'étude, vous pouvez vous adresser durant les heures de bureau à l'investigateur ou au co-investigateur. En dehors de ces horaires, laissez un message au +41 78 788 33 66 et vous serez contacté le plus rapidement possible.

Rendez-vous
ISABELLE **CARDOSO**
☎ 079 724 46 84

Questions concernant l'étude
Paul **VAUCHER**
Etude OH-MedDrive
Doctorant en neuroscience
Université de Genève
☎ 078 788 33 66 (Swisscom)

Problèmes d'ordre médical en lien avec l'étude
DR BERNARD **FAVRAT**
Responsable de l'Unité de Médecine et de Psychologie du Trafic
☎ 079 556 61 83

Directeur de l'institution
Professeur PATRICE **MANGIN**
☎ 021 314 70 70

14. Vos obligations

En participant à cette étude, vous vous engagez à suivre les directives qui vous sont données, à maintenir une relation avec le personnel de recherche basée sur la confiance et l'honnêteté, et de rapporter tous événements indésirables survenant au court de l'étude.

A ne pas oublier pour le jour de l'entretien

- Nous vous serions reconnaissants d'apporter une **liste complète des médicaments** que vous prenez régulièrement ou occasionnellement.
- Vos **corrections optiques** que vous utilisez pour conduire.
- Votre **permis de conduire**.

Prof. Patrice Mangin
Directeur du Centre Universitaire
Médicale de Médecine Légale

Dr Bernard Favrat
Investigateur principal
Responsable de l'Unité de Médecine et de Psychologie du Trafic

Paul Vaucher
Co-investigateur
Doctorant

PROFIL PSYCHOLOGIQUE

Nous allons vous poser toute une série de questions qui visent à nous permettre de vous décrire. Ces questionnaires permettent donc de quantifier votre profil psychologique pour des aspects qui peuvent influencer votre manière de conduire.

Impulsivité (UPPS-P)

Vous trouverez ci-dessous un certain nombre d'énoncés décrivant des manières de se comporter ou de penser. Pour chaque affirmation, veuillez indiquer à quel degré vous êtes d'accord ou non avec l'énoncé. Si vous êtes Tout à fait d'accord avec l'affirmation NOIRCISSEZ LE CERCLE de la première colonne, si vous êtes Plutôt d'accord NOIRCISSEZ LE CERCLE de la deuxième colonne, si vous êtes Plutôt en désaccord NOIRCISSEZ LE CERCLE de la troisième colonne et si vous êtes Tout à fait en désaccord NOIRCISSEZ LE CERCLE de la dernière colonne. Assurez-vous que vous avez indiqué votre accord ou désaccord pour chaque énoncé ci-dessous.

	Tout à fait d'accord	Plutôt d'accord	Plutôt en désaccord	Tout à fait en désaccord
**D'habitude je réfléchis soigneusement avant de faire quoi que ce soit.**	○ 0	○ 1	○ 2	○ 3
**Quand je suis vraiment enthousiate, j'ai tendance à ne pas penser aux conséquences de mes actions.**	○ 3	○ 2	○ 1	○ 0
**J'aime parfois faire des choses qui sont un petit peu effrayantes.**	○ 3	○ 2	○ 1	○ 0
**Quand je suis contrarié(e), j'agis souvent sans réfléchir.**	○ 3	○ 2	○ 1	○ 0
**Je préfère généralement mener les choses jusqu'au bout.**	○ 0	○ 1	○ 2	○ 3
**Ma manière de penser est d'habitude réfléchie et méticuleuse.**	○ 0	○ 1	○ 2	○ 3
**Quand la discussion s'échauffe, je dis souvent des choses que je regrette ensuite.**	○ 3	○ 2	○ 1	○ 0
**J'achève ce que je commence.**	○ 0	○ 1	○ 2	○ 3
**J'éprouve du plaisir à prendre des risques.**	○ 3	○ 2	○ 1	○ 0
**Quand je suis ravi(e), je ne peux pas m'empêcher de m'emballer.**	○ 3	○ 2	○ 1	○ 0
**Une fois que je commence un projet, je le termine presque toujours.**	○ 0	○ 1	○ 2	○ 3
**J'aggrave souvent les choses parce que j'agis sans réfléchir quand je suis contrarié(e).**	○ 3	○ 2	○ 1	○ 0
**D'habitude je me décide après un raisonnement bien mûri.**	○ 0	○ 1	○ 2	○ 3
**Je recherche généralement des expériences et sensations nouvelles et excitantes.**	○ 3	○ 2	○ 1	○ 0
**Quand je suis vraiment enthousiaste, j'agis souvent sans réfléchir.**	○ 3	○ 2	○ 1	○ 0
**Je suis une personne productive qui termine toujours son travail.**	○ 0	○ 1	○ 2	○ 3
**Quand je me sens rejeté(e), je dis souvent de choses que je regrette par la suite.**	○ 3	○ 2	○ 1	○ 0
**Je me réjouis des expériences et sensations nouvelles même si elles sont un peu effrayantes et non-conformistes.**	○ 3	○ 2	○ 1	○ 0
**Avant de me décider, je considère tous les avantages et inconvénients.**	○ 0	○ 1	○ 2	○ 3
**Quand je suis très heureux/heureuse, j'ai l'impression qu'il est normal de céder à ses envies ou de se laisser aller à des excès.**	○ 3	○ 2	○ 1	○ 0

Urgence négative (gris gras) ____ / 12
Urgence positive (gris fin) ____ / 12
Manque de persévérance (noir gras) ____ / 12
Manque de préméditation (noir fin) ____ / 12
Recherche de sensation (trait tiret) ____ / 12

***SCORE UPPS-P** (total)* ____ / **60**

Billieux J, Rochat L, Ceschi G, et al. Validation of a short French version of the UPPS-P Impulsive Behavior Scale. Compr Psychiatry. Jul 2012;53(5):609-615

Agressivité (BPAQ-SF)

Lisez chaque énoncé et NOIRCISSEZ LE CERCLE APPROPRIE situé à droite de l'affirmation. Répondez vite et honnêtement.

	Ne me correspond pas du tout	Ne me correspond pas vraiment	Me correspond un peu	Me correspond bien	Me correspond parfaitement
**Si je suis suffisamment provoqué(e), je pourrais frapper quelqu'un**	○ 0	○ 1	○ 2	○ 3	○ 4
**Il m'arrive souvent d'être en désaccord avec les gens**	○ 0	○ 1	○ 2	○ 3	○ 4
**Parfois j'ai l'impression d'être poursuivi par la poisse**	○ 0	○ 1	○ 2	○ 3	○ 4
**Certaines personnes m'ont tellement poussé(e) à bout, que nous en sommes venu(e)s aux mains**	○ 0	○ 1	○ 2	○ 3	○ 4
**Lorsqu'on n'est pas d'accord avec moi, je ne peux pas m'empêcher de me disputer**	○ 0	○ 1	○ 2	○ 3	○ 4
**Il m'arrive de sortir de mes gongs sans raison valable**	○ 0	○ 1	○ 2	○ 3	○ 4
**Ce sont toujours les autres qui semblent avoir de la veine**	○ 0	○ 1	○ 2	○ 3	○ 4
**Il m'arrive de menacer des connaissances**	○ 0	○ 1	○ 2	○ 3	○ 4
**Mes amis disent que je cherche facillement la dispute**	○ 0	○ 1	○ 2	○ 3	○ 4
**J'ai de la peine à maîtriser ma colère**	○ 0	○ 1	○ 2	○ 3	○ 4
**Je me demande pourquoi je ressens parfois autant d'amertume**	○ 0	○ 1	○ 2	○ 3	○ 4
**Il m'arrive de me sentir comme une marmite à vapeur sur le point d'exploser**	○ 0	○ 1	○ 2	○ 3	○ 4

Score d'agressivité physique (noir gras) ____ / 16 *Score d'hostilité (gris gras)* ____ / 12

Score d'agressivité verbale (noir fin) ____ / 12 *Score de colère (gris fin)* ____ / 8

SCORE D'AGRESSIVITE** (total)* ____ ***/ 48

Diamond, P. M., & Magaletta, P. R. (2006). The short-form Buss-Perry Aggression Questionnaire (BPAQ-SF): a validation study with federal offenders. Assessment, 13(3), 227-240

Latéralité

Veuillez indiquez votre préférence de main ou de jambe pour les activités suivantes en NOIRCISSANT LE CERCLE APPROPRIE. Si vous n'êtes pas certain de la réponse, mimez ou imaginez vous en train de réaliser l'action en question.

Quelle(s) **main(s)** utiliseriez-vous	Toujours la/le **gauche**	Le plus souvent la/le **gauche**	Autant la/le **gauche** que la/le **droit(e)**	Le plus souvent la/le **droit(e)**	Toujours la/le **droit(e)**
**Pour frotter une allumette contre sa boîte ?**	○ -2	○ -1	○ 0	○ 1	○ 2
**Pour tenir votre brosse à dent ?**	○ -2	○ -1	○ 0	○ 1	○ 2
**Pour lancer une balle ?**	○ -2	○ -1	○ 0	○ 1	○ 2
**Pour tenir un marteau en enfonçant un clou ?**	○ -2	○ -1	○ 0	○ 1	○ 2
**Pour jouer avec une raquette d'une seule main ?**	○ -2	○ -1	○ 0	○ 1	○ 2
**Pour écrire ?**	○ -2	○ -1	○ 0	○ 1	○ 2
Quel(s) **pied(s)** utiliseriez-vous					
**Pour frapper une balle immobile en direction d'une cible droit devant vous ?**	○ -2	○ -1	○ 0	○ 1	○ 2
**Pour vous tenir sur une seule jambe ?**	○ -2	○ -1	○ 0	○ 1	○ 2
**Pour aplatir du sable sur la plage ?**	○ -2	○ -1	○ 0	○ 1	○ 2
**En premier en montant sur une chaise ?**	○ -2	○ -1	○ 0	○ 1	○ 2
**Pour écraser un cafard se déplaçant rapidement sur le sol ?**	○ -2	○ -1	○ 0	○ 1	○ 2
**Pour vous maintenir en équilibre sur un seul pied sur une poutre ?**	○ -2	○ -1	○ 0	○ 1	○ 2
**Pour saisir une bille posée sur le sol avec les orteils ?**	○ -2	○ -1	○ 0	○ 1	○ 2
**Pour sautiller sur un seul pied ?**	○ -2	○ -1	○ 0	○ 1	○ 2
**Pour enfoncer une bêche dans la terre ?**	○ -2	○ -1	○ 0	○ 1	○ 2
**Comme apuis lorsque vous vous stationnez debout, une jambe légèrement pliée ?**	○ -2	○ -1	○ 0	○ 1	○ 2

Latéralisation du membre supérieur (noir fin) ____ / 12

Latéralisation de soutient du membre inférieur (noir gras) ____ / 10

Latéralisation de maniement du membre inférieur (gris gras) ____ / 10

SCORE GLOBAL DE LATERALISATION** (total)* ____ ***/ 32

Annett M. A classification of hand preference by association analysis. Br J Psychol. Aug 1970;61(3):303-321;

Elias LJ, Bryden MP, Bulman-Fleming MB. Footedness is a better predictor than is handedness of emotional lateralization. Neuropsychologia. Jan 1998;36(1):37-43

PSYCHOTROPES

Les prochaines questions visent à évaluer votre consommation de substances pouvant influencer votre conduite. Ceci nous permet de discuter des éventuelles difficultés que vous pourrez rencontrer a ne pas consommer de substances pour l'étude.

Médicaments (AFSSAPS)

Veuillez-nous indiquer le nom des médicaments que vous prennez régulièrement ou occasionnellement pendant les trois derniers mois. (SI = présentement sous l'influence du médicament)

Substance active	Classe	SI	Substance active	Classe	ST
____________	_____	☐	____________	_____	☐
____________	_____	☐	____________	_____	☐
____________	_____	☐	____________	_____	☐
____________	_____	☐	____________	_____	☐

Classification établie par: Riche C, Caulin C, Caron J, et al. Médicaments et conduite automobile. Paris: Agence française de sécurité sanitaire des produits de santé (afssaps);2009

Contraceptif (ne concerne que les femmes)

Test de grossesse Negatif ☐ 0 Positif ☐ 1

Critère d'exclusion

Moyen contraceptif actuel Preservatif ☐ 1 Contraceptif oral ou sous-cutané ☐ 2 Sterilet ☐ 3 Autre ☐ 4

Critère d'exclusion

Tabagisme (FTND)

Avez-vous consommé du tabac durant les six derniers mois ? Oui ☐ 1 Non ☐ 0 → *Passez à la section suivante*

Le matin, combien de temps après vous être réveillé fumez-vous votre première cigarette ? Dans les 5 minutes ☐ 3 6-30 minutes ☐ 2 31-60 minutes ☐ 1 Plus de 60 minutes ☐ 0

Trouvez-vous qu'il est difficile de vous abstenir de fumer dans des endroits où c'est interdit ? (p.ex. cinémas, bibliothèques) Oui ☐ 1 Non ☐ 0

A quelle cigarette renonceriez-vous le plus difficilement ? A la première de la journée ☐ 1 A une autre ☐ 0

Combien de cigarette fumez-vous par jour, en moyenne? 10 ou moins ☐ 0 11-20 ☐ 1 21-30 ☐ 2 31 ou plus ☐ 3

Fumez-vous à intervalles plus rapprochés durant les premières heures de la matinée que durant le reste de la journée ? Oui ☐ 1 Non ☐ 0

Fumez-vous lorsque vous êtes malade au point de devoir rester au lit presque toute la journée ? Oui ☐ 1 Non ☐ 0

Heatherton TF, Kozlowski LT, Frecker RC, Fagerstrom KO. The Fagerstrom Test for Nicotine Dependence: a revision of the Fagerstrom Tolerance Questionnaire. British journal of addiction. Sep 1991;86(9):1119-112
Etter JF, Duc TV, Perneger TV. Validity of the Fagerstrom test for nicotine dependence and of the Heaviness of Smoking Index among relatively light smokers. Addiction. Feb 1999;94(2):269-281

Consommation d'alcool (AUDIT-C)

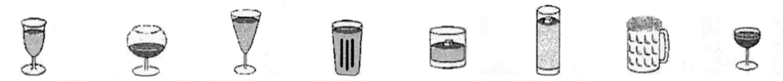

7cl d'apéritif à 18° | 2,5 cl de digestif à 45° | 10 cl de champagne à12° | 25 cl de cidre à 5° | 2,5 cl de wisky à 45° | 2,5 cl de pastis à 45° | 25 cl de bière à 5° | 10 cl de vin à 12°

Les verres ci-dessus contiennent tous la même quantité d'alcool pur (verre standard). Attention: une cannette de 50cl de bière forte (8.6° ou 10°) contient l'équivalent de 4 verres standards et une bouteille de vin contient 8 verres standards.

À quelle fréquence vous arrive-t-il de consommer des boissons contenant de l'alcool ?

Jamais ☐ 0 1 fois par mois ou moins ☐ 1 2 à 4 fois par mois ☐ 2 2 à 3 fois par semaine ☐ 3 4 fois ou plus par semaine ☐ 4

Combien de verres standards buvez-vous au cours d'une journée ordinaire où vous buvez de l'alcool ?

1 ou 2 ☐ 0 3 ou 4 ☐ 1 5 ou 6 ☐ 2 7 à 9 ☐ 3 10 ou plus ☐ 4

Au cours d'une même occasion, à quelle fréquence vous arrive-t-il de boire six verres standards ou plus ?

Jamais ☐ 0 Moins d'une fois par mois ☐ 1 Une fois par mois ☐ 2 Une fois par semaine ☐ 3 Chaque jour ou presque ☐ 4

Bradley KA, DeBenedetti AF, Volk RJ, Williams EC, Frank D, Kivlahan DR. AUDIT-C as a brief screen for alcohol misuse in primary care. Alcohol Clin Exp Res. Jul 2007;31(7):1208-1217.

Autres substances récréatives (DAST-10)

Les questions qui suivent concernent votre consommation de drogue autre que l'alcool et le tabac durant les 12 mois précédants. L'abus de drogue se réfère donc à (1) une consommation de médicaments prescrits ou acheté en vente libre en excès par rapport aux recommandations, (2) toute consommation de drogues non-médicale tel que cannabis, cocaïne, LSD, ecstasy, etc.. Lisez chaque énnoncé et répondez le plus honnêtement possible par "oui" ou "non" en NOIRCISSANT le cercle correspondant à votre réponse.

	oui	***non***
**Avez-vous consommé une drogue pour une raison autre que médicale ?**	○ 1	○ 0
Si vous avez répondu "oui" à cette première question, répondez aux questions suivantes, sinon, passez à la page suivante		
**Avez-vous été sous l'influence de plus d'une drogue à la fois ?**	○ 1	○ 0
**Arrivez-vous à arrêter votre consommation de drogue quand vous voulez ?**	○ 0	○ 1
**Vous êtes-vous évanoui(e) ou eu un flash-back lié à votre consommation de drogue ?**	○ 1	○ 0
**Vous est-il arrivé(e) de vous sentir mal ou coupable de votre consommation de drogue ?**	○ 1	○ 0
**Un membre de votre famille vous a-t-il fait une remarque sur votre consommation de drogue ?**	○ 1	○ 0
**Votre consommation de drogue vous a-t-elle éloignée de votre famille ?**	○ 1	○ 0
**Avez-vous entrepris une activité illégale pour obtenir de la drogue ?**	○ 1	○ 0
**Avez-vous déjà senti des signes de sevrage (se sentir malade) en arrêtant votre consommation de drogue ?**	○ 1	○ 0
**Avez-vous eu des soucis médicaux liés à votre consommation de drogue (exemple: perte de mémoire, hépatite, convulsions, saignements) ?**	○ 1	○ 0

≥ 3 investiguer abus de substance

Yudko E, Lozhkina O, Fouts A. A comprehensive review of the psychometric properties of the Drug Abuse Screening Test. Journal of substance abuse treatment. Mar 2007;32(2):189-198

SOCIO-DEMOGRAPHIQUES

Genre Homme ☐ 1 Femme ☐ 0 **Année de naissance** ______ **Nbr d'années de scolarité** ____ans

Historique de conduite

Année d'obtention du premier permis A (moto) ou B (voiture) _ _ _ _
Année d'obtention du permis voiture _ _ _ _

Durant les deux dernières années, quel type de véhicule avez-vous conduit au moins une fois? Vélo ☐ 0/1 Vélo-moteur ☐ 0/1 Véhicule agricole ☐ 0/1 Moto < 125cm³ ☐ 0/1 Moto ≤ 25 kW ☐ 0/1 Moto > 25 kW ☐ 0/1 Voiture ☐ 0/1 Remorque ☐ 0/1 Camion ☐ 0/1 Car ☐ 0/1

Quelle est la distance moyenne que vous conduisez par semaine ? ________km

A quand remonte votre dernier accident, touchette ou autre, fautif(ve) ou pas, avec ou sans implication de tiers ? ____ . ____ . ______

Souffrez-vous suffisamment d'un des troubles suivants pour que cela empiète sur la qualité de votre conduite ? Trouble moteur ☐ 0/1 Trouble visuel ☐ 0/1 Trouble cognitif ☐ 0/1 Trouble de l'humeur ☐ 0/1 Psychose ☐ 0/1 Fatigue ☐ 0/1 Epilepsie ☐ 0/1 Diabète ☐ 0/1 Trouble cardiaque ☐ 0/1

ENTRAINEMENT

Nous allons maintenant passé aux questions et épreuves vous permettant de vous familiariser avec celles que vous aller effectuer lors de vos quatre prochaines visites.

Substances psychogènes

Pour chaque substance ci-dessous, veuillez préciser votre consommation durant les 48h passées. Pour le déroulement de l'étude, il est important de nous répondre correctement.

Consommation 48h

Alcool Non ☐ Oui ☐ → nbr d'heures écoulées ___ h → nbr de verres à cette occasion ___ verres

Tabac Non ☐ Oui ☐ → nbr d'heures écoulées ___ h → nbr de cigarettes durant 48h ___ cig.

Café/Thé Non ☐ Oui ☐ → nbr d'heures écoulées ___ h → nbr de tasses durant 48h ___ tasses

Energy drink Non ☐ Oui ☐ → nbr d'heures écoulées ___ h → nbr de doses durant 48h ___ doses

Consommation 48h

Médicaments Non ☐ Oui ☐ → ____________________

Cannabis Non ☐ Oui ☐ → nbr d'heures écoulées ____ h

Autres Non ☐ Oui ☐ → ____________________ → nbr d'heures écoulées ____ h

TMT

TMT-A________sec TMT-B________sec

Vision

Acuité Droite : _______ Gauche : _______ Bilat. : _______

Champs visuel Droite : _______ N ___ Gauche : _______ N ___

Sensibilité au contraste (MARS) Droite : _______ Gauche : _______ Bilat. : _______

Epreuves sur ordinateur

Date: ____ . ____ . ________ Heure ____:____

UFOV-7
Procedural ________ms
Divided ________ms
Selective ________ms

MedDrive (T1-T4) ☐
Simulateur (S1-S3) ☐

Mal de voyage (SSQ-F)

NOIRCISSEZ LE CERCLE correspondant à l'intensité du symptôme ressenti pendant et après l'utilisation du simulateur.

	Pas du tout	Un peu	Modérément	Sévèrement
**Inconfort générale**	○ 0	○ 1	○ 2	○ 3
**Fatigue**	○ 0	○ 1	○ 2	○ 3
**Mal de tête**	○ 0	○ 1	○ 2	○ 3
**Fatigue des yeux**	○ 0	○ 1	○ 2	○ 3
**Difficulté à faire le focus**	○ 0	○ 1	○ 2	○ 3
**Augmentation de la salivation**	○ 0	○ 1	○ 2	○ 3
**Transpiration**	○ 0	○ 1	○ 2	○ 3
**Nausées**	○ 0	○ 1	○ 2	○ 3
**Difficulté à se concentrer**	○ 0	○ 1	○ 2	○ 3
**Impression de lourdeur dans la tête**	○ 0	○ 1	○ 2	○ 3
**Vision embrouillée**	○ 0	○ 1	○ 2	○ 3
**Etourdissement les yeux ouverts**	○ 0	○ 1	○ 2	○ 3
**Etourdisement les yeux fermés**	○ 0	○ 1	○ 2	○ 3
*** Vertiges**	○ 0	○ 1	○ 2	○ 3
**** Conscience de l'etomac**	○ 0	○ 1	○ 2	○ 3
**Rots**	○ 0	○ 1	○ 2	○ 3

Score de nausées (noir) ____ / 27
Score oculo-moteur (gris) ____ / 21
***SCORE TOTAL** (UQO)* ____ / **48**

** Les vertiges sont vécus comme une perte de l'orientation par rapport à la position verticale.*
*** L'expression "conscience de l'estomac" est habituellement utilisée pour designer un sentiment d'inconfort sans naussée.*

Kennedy RS, Lane NE, Berbaum KS, Lilienthal MG. Simulator Sickness Questionnaire: An Enhanced Method for Quantifying Simulator Sickness. The International Journal of Aviation Psychology. 1993/07/01 1993;3(3):203-220
Bouchard S, Robillard G, Renaud P, Bernier F. Exploring New Dimensions in the Assessment of Virtual Reality Induced Side Effects. J Comput & Inform Tech. 2011;1(3):20-32

Evènements indésirables

Nous allons vous interroger sur votre état de santé durant la semaine écoulée. Il est important de nous rapporter tout nouveaux signes (exemple: mal de tête, fièvre, sels liquides) que vous n'aviez pas avant de participer à cette expérience.

**Durant les 48h qui ont suivi votre visite précédante, avez vous ressenti :**	Non	Oui
des maux de tête	☐	☐
de la fatigue occulaire	☐	☐
de la difficulté à vous endormir	☐	☐
de la difficulté à vous concentrer	☐	☐

**Durant la semaine écoulée avez-vous eu :**	Non	Oui
un accident (chute, trauma, etc.)	☐	☐
de la fièvre	☐	☐
l'apparition d'un autre signe inhabituel	☐	☐
un rapport non-protégé (femme uniquement)	☐	☐

Si vous avez répondu "oui" à une des questions ci-dessus, veuillez détailler la nature de ces signes. Pour chacun d'entre eux, l'investigateur va déterminer la sévérité (S ; 0=aucune intervention nécessaire, 1=à nécessité une intervention médicale, 2=à nécessité hospitalisation, 3= à mis votre vie en danger, 4=à entrainé votre mort) et le lien avec l'utilisation de MedDrive ou l'administration d'alcool (I; .=indéterminée, 0=exclue, 1=douteuse, 2=possible, 3=probable, 4=certaine)

______________________________ ______________________________
______________________________ ______________________________
______________________________ ______________________________

Substances psychogènes

Pour chaque substance ci-dessous, veuillez préciser votre consommation durant les 48h passées. Pour le déroulement de l'étude, il est important de nous répondre correctement.

Consommation 48h

Alcool Non ☐ Oui ☐ → nbr d'heures écoulées ___ h → nbr de verres à cette occasion ___ verres

Tabac Non ☐ Oui ☐ → nbr d'heures écoulées ___ h → nbr de cigarettes durant 48h ___ cig.

Café/Thé Non ☐ Oui ☐ → nbr d'heures écoulées ___ h → nbr de tasses durant 48h ___ tasses

Energy drink Non ☐ Oui ☐ → nbr d'heures écoulées ___ h → nbr de doses durant 48h ___ doses

Consommation 48h

Médicaments Non ☐ Oui ☐ → ____________________

Cannabis Non ☐ Oui ☐ → nbr d'heures écoulées _____ h

Autres Non ☐ Oui ☐ → ____________________ → nbr d'heures écoulées _____ h

Vision

Sensibilité au contraste (MARS) Droite : _______ Gauche : _______ Bilat. : _______

TMT

TMT-A_________sec TMT-B_________sec

Epreuves sur ordinateur

Date: ____ . ____ . ________ heure ____:____

UFOV-7

Procedural _________ms
Divided _________ms
Selective _________ms

MedDrive T1 ☐
MedDrive T1bis ☐
MedDrive T2-T4 ☐
Simulateur S1-S3 ☐

Mal de voyage (SSQ-F)

NOIRCISSEZ LE CERCLE correspondant à l'intensité du symptôme ressenti pendant et après l'utilisation du simulateur.

	Pas du tout	Un peu	Modérément	Sévèrement
**Inconfort générale**	○ 0	○ 1	○ 2	○ 3
**Fatigue**	○ 0	○ 1	○ 2	○ 3
**Mal de tête**	○ 0	○ 1	○ 2	○ 3
**Fatigue des yeux**	○ 0	○ 1	○ 2	○ 3
**Difficulté à faire le focus**	○ 0	○ 1	○ 2	○ 3
**Augmentation de la salivation**	○ 0	○ 1	○ 2	○ 3
**Transpiration**	○ 0	○ 1	○ 2	○ 3
**Nausées**	○ 0	○ 1	○ 2	○ 3
**Difficulté à se concentrer**	○ 0	○ 1	○ 2	○ 3
**Impression de lourdeur dans la tête**	○ 0	○ 1	○ 2	○ 3
**Vision embrouillée**	○ 0	○ 1	○ 2	○ 3
**Etourdissement les yeux ouverts**	○ 0	○ 1	○ 2	○ 3
**Etourdisement les yeux fermés**	○ 0	○ 1	○ 2	○ 3
*** Vertiges**	○ 0	○ 1	○ 2	○ 3
**** Conscience de l'etomac**	○ 0	○ 1	○ 2	○ 3
**Rots**	○ 0	○ 1	○ 2	○ 3

Kennedy RS, Lane NE, Berbaum KS, Lilienthal MG. Simulator Sickness Questionnaire: An Enhanced Method for Quantifying Simulator Sickness. The International Journal of Aviation Psychology. 1993/07/01 1993;3(3):203-220
Bouchard S, Robillard G, Renaud P, Bernier F. Exploring New Dimensions in the Assessment of Virtual Reality Induced Side Effects. J Comput & Inform Tech. 2011;1(3):20-32

** Les vertiges sont vécus comme une perte de l'orientation par rapport à la position verticale.*
*** L'expression "conscience de l'estomac" est habituellement utilisée pour designer un sentiment d'inconfort sans naussée.*

Score de nausées (noir) ____ / 27
Score oculo-moteur (gris) ____ / 21
***SCORE TOTAL** (UQO)* ____ **/ 48**

Allocation

Votre boisson contenait-elle de l'alcool ? Non ☐ Oui ☐ → pour quelle alcoolémie ? 0.5‰ ☐ 0.43‰ ☐ . ,6‰ ☐

Parmis les points suivants, lesquels vous ont aidé(e) pour répondre à la question précédante ? (plusieurs réponses possibles)

le goût ☐ ma performance ☐ l'attitude de l'investigateur ☐ l'attitude de l'examinateur ☐ les effets (tête qui tourne, nausée) ☐

Evènements indésirables

Nous allons vous interroger sur votre état de santé durant la semaine écoulée. Il est important de nous rapporter tout nouveaux signes (exemple: mal de tête, fièvre, sels liquides) que vous n'aviez pas avant de participer à cette expérience.

**Durant les 48h qui ont suivi votre visite précédante, avez vous ressenti :**	Non	Oui
des maux de tête	☐	☐
de la fatigue occulaire	☐	☐
de la difficulté à vous endormir	☐	☐
de la difficulté à vous concentrer	☐	☐

**Durant la semaine écoulée avez-vous eu :**	Non	Oui
un accident (chute, trauma, etc.)	☐	☐
de la fièvre	☐	☐
l'apparition d'un autre signe inhabituel	☐	☐
un rapport non-protégé (femme uniquement)	☐	☐

Si vous avez répondu "oui" à une des questions ci-dessus, veuillez détailler la nature de ces signes. Pour chacun d'entre eux, l'investigateur va déterminer la sévérité (S ; 0=aucune intervention nécessaire, 1=à nécessité une intervention médicale, 2=à nécessité hospitalisation, 3= à mis votre vie en danger, 4=à entrainé votre mort) et le lien avec l'utilisation de MedDrive ou l'administration d'alcool (I; .=indéterminée, 0=exclue, 1=douteuse, 2=possible, 3=probable, 4=certaine)

______________________ ______________________

______________________ ______________________

______________________ ______________________

Substances psychogènes

Pour chaque substance ci-dessous, veuillez préciser votre consommation durant les 48h passées. Pour le déroulement de l'étude, il est important de nous répondre correctement.

Consommation 48h

- **Alcool** Non ☐ Oui ☐ → nbr d'heures écoulées ___ h; → nbr de verres à cette occasion ___ verres
- **Tabac** Non ☐ Oui ☐ → nbr d'heures écoulées ___ h; → nbr de cigarettes durant 48h ___ cig.
- **Café/Thé** Non ☐ Oui ☐ → nbr d'heures écoulées ___ h; → nbr de tasses durant 48h ___ tasses
- **Energy drink** Non ☐ Oui ☐ → nbr d'heures écoulées ___ h; → nbr de doses durant 48h ___ doses

Consommation 48h

- **Médicaments** Non ☐ Oui ☐ → ____________________
- **Cannabis** Non ☐ Oui ☐ → nbr d'heures écoulées _____ h
- **Autres** Non ☐ Oui ☐ → ____________________; → nbr d'heures écoulées _____ h

Vision

Sensibilité au contraste (MARS) Droite : _______ Gauche : _______ Bilat. : _______

TMT

TMT-A_________sec TMT-B_________sec

Epreuves sur ordinateur

Date: ____ . ____ . ________ heure ____:____

UFOV-7

Procedural _________ms
Divided _________ms
Selective _________ms

MedDrive T1 ☐
MedDrive T1bis ☐
MedDrive T2-T4 ☐
Simulateur S1-S3 ☐

Mal de voyage (SSQ-F)

NOIRCISSEZ LE CERCLE correspondant à l'intensité du symptôme ressenti pendant et après l'utilisation du simulateur.

	Pas du tout	Un peu	Modérément	Sévèrement
**Inconfort générale**	○ 0	○ 1	○ 2	○ 3
**Fatigue**	○ 0	○ 1	○ 2	○ 3
**Mal de tête**	○ 0	○ 1	○ 2	○ 3
**Fatigue des yeux**	○ 0	○ 1	○ 2	○ 3
**Difficulté à faire le focus**	○ 0	○ 1	○ 2	○ 3
**Augmentation de la salivation**	○ 0	○ 1	○ 2	○ 3
**Transpiration**	○ 0	○ 1	○ 2	○ 3
**Nausées**	○ 0	○ 1	○ 2	○ 3
**Difficulté à se concentrer**	○ 0	○ 1	○ 2	○ 3
**Impression de lourdeur dans la tête**	○ 0	○ 1	○ 2	○ 3
**Vision embrouillée**	○ 0	○ 1	○ 2	○ 3
**Etourdissement les yeux ouverts**	○ 0	○ 1	○ 2	○ 3
**Etourdisement les yeux fermés**	○ 0	○ 1	○ 2	○ 3
*** Vertiges**	○ 0	○ 1	○ 2	○ 3
**** Conscience de l'etomac**	○ 0	○ 1	○ 2	○ 3
**Rots**	○ 0	○ 1	○ 2	○ 3

** Les vertiges sont vécus comme une perte de l'orientation par rapport à la position verticale.*
*** L'expression "conscience de l'estomac" est habituellement utilisée pour designer un sentiment d'inconfort sans naussée.*

Score de nausées (noir) ____ / 27
Score oculo-moteur (gris) ____ / 21
***SCORE TOTAL** (UQO)* ____ **/ 48**

Kennedy RS, Lane NE, Berbaum KS, Lilienthal MG. Simulator Sickness Questionnaire: An Enhanced Method for Quantifying Simulator Sickness. The International Journal of Aviation Psychology. 1993/07/01 1993;3(3):203-220
Bouchard S, Robillard G, Renaud P, Bernier F. Exploring New Dimensions in the Assessment of Virtual Reality Induced Side Effects. J Comput & Inform Tech. 2011;1(3):20-32

Allocation

Votre boisson contenait-elle de l'alcool ? Non ☐ Oui ☐ → pour quelle alcoolémie ? 0.5‰ ☐ 0.8‰ ☐ 1.0‰ ☐

Parmis les points suivants, lesquels vous ont aidé(e) pour répondre à la question précédante ? (plusieurs réponses possibles)

le goût ☐ l'attitude de l'investigateur ☐ les effets (tête qui tourne, nausée) ☐
ma performance ☐ l'attitude de l'examinateur ☐ par déduction des cond. restantes ☐

Evènements indésirables

Nous allons vous interroger sur votre état de santé durant la semaine écoulée. Il est important de nous rapporter tout nouveaux signes (exemple: mal de tête, fièvre, sels liquides) que vous n'aviez pas avant de participer à cette expérience.

Durant les 48h qui ont suivi votre visite précédante, avez vous ressenti :

	Non	Oui
des maux de tête	☐	☐
de la fatigue occulaire	☐	☐
de la difficulté à vous endormir	☐	☐
de la difficulté à vous concentrer	☐	☐

Durant la semaine écoulée avez-vous eu :

	Non	Oui
un accident (chute, trauma, etc.)	☐	☐
de la fièvre	☐	☐
l'apparition d'un autre signe inhabituel	☐	☐
un rapport non-protégé (femme uniquement)	☐	☐

Si vous avez répondu "oui" à une des questions ci-dessus, veuillez détailler la nature de ces signes. Pour chacun d'entre eux, l'investigateur va déterminer la sévérité (S ; 0=aucune intervention nécessaire, 1=à nécessité une intervention médicale, 2=à nécessité hospitalisation, 3= à mis votre vie en danger, 4=à entrainé votre mort) et le lien avec l'utilisation de MedDrive ou l'administration d'alcool (I; .=indéterminée, 0=exclue, 1=douteuse, 2=possible, 3=probable, 4=certaine)

Substances psychogènes

Pour chaque substance ci-dessous, veuillez préciser votre consommation durant les 48h passées. Pour le déroulement de l'étude, il est important de nous répondre correctement.

Consommation 48h

Alcool Non ☐ Oui ☐ → nbr d'heures écoulées ___ h; nbr de verres à cette occasion ___ verres

Tabac Non ☐ Oui ☐ → nbr d'heures écoulées ___ h; nbr de cigarettes durant 48h ___ cig.

Café/Thé Non ☐ Oui ☐ → nbr d'heures écoulées ___ h; nbr de tasses durant 48h ___ tasses

Energy drink Non ☐ Oui ☐ → nbr d'heures écoulées ___ h; nbr de doses durant 48h ___ doses

Consommation 48h

Médicaments Non ☐ Oui ☐ → ____________

Cannabis Non ☐ Oui ☐ → nbr d'heures écoulées _____ h

Autres Non ☐ Oui ☐ → ____________; nbr d'heures écoulées _____ h

Vision

Sensibilité au contraste (MARS) Droite : _______ Gauche : _______ Bilat. : _______

TMT

TMT-A_________sec TMT-B_________sec

Epreuves sur ordinateur

Date: ____ . ____ . ________ heure ____:____

UFOV-7

Procedural _________ms
Divided _________ms
Selective _________ms

MedDrive T1 ☐
MedDrive T1bis ☐
MedDrive T2-T4 ☐
Simulateur S1-S3 ☐

Mal de voyage (SSQ-F)

NOIRCISSEZ LE CERCLE correspondant à l'intensité du symptôme ressenti pendant et après l'utilisation du simulateur.

	Pas du tout	Un peu	Modérément	Sévèrement
**Inconfort générale**	○ 0	○ 1	○ 2	○ 3
**Fatigue**	○ 0	○ 1	○ 2	○ 3
**Mal de tête**	○ 0	○ 1	○ 2	○ 3
**Fatigue des yeux**	○ 0	○ 1	○ 2	○ 3
**Difficulté à faire le focus**	○ 0	○ 1	○ 2	○ 3
**Augmentation de la salivation**	○ 0	○ 1	○ 2	○ 3
**Transpiration**	○ 0	○ 1	○ 2	○ 3
**Nausées**	○ 0	○ 1	○ 2	○ 3
**Difficulté à se concentrer**	○ 0	○ 1	○ 2	○ 3
**Impression de lourdeur dans la tête**	○ 0	○ 1	○ 2	○ 3
**Vision embrouillée**	○ 0	○ 1	○ 2	○ 3
**Etourdissement les yeux ouverts**	○ 0	○ 1	○ 2	○ 3
**Etourdisement les yeux fermés**	○ 0	○ 1	○ 2	○ 3
*** Vertiges**	○ 0	○ 1	○ 2	○ 3
**** Conscience de l'etomac**	○ 0	○ 1	○ 2	○ 3
**Rots**	○ 0	○ 1	○ 2	○ 3

Kennedy RS, Lane NE, Berbaum KS, Lilienthal MG. Simulator Sickness Questionnaire: An Enhanced Method for Quantifying Simulator Sickness. The International Journal of Aviation Psychology. 1993/07/01 1993;3(3):203-220
Bouchard S, Robillard G, Renaud P, Bernier F. Exploring New Dimensions in the Assessment of Virtual Reality Induced Side Effects. J Comput & Inform Tech. 2011;1(3):20-32

** Les vertiges sont vécus comme une perte de l'orientation par rapport à la position verticale.*
*** L'expression "conscience de l'estomac" est habituellement utilisée pour designer un sentiment d'inconfort sans naussée.*

Score de nausées (noir) ____ / 27
Score oculo-moteur (gris) ____ / 21
***SCORE TOTAL** (UQO)* ____ / **48**

Allocation

Votre boisson contenait-elle de l'alcool ? Non ☐ Oui ☐ → pour quelle alcoolémie ? 0.5‰ ☐ 0.8‰ ☐ 1.0‰ ☐

Parmis les points suivants, lesquels vous ont aidé(e) pour répondre à la question précédante ? (plusieurs réponses possibles)

le goût ☐ l'attitude de l'investigateur ☐ les effets (tête qui tourne, nausée) ☐
ma performance ☐ l'attitude de l'examinateur ☐ par déduction des cond. restantes ☐

Evènements indésirables

Nous allons vous interroger sur votre état de santé durant la semaine écoulée. Il est important de nous rapporter tout nouveaux signes (exemple: mal de tête, fièvre, sels liquides) que vous n'aviez pas avant de participer à cette expérience.

**Durant les 48h qui ont suivi votre visite précédante, avez vous ressenti :**	Non	Oui
des maux de tête	☐	☐
de la fatigue occulaire	☐	☐
de la difficulté à vous endormir	☐	☐
de la difficulté à vous concentrer	☐	☐

**Durant la semaine écoulée avez-vous eu :**	Non	Oui
un accident (chute, trauma, etc.)	☐	☐
de la fièvre	☐	☐
l'apparition d'un autre signe inhabituel	☐	☐
un rapport non-protégé (femme uniquement)	☐	☐

Si vous avez répondu "oui" à une des questions ci-dessus, veuillez détailler la nature de ces signes. Pour chacun d'entre eux, l'investigateur va déterminer la sévérité (S ; 0=aucune intervention nécessaire, 1=à nécessité une intervention médicale, 2=à nécessité hospitalisation, 3= à mis votre vie en danger, 4=à entrainé votre mort) et le lien avec l'utilisation de MedDrive ou l'administration d'alcool (I; .=indéterminée, 0=exclue, 1=douteuse, 2=possible, 3=probable, 4=certaine)

_______________ _______________

_______________ _______________

_______________ _______________

Substances psychogènes

Pour chaque substance ci-dessous, veuillez préciser votre consommation durant les 48h passées. Pour le déroulement de l'étude, il est important de nous répondre correctement.

Consommation 48h

Alcool Non ☐ Oui ☐ → nbr d'heures écoulées ___ h → nbr de verres à cette occasion ___ verres

Tabac Non ☐ Oui ☐ → nbr d'heures écoulées ___ h → nbr de cigarettes durant 48h ___ cig.

Café/Thé Non ☐ Oui ☐ → nbr d'heures écoulées ___ h → nbr de tasses durant 48h ___ tasses

Energy drink Non ☐ Oui ☐ → nbr d'heures écoulées ___ h → nbr de doses durant 48h ___ doses

Consommation 48h

Médicaments Non ☐ Oui ☐ → ____________

Cannabis Non ☐ Oui ☐ → nbr d'heures écoulées _____ h

Autres Non ☐ Oui ☐ → ____________ → nbr d'heures écoulées _____ h

Vision

Sensibilité au contraste (MARS) Droite : _______ Gauche : _______ Bilat. : _______

TMT

TMT-A_________sec TMT-B_________sec

Epreuves sur ordinateur

Date: ____ . ____ . ________ heure ____:____

UFOV-7

Procedural _________ms
Divided _________ms
Selective _________ms

MedDrive T1 ☐
MedDrive T1bis ☐
MedDrive T2-T4 ☐
Simulateur S1-S3 ☐

Mal de voyage (SSQ-F)

NOIRCISSEZ LE CERCLE correspondant à l'intensité du symptôme ressenti pendant et après l'utilisation du simulateur.

	Pas du tout	Un peu	Modérément	Sévèrement
**Inconfort générale**	○ 0	○ 1	○ 2	○ 3
**Fatigue**	○ 0	○ 1	○ 2	○ 3
**Mal de tête**	○ 0	○ 1	○ 2	○ 3
**Fatigue des yeux**	○ 0	○ 1	○ 2	○ 3
**Difficulté à faire le focus**	○ 0	○ 1	○ 2	○ 3
**Augmentation de la salivation**	○ 0	○ 1	○ 2	○ 3
**Transpiration**	○ 0	○ 1	○ 2	○ 3
**Nausées**	○ 0	○ 1	○ 2	○ 3
**Difficulté à se concentrer**	○ 0	○ 1	○ 2	○ 3
**Impression de lourdeur dans la tête**	○ 0	○ 1	○ 2	○ 3
**Vision embrouillée**	○ 0	○ 1	○ 2	○ 3
**Etourdissement les yeux ouverts**	○ 0	○ 1	○ 2	○ 3
**Etourdisement les yeux fermés**	○ 0	○ 1	○ 2	○ 3
*** Vertiges**	○ 0	○ 1	○ 2	○ 3
**** Conscience de l'etomac**	○ 0	○ 1	○ 2	○ 3
**Rots**	○ 0	○ 1	○ 2	○ 3

** Les vertiges sont vécus comme une perte de l'orientation par rapport à la position verticale.*
*** L'expression "conscience de l'estomac" est habituellement utilisée pour designer un sentiment d'inconfort sans naussée.*

Score de nausées (noir) _____ / 27
Score oculo-moteur (gris) _____ / 21
***SCORE TOTAL** (UQO)* _____ **/ 48**

Kennedy RS, Lane NE, Berbaum KS, Lilienthal MG. Simulator Sickness Questionnaire: An Enhanced Method for Quantifying Simulator Sickness. The International Journal of Aviation Psychology. 1993/07/01 1993;3(3):203-220
Bouchard S, Robillard G, Renaud P, Bernier F. Exploring New Dimensions in the Assessment of Virtual Reality Induced Side Effects. J Comput & Inform Tech. 2011;1(3):20-32

Allocation

Votre boisson contenait-elle de l'alcool ? Non ☐ Oui ☐ → pour quelle alcoolémie ? 0.5‰ ☐ 0.8‰ ☐ 1.0‰ ☐

Parmis les points suivants, lesquels vous ont aidé(e) pour répondre à la question précédante ? (plusieurs réponses possibles)

le goût ☐ l'attitude de l'investigateur ☐ les effets (tête qui tourne, nausée) ☐
ma performance ☐ l'attitude de l'examinateur ☐ par déduction des cond. restantes ☐

EVENEMENT INDESIRABLE (AE)

HUG
Hôpitaux Universitaires de Genève

Nom de l'étude

Patient n°
|__|__|__|__|

Date : |__|__|__|__|__|__| Heure : |__|__|__|__|
J J M M A A h h min min

Nature : --

Degré de sévérité : ☐ léger ☐ modéré ☐ sérieux

Evénement indésirable **grave** (SAE) ? ☐ oui ☐ non
Si oui compléter le formulaire (Evénement Indésirable Grave du CRF)
Rappel définitions d'un événement indésirable grave :
☐ Décès (quelle que soit la cause)
☐ Etat critique, pouvant entraîner la mort
☐ Hospitalisation ou prolongation de l'hospitalisation
☐ Séquelles durables (ex : cancer, malformations,…)
☐ Invalidité, handicap transitoire ou non
☐ Intérêt médical particulier (ex:convulsions, développement d'une dépendance à la drogue ou abus de drogue,...)

Traitement entrepris: ☐ aucun
☐ médicament(s) : --

☐ autre : --

En relation avec médicament d'étude:
☐ exclu
☐ pas exclu (effet indésirable médicamenteux-ADR-)
☐ non évaluable

Si ADR, médicament d'étude stoppé ?
☐ oui ☐ non

Issue :
☐ En cours
☐ Résolu
☐ Résolu avec séquelles
☐ Inconnue

Signature de l'investigateur :____________________

Evénement indésirable = Adverse Event (AE)
Evénement indésirable grave = Serious Adverse Event (SAE)
Effet indésirable médicamenteux =Adverse Drug Reaction (ADR)
SUSAR = Suspected Unexpected Serious Adverse Reaction

EVENEMENT INDESIRABLE GRAVE (SAE)

Hôpitaux Universitaires de Genève

Nom de l'étude

Patient n° |__|__|__|__|

Date : |__|__|__|__|__|__| J J M M A A

Heure : |__|__|__|__| h h min min

Nature : --

Cocher la case correspondante :

- ☐ Décès (quelle que soit la cause)
- ☐ Etat critique, pouvant entraîner la mort
- ☐ Hospitalisation ou prolongation de l'hospitalisation
- ☐ Séquelles durables (ex : cancer, malformations,…)
- ☐ Invalidité, handicap transitoire ou non
- ☐ Intérêt médical particulier (ex: convulsions, développement d'une dépendance à la drogue ou abus de drogue,...)

L'évenement indésirable grave est-il **attendu** ?

☐ oui ☐ non

En relation avec le médicament de l'étude :

☐ exclu ☐ pas exclu ☐ non évaluable

Si « pas exclu », déterminer le degré de causalité du médicament d'étude (imputabilité):

- ☐ Certain (1)
- ☐ Probable (2)
- ☐ Possible (3)
- ☐ Improbable (unlikely) (4)
- ☐ Non évaluable (unclassifiable) (5)

Si réponse « non » au caractère attendu et 1, 2 ou 3 à la causalité, c'est 1 **SUSAR** (Suspected Unexpected Serious Adverse Reaction), veuillez informer immédiatement Swissmedic et le CE HUG

Traitement entrepris: ☐ aucun

☐ médicament(s) : --

☐ autre : --

Evénement indésirable = Adverse Event (AE)
Evénement indésirable grave = Serious Adverse Event (SAE)
Effet indésirable médicamenteux =Adverse Drug Reaction (ADR)
SUSAR = Suspected Unexpected Serious Adverse Reaction

EVENEMENT INDESIRABLE GRAVE (SAE)

HUG
Hôpitaux Universitaires de Genève

Nom de l'étude

Patient n°
|__|__|__|__|

L'insu a-t-il été levé ?
☐ oui ☐ non

Si oui, dans quel groupe se trouve le patient :________________________

Si ADR, médicament d'étude stoppé ?
☐ oui ☐ non

Issue :
- ☐ Rétablissement
- ☐ Amélioration
- ☐ Stabilisation
- ☐ En cours
- ☐ Aggravation
- ☐ Séquelles
- ☐ Mort
- ☐ Inconnue

Signature de l'investigateur : _________________________

Evénement indésirable = Adverse Event (AE)
Evénement indésirable grave = Serious Adverse Event (SAE)
Effet indésirable médicamenteux =Adverse Drug Reaction (ADR)
SUSAR = Suspected Unexpected Serious Adverse Reaction

Bernard Favrat & Paul Vaucher
Unité de Médecine et de Psychologie du Trafic
Centre Universiatire Romand de Médecine Légale
Michel-Servet 1
CH-1211 Genève 4

Unitec
Université de Genève
24, Rue du Général Dufour
1211 Genève 4
Switzerland

Genève, le 4 janvier 2013

Chère Madame, Cher Monsieur,

Par la présente, nous, Bernard Favrat né le 05.07.1961, et Paul Vaucher né le 01.09.1975, déclarons renoncer à notre part personnelle des revenus potentiels issus d'une commercialisation éventuelle par l'Université de Genève et/ou du Centre Hospitalier Universitaire Vaudois, ou de leur(s) licencié(s) du logiciel informatique MedDrive que nous avons créé dans le cadre de notre activité professionnelle. MedDrive a fait l'objet d'une annonce de logiciel auprès du bureau de transfert de technologies et de compétences Unitec de l'Université de Genève (723-A600)

Cette décision vise à préserver notre intégrité scientifique et à assurer l'absence de conflits d'intérêts financiers pouvant interférer avec nos recherches et notre promotion de MedDrive. Nous avons conscience que notre décision est irrévocable. Cette décision est faite en connaissance et conscience des conséquences, et en l'absence de toutes pressions de la part de nos collègues, collaborateurs ou hiérarchie.

Bernard Favrat

Genève, le 4 janvier 2013

Paul Vaucher

Genève, le 4 janvier 2013
